# An updated compendium and reevaluation of the evidence for nuclear transcription factor occupancy over the mitochondrial genome

**DOI:** 10.1371/journal.pone.0318796

**Published:** 2025-03-31

**Authors:** Georgi K Marinov, Vivekanandan Ramalingam, William J Greenleaf, Anshul Kundaje

**Affiliations:** 1 Department of Genetics, Stanford University, Stanford, California, United States of America; 2 Center for Personal Dynamic Regulomes, Stanford University, Stanford, California, United States of America; 3 Department of Applied Physics, Stanford University, Stanford, California, United States of America; 4 Chan Zuckerberg Biohub, San Francisco, California, United States of America; 5 Department of Computer Science, Stanford University, Stanford, California, United States of America; Università degli Studi di Milano, ITALY

## Abstract

In most eukaryotes, mitochondrial organelles contain their own genome, usually circular, which is the remnant of the genome of the ancestral bacterial endosymbiont that gave rise to modern mitochondria. Mitochondrial genomes are dramatically reduced in their gene content due to the process of endosymbiotic gene transfer to the nucleus; as a result most mitochondrial proteins are encoded in the nucleus and imported into mitochondria. This includes the components of the dedicated mitochondrial transcription and replication systems and regulatory factors, which are entirely distinct from the information processing systems in the nucleus. However, since the 1990s several nuclear transcription factors have been reported to act in mitochondria, and previously we identified 8 human and 3 mouse transcription factors (TFs) with strong localized enrichment over the mitochondrial genome using ChIP-seq (**Ch**romatin **I**mmuno**p**recipitation) datasets from the second phase of the ENCODE (**Enc**yclopedia **o**f **D**NA **E**lements) Project Consortium. Here, we analyze the greatly expanded in the intervening decade ENCODE compendium of TF ChIP-seq datasets (a total of 6,153 ChIP experiments for 942 proteins, of which 763 are sequence-specific TFs) combined with interpretative deep learning models of TF occupancy to create a comprehensive compendium of nuclear TFs that show evidence of association with the mitochondrial genome. We find some evidence for chrM occupancy for 50 nuclear TFs and two other proteins, with bZIP TFs emerging as most likely to be playing a role in mitochondria. However, we also observe that in cases where the same TF has been assayed with multiple antibodies and ChIP protocols, evidence for its chrM occupancy is not always reproducible. In the light of these findings, we discuss the evidential criteria for establishing chrM occupancy and reevaluate the overall compendium of putative mitochondrial-acting nuclear TFs.

## Introduction

Mitochondria contain their own genome [2] (mtDNA/chrM), usually circular in topology and highly compact, especially in metazoans. The mammalian chrM is around 16-17 kbp in size and encodes for 13 proteins (all of them components of electron transport chains), 22 tRNAs and two rRNAs [3,4]. It has a peculiar compared to the nuclear genome organization and is replicated, transcribed and regulated by its own dedicated set of information processing factors. The 13 genes and two rRNAs are densely packed, with only one notable non-coding region (NCR)—the so-called displacement or D-loop [5]. The D-loop is major the site of replication and transcription initiation, the latter producing long polycistronic transcripts from both strands (referred to as the H- for “heavy” and L- for “light”), and contains three promoters—a light strand (LSP) and two heavy strand (HSP1 and HSP2) promoters [6,7]. Transcription is carried out by the mitochondrial-specific nuclear-encoded POLRMT RNA polymerase [8], with several additional nuclear genome-encoded factors—TFAM [9–11], TFB1M, and TFB2M [12,13]—also involved in the process of initiation [14] (though the evidence for the involvement TFB1M has become much weaker in recent years [15]).

The modern organization of the mitochondrial genome is the result of a combination of extreme reduction and massive endosymbiotic gene transfer (EGT). Mitochondria originated very early in eukaryote evolution as a result of the establishment of endosymbiosis with their prokaryotic ancestor [16–21], which was most likely a member of the *α*-proteobacteria clade [21–24]. Subsequently, the endosymbiont lost the vast majority of its genes, either outright, or through transfer to the nuclear genome, of which some gained protein import signals which allowed redirecting them back into the mitochondrion.

In addition to the well-characterized bona fide mitochondrial transcription and replication factors, since the 1990s there have been reports suggesting that nuclear transcription factors may also moonlight as direct regulators of events in mitochondria [25–28]. These include the glucocorticoid receptor GR [29–32], a 43kDa isoform of the thyroid hormone T_3_ receptor T_3_R*α*1 called p43 [33–36], the CREB TF [37–40], the tumor suppressor transcription factor p53 [41–44], the estrogen receptor ER [45,46], STAT3 [47], AP-1 and PPAR*γ*2 [48–50], as well as MEF2D in mouse [51]. Other nuclear proteins involved in transcriptional regulation have also been reported to play a role in mitochondria, such as the MOF acetyltransferase [52].

However, direct *in vivo* evidence for occupancy of mtDNA by nuclear factors was provided only for a handful of them by these original studies (e.g. CREB [38] and p53 [44], and it was limited to only the D-loop region. The advent of high-resolution techniques for genome-wide profiling of DNA-protein interactions such as ChIP-seq (Chromatin Immunoprecipitation coupled with deep sequencing [53–56]) eventually enabled the direct examination of evidence for mtDNA occupancy by a large number of nuclear TFs.

At the end of the second phase of the ENCODE Project, we and others [27,28] carried out a comprehensive survey of the existing at the time ChIP-seq datasets generated by the ENCODE, mouseENCODE and modENCODE efforts [57–63] in human, mouse, the worm *C. elegans* and the fly *D. melanogaster*. Eight human TFs were identified as showing strong evidence for mtDNA occupancy (JUN, JUND, CEBPB, MAX, MafF, MafK, NFE2 and RFX5), three mouse TFs (MafK, MafF and Usf2), and no fly or worm ones. Furthermore, Blumberg et al. [28] demonstrated directly the localization to mitochondria of JUN and JUND in HepG2 cells using immuno-gold labeling and electron microscopy while Marinov et al. [27] showed MAFK localizing to mitochondria using immunocytochemical staining. Examination of available ChIP-seq data for TFs previously proposed to act in mitochondria (GR, ER*α*, CREB, STAT3, p53) found no putative occupancy sites.

However, these studies did not reveal any obvious mechanisms through which these nuclear TFs might act to regulate mitochondrial transcription, as all the identified ChIP-seq peaks are away from the D-loop in the middle of the transcriptional units. The D-loop itself shows up as “enriched” in practically all ChIP-seq datasets, but this is almost certainly an experimental artifact as the ChIP signal there does not show the characteristics of proper occupancy sites (e.g. it does not display the typical asymmetric read distribution around the binding site; the additional fact that “enrichment” is observed in the great majority of ChIP-seq datasets, something extremely unlikely to reflect true occupancy for all assayed proteins, makes this a clear artifact). Thus, the question about the potential role of nuclear TFs in mitochondria remains open and unresolved [25,26].

With the completion of the third [64] and fourth phases of the ENCODE project, a vastly expanded collection of ChIP-seq datasets has now become available, encompassing an order-of-magnitude larger sampling of the human TF repertoire. Furthermore, many TFs have now been assayed using multiple different reagents or using endogenous tagging, thus potentially providing distinct lines of evidence for mtDNA occupancy, and powerful deep learning-based tools for analyzing the sequence patterns driving TF occupancy and predicting it from sequence have been developed [65]. In this study, we take advantage of these resources, survey the expanded ENCODE collection, and identify 50 nuclear TFs plus two other chromatin proteins exhibiting more or less robustly supported peaks over chrM. On the other hand, the picture revealed by the expanded collection is more complicated than previously perceived as in many cases occupancy profiles are not replicated in all cases where multiple immune reagents have been used to assay the same TF. We discuss the currently most reliable set of mtDNA-associated nuclear TFs, as well as the evidential criteria for establishing chrM occupancy using ChIP and other experimental methods.

## Materials and methods

All custom Python scripts used to carry out computational processing can be found at https:// github.com/georgimarinov/GeorgiScripts.

### ChIP-seq data processing

Raw sequencing reads for transcription factor ChIP-seq datasets were downloaded from the ENCODE Consortium Portal [66] (https://www.encodeproject.org/; data current as of May 1st 2022). Reads were aligned using Bowtie [67] (version 1.1.1) as 1 × 36mers against an index containing the mitochondrial genome, with the following settings ‘‘-v 2 -k 2 -m 1 -t --best --strata’’.

The hg38 version of the *Homo sapiens* genome was used for all analysis.

### Screening for TF occupancy over the mitochondrial genome

We then generated plus- and minus-strand coverage tracks over chrM for all datasets and made Circos [68] plots for each such pair. These Circos plots were manually examined to identify likely TF occupancy events and to screen out potentially artifactual high ChIP signal localization events that do not display the expected asymmetric pattern around true occupancy sites.

### Mappability track generation

Mappability was assessed as follows. Sequences of length *N* bases were generated starting at each position in the mitochondrial genome. The resulting set of “reads” was then mapped against the same bowtie index used for mapping real data. Positions covered by *N* reads were considered fully mappable. In this case, *N* = 36 as this is the read length for most of the sequencing data analyzed in this study.

### BPNet model training and predictions

Uniformly processed ChIP-seq datasets were downloaded from the ENCODE portal. For experiments utilizing paired-end sequencing, PCR duplicates were eliminated. However, for experiments with single-end sequencing, all reads were retained due to the absence of a dependable method for removing duplicates without sacrificing significant genuine signal. Then we generated base resolution signal tracks from the 5’ end of the mapped reads.

The BPNet model architecture and the training approach were adapted from [69]. The model was designed to accept a one-hot-encoded input DNA sequence of 2,114 base pairs, predicting signals at a 1,000-basepair output window. Additionally, a control input DNA track was also provided to predict the residuals of the ChIP signal from the control track using input sequences. The model outputs comprised of a profile output and a total *log* counts output for 1,000-bp windows.

The model architecture consisted of nine consecutive convolutional layers with ReLU activation. The initial convolutional layer featured a filter size of 21 with no dilation, while subsequent layers utilized filter sizes of 3 with a stride of 1 and increasing dilation rates (power of two) in subsequent layers. Each layer had 64 filters, with residual connections between the convolution layers and zero padding across all layers. Profile prediction was calculated by passing the last dilated convolution output to another convolutional layer with a kernel size of 75 (stride 1) and no padding and no activation, followed by stacking with the control profile tracks and feeding to a final convolutional filter of size 1, operating on one base of the logits and control profiles at a time to generate the predicted profile logits. The *log* of total read counts was computed by performing global average pooling on last dilated convolution output layer. The pooled output was then fed to a dense layer and then concatenated with the *log* of the total counts across both strands from the control experiment, and then further processed through a final dense layer predicting the *log* of the total counts across both strands. The predicted profile logits were converted into probabilities using a single softmax function, generating the profile predictions for both strands, and a negative log-likelihood loss was calculated. Mean squared error of the *log* of total counts across both strands was also computed.

Training epochs comprised of IDR [70]-thresholded (Irreproducibility Discovery Rate) peaks and non-peak regions (selected to match the GC content of the peak regions) at a 3:1 ratio. Outlier peak regions with signals exceeding 1.2 times the 99th quantile were removed. Additionally, peak sequences were randomly jittered up to 128 bp to augment positive examples. Reverse-complement augmentation was also employed during training. Models were trained and evaluated using 5-fold cross-validation by chromosomes, ensuring no overlap between training, validation, and test sets. Predictions were averaged across both the forward and reverse-complement of the input sequences. All BPNet models and associated outputs will be released as part of the ENCODE Consortium Phase 4 flagship manuscript.

Trained models were then used to generated predicted occupancy profiles for both strands over the mitochondrial genome by splitting the mitochondrial genome into 2,114 bp tiles.

In some cases, we did not obtain usable predictions over chrM. This is generally due to failing to train good models, primarily due to less-than-ideal quality of the input datasets—too few peaks, poor signal-to-noise ratio, strong non-specific enrichment, etc.

## Results

### Evaluating the evidence for mitochondrial occupancy of nuclear TFs in the expanded ENCODE ChIP-seq collection

According to the latest census of human transcription factors [1], the human genome encodes 1,742 sequence-specific TFs, belonging to  ∼ 60 different families defined by their DNA binding domains (DBDs).

Our previous analysis of the dataset collection generated as part of the second phase of the ENCODE Project encompassed a total of 151 transcription factors, which represents less than 10% of the total. After the fourth phase of ENCODE, the number of available datasets is now greatly expanded and covers  ∼ 44% of the known TFs.

**Fig 1:**
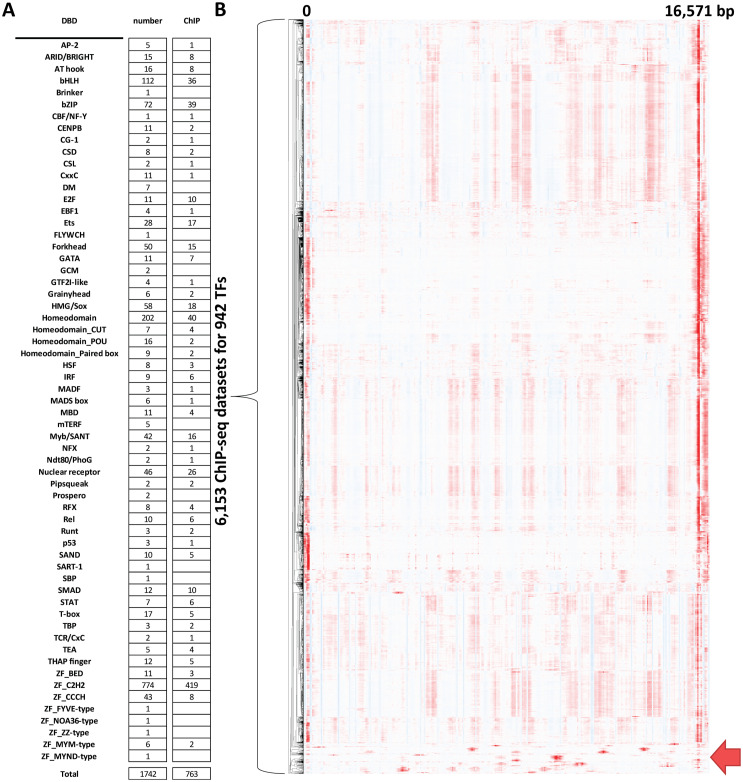
Global assessment of the evidence for association of human nuclear TFs with the mitochondrial genome. (A) Summary of the known human TFs and available ENCODE TF ChIP datasets. The TF classification of human TFs of Lambert et al. 2018 [1] was followed. (B) Hierarchical clustering of ChIP-seq profiles over the mitochondrial genome for 6,513 TF ChIP-seq datasets. Datasets that show evidence for non-artefactual association with mitochondrial DNA are highlighted at the bottom.

In order to evaluate putative physical associations of nuclear TFs with the mitochondrial genome (and also reevaluate previous observations), we examined 6,153 ChIP-seq datasets for 942 targets, of which 763 are sequence-specific TFs [1] (Fig 1A). The additional 179 are non-sequence specific chromatin proteins, such as histone modifying enzymes and chromatin remodelers.

The most recent ENCODE collection is also not merely a quantitative expansion. Multiple TFs have been assayed with different antibodies, which is extremely valuable because, especially when the antibodies are polyclonal, the possibility that mtDNA ChIP-seq peaks are the result of non-specific pull down can never be excluded (although that would still mean that some previously not known to do so protein is specifically occupying those regions of the mitochondrial genome). Many of the newly available TFs, but also some that have been mapped previously, have now been assayed using endogenous tagging with FLAG, GFP or HA tags, most commonly using CRISPR epitope tagging (CETCH-seq [71]) and site-specific recombination [72]. We note that in CETCH-seq tagging is carried out at the stop codon of the gene, i.e. at its C-terminus, thus it is unlikely that it would affect import into mitochondria, which is classically mediated by N-terminal targeting sequences [73,74]; however, that can nevertheless not be completely excluded as a possibility.

We updated our pipeline for analyzing mitochondrial occupancy in several ways in order to allow for more comprehensive characterization of the current TF compendium.

First, we previously mapped reads against a joint nuclear and mitochondrial genomic index, and excluded all reads mapping to multiple locations within that combined genomic space. A substantial portion of the human chrM in the context of the hg38 assembly is affected by such mappability limitations (S1 Text, S1 Fig). However, because of the very high copy number of mitochondrial genomes in a given cell relative to the diploid nuclear genome [75], peaks observed over chrM are nearly always much stronger than even the very top nuclear ChIP-seq peaks, as previously demonstrated [27]. Furthermore, other hallmarks of TF occupancy, such as chromatin accessibility peaks and high levels of histone marks associated with active regulatory elements, which would be expected if chrM peaks arose from mitochondrial sequences that have been inserted in the nuclear genome (so called NUMTs [76]), are not seen over the peaks observed over the mitochondrial genome. This makes it highly unlikely that they arise from nuclear TF occupancy over NUMTs. For these reasons, we now evaluate putative mitochondrial occupancy based on read alignments generated entirely in mitochondrial space.

Second, in our past work we sought corroborating evidence for the observed ChIP-seq profiles in the presence or absence of the cognate sequence recognition motifs for each TF. However, these motifs are often very short and degenerate, and thus only a small fraction of them is actually occupied in cells. In this work, we leverage the power of interpretative deep learning models to generate more reliable and specific predictions of TF occupancy profiles over chrM, which we then compare to experimental measurements. We use the state-of-the-art BPNet [69] profile models, which take as input genomic sequence and the forward- and reverse-strand ChIP-seq profiles, and then learn to predict these profiles as a function of genomic sequence. As part of the overall ENCODE effort, we have trained such models over the nuclear genome for all TFs for which data is available, and we used these models to predict chrM ChIP-seq profiles (see Methods for details).

Fig 1B shows the observed chrM profiles for all 6,153 datasets. As discussed in our previous work [27], the D-loop region appears as strongly “enriched” in nearly all ChIP datasets; this is certainly an artifact in almost all cases because the observed forward- and reverse-strand profiles do not exhibit the expected from true occupancy asymmetry around a punctate binding site [77–80]. It is most likely that the unique triple-stranded structure of the D-loop results in preferential enrichment in sequencing libraries. We also observe a few regions of weakly elevated signal in the middle of chrM, which are also present in the majority of datasets, and are also unlikely to represent true occupancy events.

Disregarding these signals, we find some evidence for chrM occupancy for 50 sequence-specific TFs, which we discuss in detail below. In addition, two of the 179 non-sequence specific chromatin proteins also showed evidence for putative association with mtDNA.

### bZIP TFs

The TF family with the largest and most notable set of members with strong chrM peaks is the bZIP (Basic Leucine Zipper) domain-containing proteins. In humans, 72 such TFs are annotated in the genome, and for 39 of them there is ENCODE ChIP-seq data.

**Fig 2:**
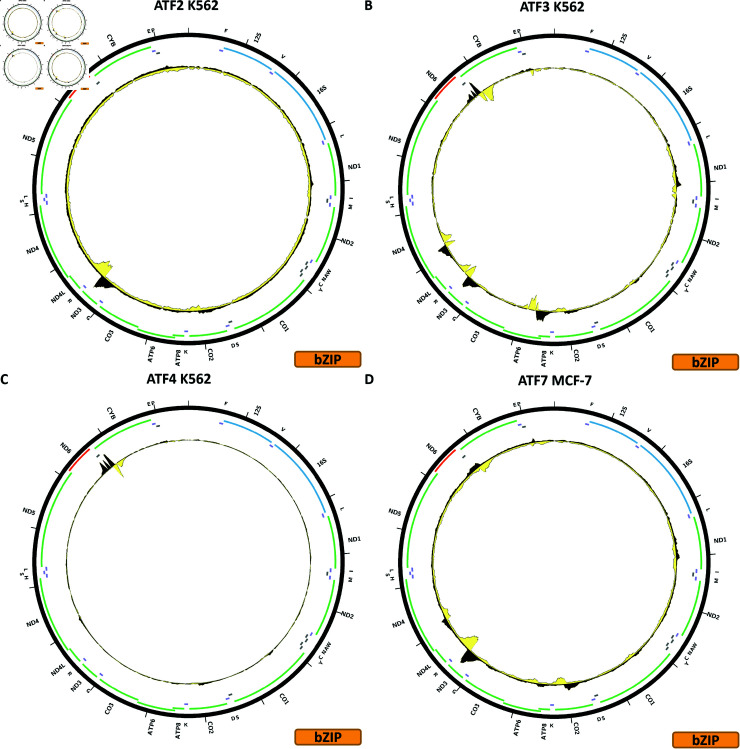
Evidence for mitochondrial genome occupancy by nuclear transcription factors. Black and yellow tracks show the forward- and reverse-strand ChIP-seq coverage over chrM. (A) ATF2 (bZIP); (B) ATF3 (bZIP); (C) ATF4 (bZIP); (D) ATF7 (bZIP).

**Fig 3:**
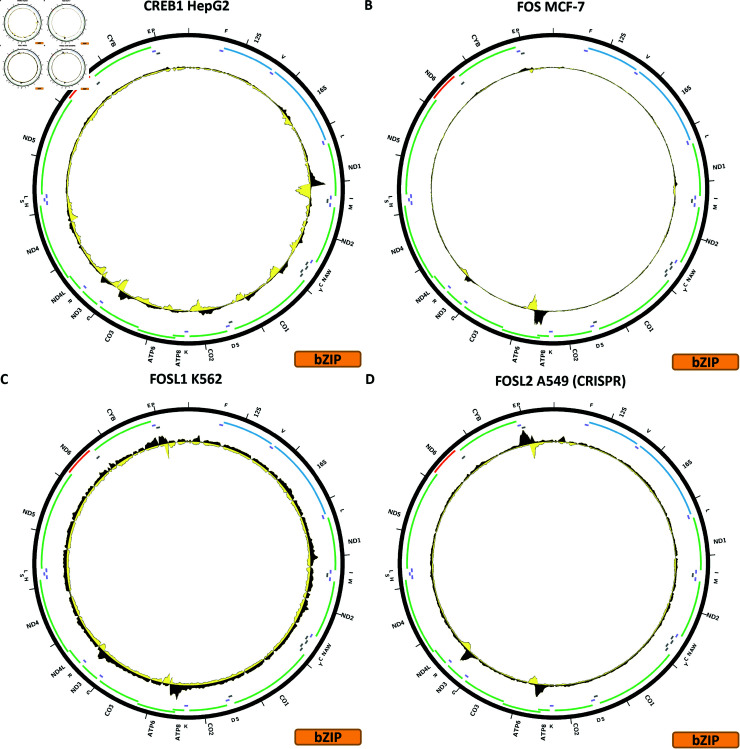
Evidence for mitochondrial genome occupancy by nuclear transcription factors. Black and yellow tracks show the forward- and reverse-strand ChIP-seq coverage over chrM. (A) CREB1 (bZIP); (B) FOS (bZIP); (C) FOSL1 (bZIP); (D) FOSL2 (bZIP).

**Fig 4:**
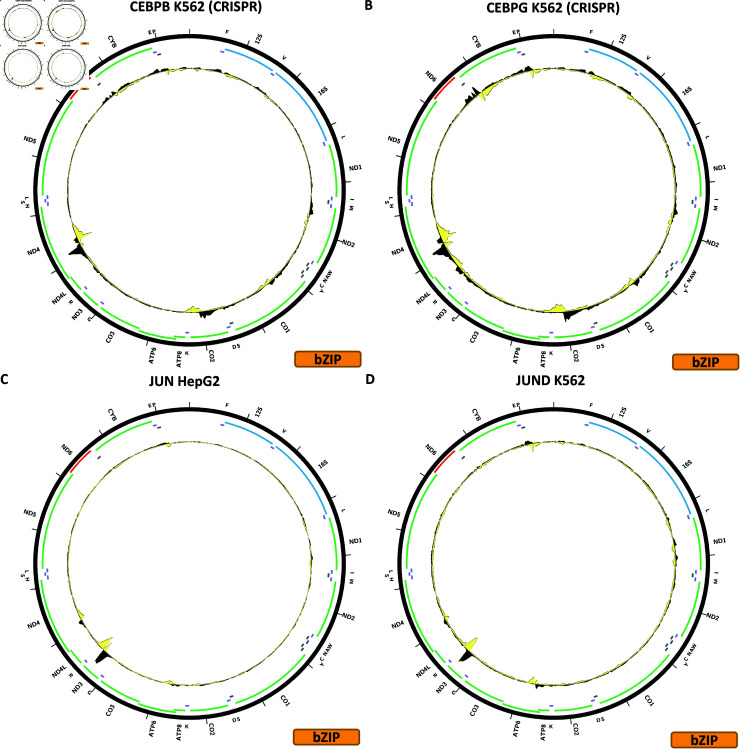
Evidence for mitochondrial genome occupancy by nuclear transcription factors. Black and yellow tracks show the forward- and reverse-strand ChIP-seq coverage over chrM. (A) CEBPB (bZIP); (B) CEBPG (bZIP); (C) JUN (bZIP); (D) JUND (bZIP).

**Fig 5:**
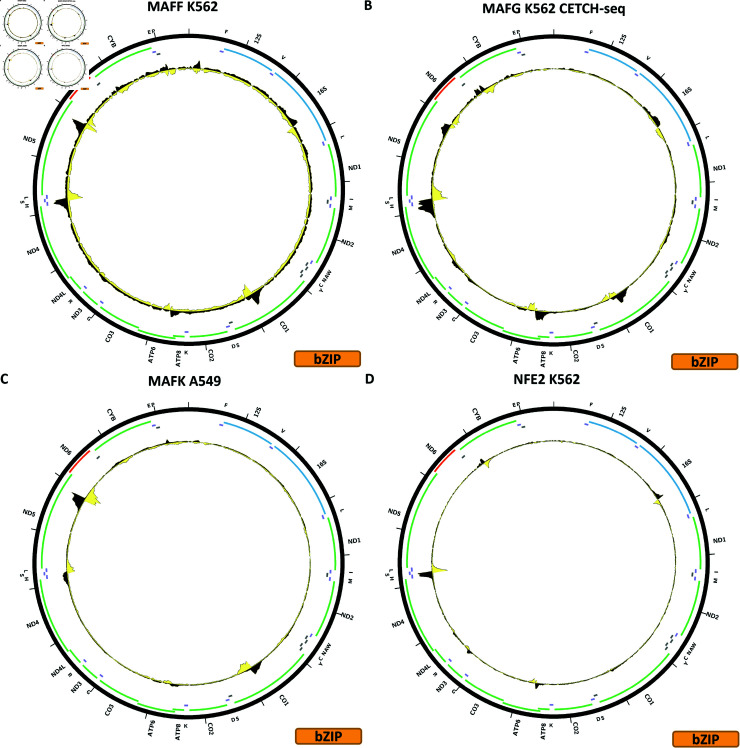
Evidence for mitochondrial genome occupancy by nuclear transcription factors. Black and yellow tracks show the forward- and reverse-strand ChIP-seq coverage over chrM. (A) MAFF (bZIP); (B) MAFG (bZIP); (C) MAFK (bZIP); (D) NFE2 (bZIP).

**Fig 6:**
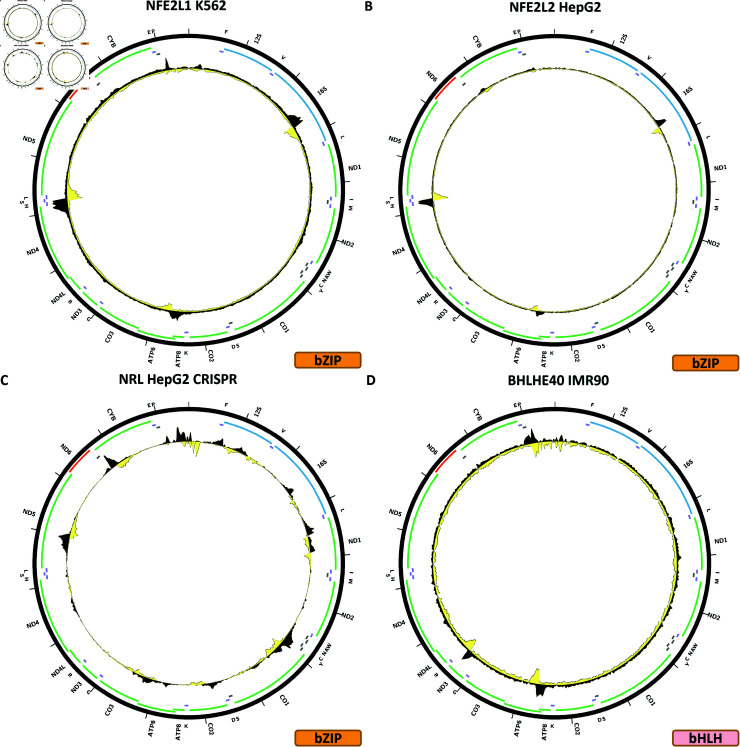
Evidence for mitochondrial genome occupancy by nuclear transcription factors. Black and yellow tracks show the forward- and reverse-strand ChIP-seq coverage over chrM. (A) NFE2L1 (bZIP); (B) NFE2L2 (bZIP); (C) NRL (bZIP); (D) BHLHE40 (bHLH).

Remarkably, nearly half of them—19/39—exhibit evidence for mtDNA occupancy (Figs 2–6A–C and S1 Text, S2–S22 Figs). These TFs are ATF2, ATF3, ATF4, ATF7, CREB1, FOS, FOSL1, FOSL2, CEBPB, CEBPG, JUN, JUND, MAFF, MAFG, MAFK, NFE2, NFE2L1, NFE2L2, and NRL.

Fig 2A shows the chrM ChIP-seq profile for the ATF2 TF in the K562 cell line, revealing a strong peak, with the classic strand asymmetry features of true sequence-specific TF occupancy, in the region around the *MT-ND3* and *MT-ND4L* genes and the *MT-TR* tRNA gene. The BPNet-predicted profile corroborates the existence of such a peak, although BPNet also predicts high ChIP-seq signal in several other locations, which is not observed in the actual data (S1 Text, S2B Fig). The same peak is observed in HepG2 cells using the same antibody (S1 Text, S2E Fig), and in site-recombination-tagged experiment in the HEK293 cell line (S1 Text, S2D Fig). It is not observed in H1-hESC using a different antibody (S1 Text, S2C Fig), and also in a CRISPR-mediated endogenous tagging experiment in HepG2 cells (S1 Text, S2F Fig). Thus, for ATF2 we see evidence for occupancy using two different reagents (an *α*-ATF2 antibody and GFP-tagging), but not with another *α*-ATF2 antibody and FLAG-tagging.

Fig 2B shows the chrM ChIP-seq profile for the ATF3 TF in the K562 cell line. In this case, at least four strong peaks are observed—the same one around *MT-ND3*/*MT-ND4L* seen for ATF2, but also one over *MT-ATP6*, one over *MT-ND4*, and another over *MT-CYB*. All these peaks are corroborated by BPNet predictions (S1 Text, S3B Fig), although here too BPNet predicts additional occupancy peaks. However, none of the experiments in other cell lines—H1-hESC, HCT116, HepG2, A549, GM12878, liver, and K562 again—carried out with a different antibody exhibit these peaks (S1 Text, S3C–S3I Fig), and neither does CRISPR-mediated FLAG-tagging (S1 Text, S4J Fig).

Fig 2C shows the chrM ChIP-seq profile for the ATF4 TF in the K562 cell line. In this case we observe a single strong peak, at the same location as the ATF3 peak over the *MT-CYB* gene. Unfortunately, we were not able to train a good model for this TF, thus we do not have BPNet predictions over chrM for it (S1 Text, S4B Fig). This peak is not seen in a CRISPR-mediated FLAG-tagging experiment in HepG2 cells (S1 Text, S4C Fig).

Fig 2D shows the chrM ChIP-seq profile for the ATF7 TF in the MCF-7 cell line. Its profile over chrM is similar to that of ATF3 (Fig 2B), with four peaks. BPNet models corroborate the strong peak over *MT-ND3*/*MT-ND4L*, but are less concordant elsewhere in the genome. These peaks are also seen in GM12878 and K562 ChIP-seq experiments generated with the same antibody (S1 Text, S5C–S5D Fig), but not in HepG2 ChIP-seq carried out with a different antibody (S1 Text, S5E Fig).

Fig 3A shows the chrM ChIP-seq profile for the CREB1 TF in the HepG2 cell line. CREB1 is notable for having been previously proposed to localize to mitochondria and play a functional role there [38,40], and specifically to bind to the D-loop [39]. Just as in our previous effort [27], we see no evidence that is unlikely to be an artifact for D-loop occupancy, but we observe a strong peak over the *MT-ND1* gene, another one over *MT-CO3* and several weaker others elsewhere in the genome. These match BPNet predictions qualitatively, but the magnitudes of observed and predicted signals differ significantly (S1 Text, S6B Fig). The putative occupancy profiles are replicated in MCF-7 cells using the same antibody (S1 Text, S6D Fig), in CRISPR FLAG-tagged HepG2 and GM23338 cells (S1 Text, S6C and S6E Fig), and also in K562 cells using a different antibody (S1 Text, S6F Fig). However, the latter antibody was also used in datasets in GM12878, H1-hESC and Ishikawa cells (S1 Text, S6G–S6I Fig) resulting in a flat profile over chrM, as is the case with a CETCH-seq experiment in K562 cells (S1 Text, S6J Fig).

Fig 3B shows the chrM ChIP-seq profile for the FOS TF in the MCF-7 cell line. Two peaks are observed—a strong one over the *MT-ATP6* gene and a weaker one at the same location as the ATF2, ATF3 and ATF7 peaks over *MT-ND3*/*MT-ND4L*. Both are matched by BPNet predictions while several other BPNet-predicted peaks are not observed in the data (S1 Text, S7B Fig). The pattern is replicated in K562 cells using the same antibody (S1 Text, S7C Fig), but not in IMR90, endothelial cells of the umbilical vein, GM12878 or HeLaS3, all using the same antibody (S1 Text, S7D–S7G Fig).

Fig 3C shows the chrM ChIP-seq profile for the FOSL1 TF in the K562 cell line; this experiment uses GFP-tagged FOSL1. The same two peaks as for FOS are observed, but neither is particularly strong. BPNet predicts a large number of peaks all over mtDNA, which are not seen in the data (S1 Text, S8B Fig). These peaks are not replicated by K562 CETCH-seq, HepG2 CETCH-seq, and ChIP-seq using an *α*-FOSL1 antibody in H1-hESC and HCT116 (S1 Text, S8C–S8F Fig).

Fig 3D shows the chrM ChIP-seq profile for the FOSL2 TF in the A549 cell line, using CRISPR-tagged cells. Again, the same two peaks are observed as for FOS and FOSL1. These are also predicted by BPNet (S1 Text, S9B Fig), but the strongest BPNet prediction—over *MT-CYB* is not observed in the data. However, other experiments using two different antibodies in A549, MCF-7, HepG2 and SK-N-SH as well as CETCH-seq in MCF-7 and HepG2 do not shows these peaks (S1 Text, S9C–S9J Fig).

Fig 4A shows the chrM ChIP-seq profile for the CEBPB TF in the K562 cell line, using CRISPR-tagged cells. A strong peak is observed over the *MT-ND4* gene, and a weaker one over *MT-CO2*, as well as a few other weak peaks. These are corroborated by BPNet (S1 Text, S10B Fig); in fact BPNet predicts two distinct binding sites over *MT-ND4* and two peaks associated with strand asymmetry are also seen in the CETCH-seq data. BPNet also predicts numerous other peaks that are not observed experimentally. A large number of different additional experiments are available for CEBPB (S1 Text, S10C–S10J and S11 Figs), using two different antibody lots. The observed putative mtDNA occupancy is replicated in IMR90 (S1 Text, S10D Fig), HeLaS3 (S1 Text, S10F Fig), HepG2 (S1 Text, S10J Fig), A549 (S1 Text, S11E Fig), but not in other experiments for HepG2 and A549 or the other cell lines—MCF-7, HCT116, Ishikawa, H1-hESC, GM12878 and non-tagged K562—that have been assayed.

Fig 4B shows the chrM ChIP-seq profile for the CEBPG TF in the K562 cell line, using CRISPR-tagged cells. A similar pattern to CEBPB is observed, but with a stronger peak over the *MT-CYB* gene. BPNet predictions match the observed profile (S1 Text, S12B Fig). However, CRISRP-tagged MCF-7 and HepG2 cells do not show these peaks (S1 Text, S12C and S12D Fig).

Fig 4C shows the chrM ChIP-seq profile for the JUN TF in the HepG2 cell line. Two strong peaks are observed for JUN—one over the same *MT-ND3MT-ND4L* region as seen for many other bZIP factors, and another over *ND4*. A weaker peak is seen over *MT-ATP6*. BPNet predicts all these peaks, as well as many others (S1 Text, S13B Fig). These peaks are also observed in endothelial cells of the umbilical veins (S1 Text, S13C Fig), where it is to be noted that the *MT-ATP6* peak is stronger than the *MT-ND4L* one, but not in any of the other cell lines assayed—MCF-7, HeLa-S3, A549, K562, H1-hESC and in a HepG2 CETCH-seq sample (S1 Text, S13D–S13I Fig). All of these ChIP-seq experiments were carried out with the same antibody. JUN was one of the factors whose presence in mitochondria was conclusively confirmed through immunogold electron microscopy previously [28], thus the discrepancy between HepG2 ChIP-seq and CETCH-seq and it being observed only in two seemingly unrelated cell lines and not in any of the others are particularly puzzling observations.

Fig 4D shows the chrM ChIP-seq profile for the JUND TF in the K562 cell line. The same three peaks are observed as for JUN, and these are also corroborated by BPNet predictions (S1 Text, S14B Fig). Here too we observed discordance in the available datasets as these peaks are also seen in HepG2 (S1 Text, S14D Fig) and SK-N-SH (S1 Text, S14H Fig) cells, but not in any of the other ENCODE experiments for JUND—HeLaS3, GM12878, HCT116, H1-hESC, liver, MCF-7, T47D, A549, and most puzzling, additional datasets in K562, HepG2 and SK-N-SH (S1 Text, S14C, S14E–S14G, S14I and S15 Figs). All of these experiments were carried out with the same Santa Cruz Biotech sc-74 antibody, except for the K562 experiments, both of which used GFP-tagged JUND. However, this antibody is polyclonal and there is no information available whether the same lot was used. It is possible the discrepancy arises as a result of lot differences; the other possibility is that the experimental protocols used are not the same as the discordant samples arise from two different production labs. As is the case with JUN, JUND’s presence in mitochondria was previously verified by immunogold electron microscopy in HepG2 cells [28].

Fig 5A shows the chrM ChIP-seq profile for the MAFF TF in the K562 cell line. Several peaks are observed—over the *MT-CO1* and *MT-ND5* genes as well as over the tRNA cluster between *MT-ND4* and *MT-ND5*. These are also predicted by BPNet (S1 Text, S16B Fig) together with multiple other peaks not observed in the ChIP data. The first two peaks are also observed in HepG2 and HeLa-S3 cells (all experiments carried out with the same antibody) but the latter is not.

Fig 5B shows the chrM CETCH-seq profile for the MAFG TF in the K562 cell line. The three peaks observed for MAFF are also present in the MAFG profile, but in addition peaks are present over the *MT-CYB* and *MT-ATP6* genes as well as a weaker one over the 16S rRNA. These peaks are corroborated by BPNet predictions (S1 Text, S17B Fig), but are not seen in a HepG2 CETCH-seq experiment (S1 Text, S17C Fig).

Fig 5C shows the chrM ChIP-seq profile for the MAFK TF in the A549 cell line. The same peaks are observed as those for MAFF, and they are corroborated by BPNet predictions (S1 Text, S18B Fig). They are also observed in GM12878, IMR-90, HeLa-S3, K562, MCF-7 and HepG2 cells, but not in H1-hESC cells (S1 Text, S18C–S18I Fig). Of note, they are seen in datasets generated with two different antibodies, and MAFK was previously shown to localize to mitochondria using immunocytochemical staining.

Fig 5D shows the chrM ChIP-seq profile for the NFE TF in the K562 cell line. Five peaks are observed—over the 16S rRNA gene, over *MT-ATP6*, over *MT-ND3*, over the tRNA cluster between *MT-ND4* and *MT-ND5*, and over *MT-CYB*. These are all sites where peaks are seen also for other bZIP factors. Most of them are predicted by BPNet (S1 Text, S19B Fig), and they also seen in K562 CETCH-seq experiment (S1 Text, S19C Fig). On the other hand, CETCH-seq in HepG2 (S1 Text, S19D Fig) and ChIP-seq in GM12878 generated using a different antibody (S1 Text, S19E Fig) show no peaks.

Fig 6A shows the chrM ChIP-seq profile for the NFE2L1 TF in the K562 cell line. Three strong peaks are observed in this case—over the 16S rRNA gene, over *MT-ATP6*, and over the tRNA cluster between *MT-ND4* and *MT-ND5*. BPNet predicts the latter two but not the one over the 16S rRNA (S1 Text, S20B Fig). A HepG2 CETCH-seq experiment does not exhibit the same pattern (S1 Text, S20C Fig).

Fig 6B shows the chrM ChIP-seq profile for the NFE2L2 TF in the K562 cell line. It is very similar to what is observed for NFE2L1, and in this case too BPNet does not predict a 16S rRNA peak (S1 Text, S21B Fig). These peaks are also seen in IMR-90 cells (S1 Text, S21C Fig) and weakly in A549 and HeLaS3 cells (Fig 21D and 21E), using the same antibody for all experiments.

Fig 6C shows the chrM ChIP-seq profile for the NRL TF in a HepG2 CETCH-seq experiment. In this case, multiple, and often potentially complex multisummit peaks are observed all over the genome. They generally match BPNet predictions (S1 Text, S22B Fig).

### bHLH TFs

The second major group of TFs exhibiting evidence for mtDNA occupancy are the basic helix–loop–helix (bHLH) transcription factors. Of 122 bHLH factors annotated in the genome, ChIP-seq data is available for 36, of which peaks over chrM are observed for four—BHLHE40, MAX, MITF, and SREBF1.

Fig 6D shows the chrM ChIP-seq profile for the BHLHE40 TF in the K562 cell line. Two peaks are observed—over the *MT-ATP6* gene and over *NT-ND3*/*MT-DN4L*. However, the observed profile does not match the BPNet predicted one (S1 Text, S23B Fig), and is also not seen in any other cell line (S1 Text, S23C–S23G Fig). In the GM12878 cell line a different peak is observed over the *MT-ND5* gene (S1 Text, S23D Fig); the same antibody was used for both the IMR90 and GM12878 experiments, but a different antibody was used in A549 and HepG2 cells.

**Fig 7:**
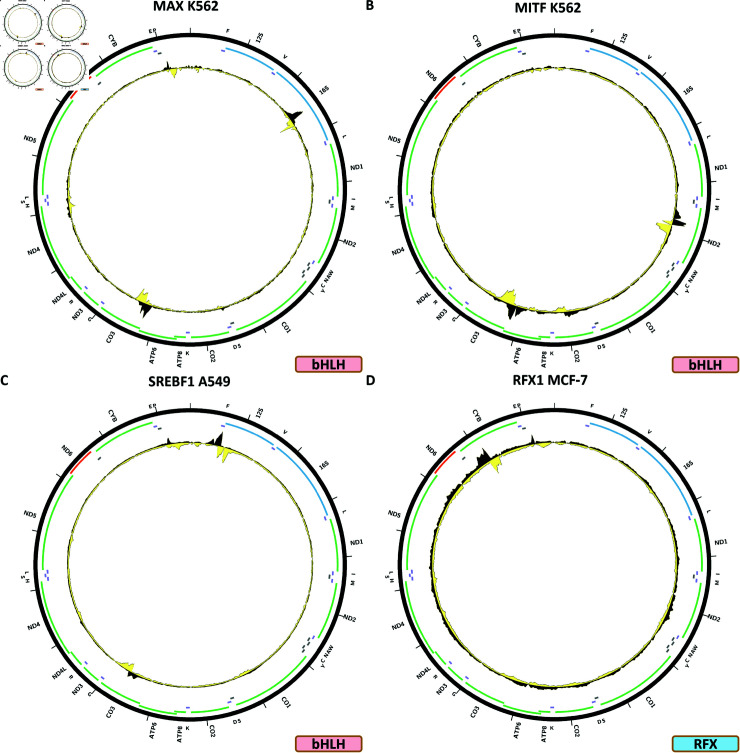
Evidence for mitochondrial genome occupancy by nuclear transcription factors. Black and yellow tracks show the forward- and reverse-strand ChIP-seq coverage over chrM. (A) MAX (bHLH); (B) MITF (bHLH); (C) SREBF1 (bHLH); (D) RFX1 (RFX).

Fig 7A shows the chrM ChIP-seq profile for the MAX TF in the K562 cell line. Strong peaks are observed over the 16S rRNA gene and over *MT-CO3*, which are also predicted by BPNet (S1 Text, S24B Fig) together with a number of other peaks not seen in the data. Many different additional experiments are available for MAX (S1 Text,s S24C–S24J and S25 Figs)—all generated with the same antibody, but including experiments from different production groups. These peaks are also seen in endothelial cells of umbilical vein (S1 Text, S24F Fig) and H1-hESC (S1 Text, S24J Fig), generated by two different productions groups, but not in the rest of the experiments—A549, HepG2 (ChIP and CETCH-seq), GM12878, HCT116, HeLaS3, Ishikawa, liver, MCF-7 and SK-N-SH.

Fig 7B shows the chrM ChIP-seq profile for the MITF TF in the K562 cell line. Peaks are observed over the *MT-ND2* gene and on the boundary between *MT-ATP6* and *MT-CO3*. However, these do not match the predicted BPNet profile (S1 Text, S21B Fig).

Fig 7C shows the chrM ChIP-seq profile for the SREBF1 TF in the A549 cell line. In this case, two potential peaks are seen at the very beginning of the 12S rRNA gene and another one over the *MT-C03* gene. These are predicted by BPNet (S1 Text, S27B Fig), together with many other peaks not observed in the data.

### RFX TFs

The human genome encodes eight RFX TFs, of which ChIP-seq data now exists for four. Two of these shows evidence for mtDNA occupancy—RFX1 and RFX5.

Fig 7D shows the chrM ChIP-seq profile for the RFX1 TF in the MCF-7 cell line. One peak is observed—over the *MT-CYB* gene—where multiple peaks summits are also predicted by BPNet (S1 Text, S28B Fig). This profile is also observed in K562 cells (S1 Text, S28C Fig), but not in HepG2 (S1 Text, S28D Fig).

**Fig 8:**
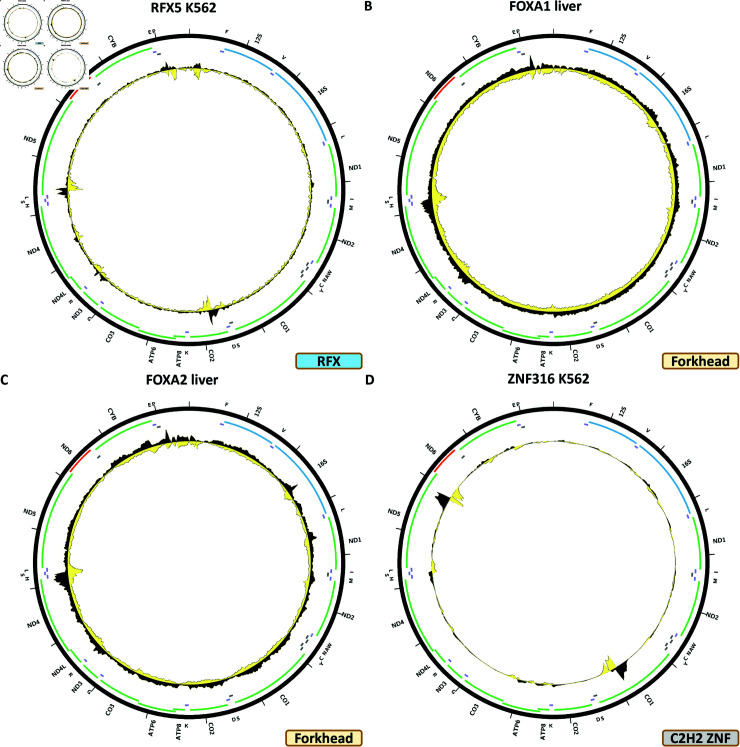
Evidence for mitochondrial genome occupancy by nuclear transcription factors. Black and yellow tracks show the forward- and reverse-strand ChIP-seq coverage over chrM. (A) RFX5 (RFX); (B) FOXA1 (Forkhead); (C) FOXA2 (Forkhead); (D) ZNF316 (C2H2 ZNF).

Fig 8A shows the chrM ChIP-seq profile for the RFX5 TF in the K562 cell line. Three areas of elevated signal are observed—over *MT-CO2*, in the beginning of *ND5*, and in the *MD-ND3*/*MD-ND4L* region. BPNet predicts multiple strong peaks (S1 Text, S29B Fig), two of which match the *MT-CO2* and *MD-ND3*/*MD-ND4L* peaks, but not the *ND5* one. This pattern is also seen in IMR90 cells (S1 Text, S29C Fig). In HepG2 cells, a different profile is observed—a peak over the *MT-ND1* gene (S1 Text, S29D Fig). No peaks are seen in the available other experiments—SK-N-SH, GM12878, H1-hESC, A549, HeLa-S3, MCF-7 (S1 Text, S29E–S29J Fig), all of which were generated using the same antibody.

### Forkhead TFs

A total of 50 forkhead TFs are encoded in the human genome, for which 15 have been assayed by ENCODE. Two of them—FOXA1 and FOXA2—show putative evidence for mtDNA occupancy.

Fig 8B shows the chrM ChIP-seq profile for the FOXA1 TF in liver. A peak is observed over the tRNA cluster between *MT-ND4* and *MT-ND5*, which is also predicted by BPNet (S1 Text, S30B Fig), together with many other peaks not observed in the data. However, HepG2 ChIP-seq carried out with the same antibody does not show the same peak (S1 Text, S30D Fig), and neither does HepG2 CETCH-seq (S1 Text, S30E Fig) nor do ChIP-seq datasets for A549, MCF-7 and K562 cells generated using two other different antibodies (S1 Text, S30C and S30D and S30F–S30H Fig).

Fig 8C shows the chrM ChIP-seq profile for the FOXA2 TF in liver. The same peak is observed as for FOXA1, but it is not strongly predicted by BPNet (S1 Text, S31B Fig). As with FOXA1, it is not seen in cell line data—A549 ChIP-seq, HepG2 CETCH-seq and HepG2 ChIP-seq (S1 Text, S31C–S31E Fig).

### C2H2 zinc finger TFs

The largest TF family in mammals is the C2H2 zinc finger factors. The human genome encodes 774 of these, of which 419 have been now assayed by ENCODE, and 12 show some evidence for mtDNA occupancy—DZIP1, HIVEP1, ZNF225, ZNF274, ZNF350, ZNF598, ZNF768, ZNF839, ZNF891, ZNF263, ZNF280B, ZNF316. Most of these experiments have been carried out using endogenous epitope tagging as specific antibodies for most ZNFs are not available.

Fig 8D shows the chrM ChIP-seq profile for the ZNF316 TF in the K562 cell line. Two strong peaks are observed—over the *MT-CO1* and *MT-ND5* genes, and several weaker ones elsewhere around chrM. These are predicted by BPNet models (Fig 32B). However, a second K562 experiment, carried out by the same production group but with a different antibody does not exhibit any peaks over chrM.

**Fig 9:**
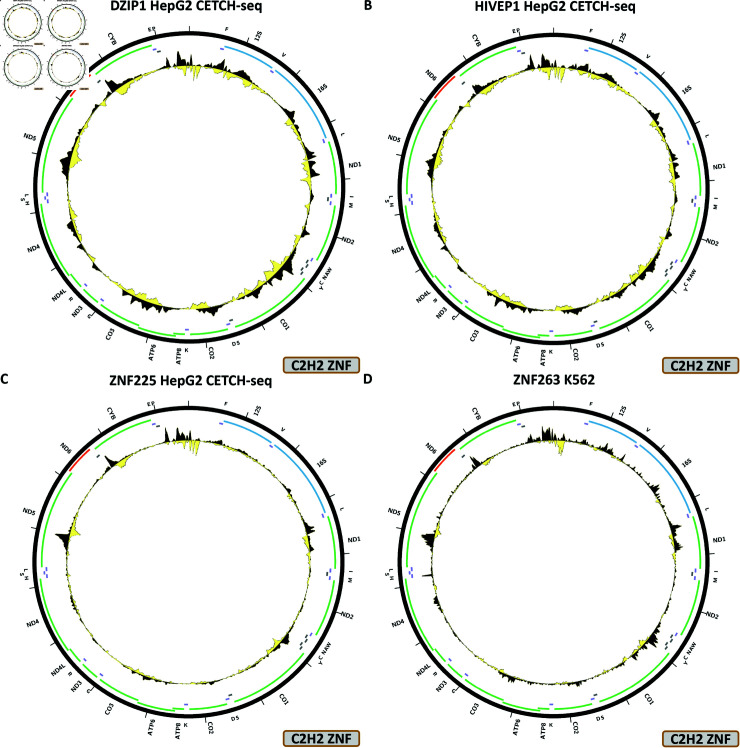
Evidence for mitochondrial genome occupancy by nuclear transcription factors. Black and yellow tracks show the forward- and reverse-strand ChIP-seq coverage over chrM. (A) DZIP1 (C2H2 ZNF); (B) HIVEP1 (C2H2 ZNF); (C) ZNF225 (C2H2 ZNF); (D) ZNF263 (C2H2 ZNF).

**Fig 10:**
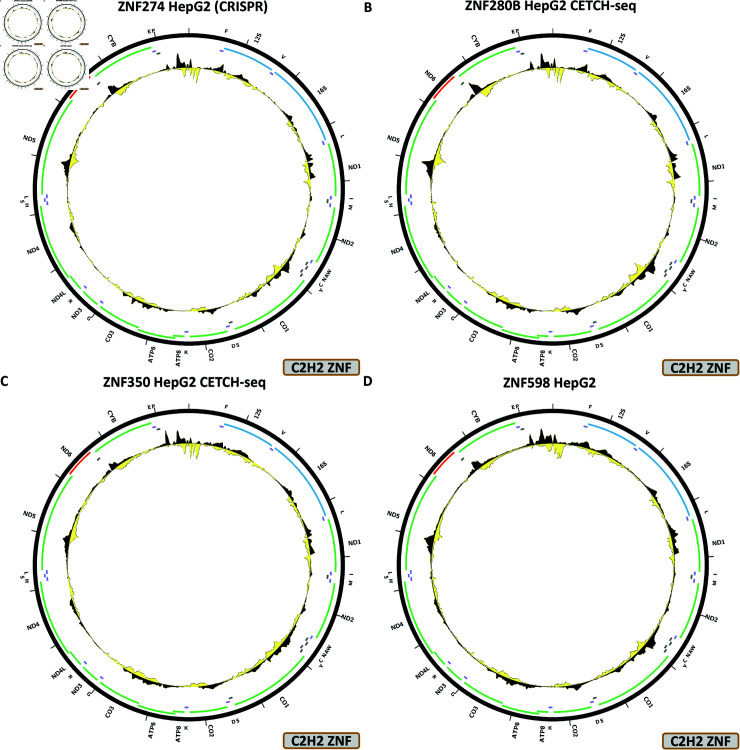
Evidence for mitochondrial genome occupancy by nuclear transcription factors. Black and yellow tracks show the forward- and reverse-strand ChIP-seq coverage over chrM. (A) ZNF274 (C2H2 ZNF); (B) ZNF280B (C2H2 ZNF); (C) ZNF350 (C2H2 ZNF); (D) ZNF598 (C2H2 ZNF).

**Fig 11:**
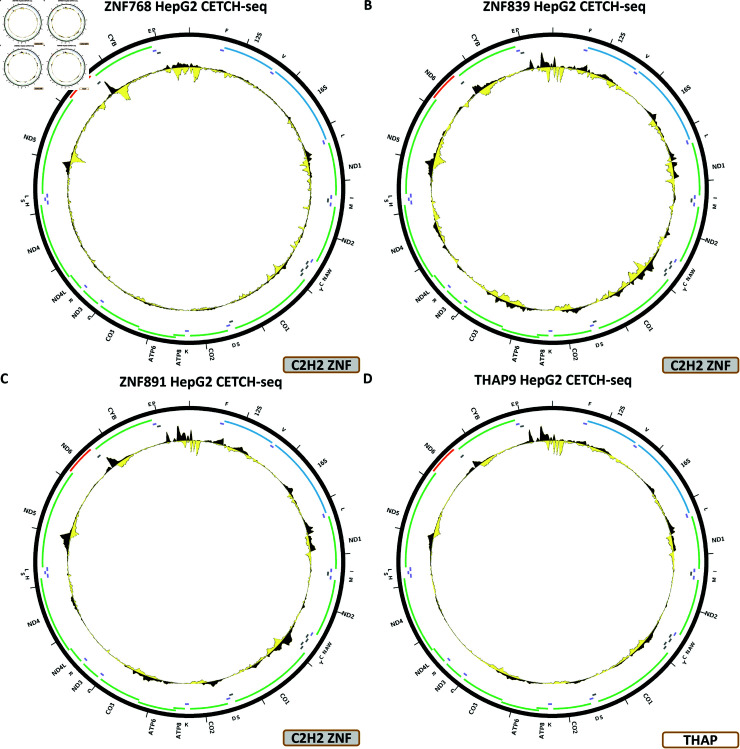
Evidence for mitochondrial genome occupancy by nuclear transcription factors. Black and yellow tracks show the forward- and reverse-strand ChIP-seq coverage over chrM. (A) ZNF768 (C2H2 ZNF); (B) ZNF839 (C2H2 ZNF); (C) ZNF891 (C2H2 ZNF); (D) THAP9 (THAP finger).

The DZIP1, HIVEP1, ZNF225, ZNF263, ZNF274, ZNF280B, ZNF350, ZNF598, ZNF768, ZNF839, ZNF891, all display a similar pattern over chrM, in, respectively, HepG2 CETCH-seq (Fig 9A), HepG2 CETCH-seq (Fig 9B), HepG2 CETCH-seq (Fig 9C), K562-ChIP-seq (Fig 9D), HepG2 CETCH-seq (Fig 10A), HepG2 CETCH-seq (Fig 10B), HepG2 CETCH-seq (Fig 10C), HepG2 ChIP-seq (Fig 10D), HepG2 CETCH-seq (Fig 11A), HepG2 CETCH-seq (Fig 11B), and HepG2 CETCH-seq Fig 11C). Nine out of eleven of these datasets are the result of endogenous epitope tagging experiments in HepG2 cells, but two are conventional ChIP-seq using TF-specific antibodies. They all display multiple peaks all over the length of the mitochondrial genome, and they are all generally matching the predicted BPNet profiles (S1 Text, S33B, S34B, S35B, S36B, S37B, S38B, S39B, S40B, S41B, S42B, and S43B Figs).

For most of these TFs, these are the only datasets available, but in the cases where additional experiments exist, the observed peaks are not replicated—K562 CETCH-seq for HIVEP1 (S1 Text, S34C Fig); HEK293 ChIP-seq (S1 Text, S35C Fig) and HepG2 CETCH-seq (S1 Text, S35D Fig) for ZNF263; HepG2, K562, H1-hESC, HeLa-S3, HCT116, HEK293, and GM12878 ChIP-seq and HEK293 CETCH-seq for ZNF274 (S1 Text, S35C–S35J Fig); HEK293 ChIP-seq for ZNF350 (S1 Text, S39C Fig); HEK293 ChIP-seq for ZNF768 (S1 Text, S41C Fig).

### THAP finger TFs

The human genome encodes 12 THAP finger TFs, for five of which datasets exist in the ENCODE collection.

Fig 11A shows the chrM CETCH-seq profile for the THAP9 TF in the HepG2 cell line. This factor displays largely the same profile as most of the C2H2 zinc finger TFs discussed above, and it too matches BPNet predictions (S1 Text, S44B Fig).

### Rel TFs

Of the ten Rel TFs in the human genome, six have been assayed by ENCODE.

**Fig 12:**
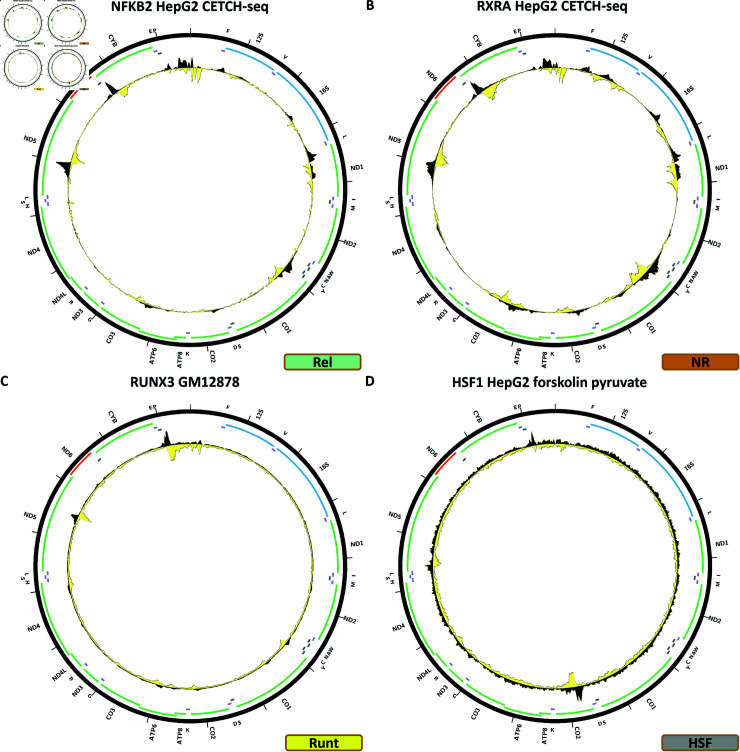
Evidence for mitochondrial genome occupancy by nuclear transcription factors. Black and yellow tracks show the forward- and reverse-strand ChIP-seq coverage over chrM. (A) NFKB2 (Rel); (B) RXRA (nuclear receptor); (C) RUNX3 (Runt); (D) HSF1 (HSF).

Fig 12A shows the chrM CETCH-seq profile for the NFKB2 TF in the HepG2 cell line. This dataset too exhibits a similar pattern as THAP9 and most of the C2H2 zinc finger TFs, matched by BPNet predictions (S1 Text, S45B Fig).

### Nuclear receptors

Of the 46 human nuclear receptors, 26 have been assayed by ENCODE.

Fig 12B shows the chrM CETCH-seq profile for the RXRA TF in the HepG2 cell line. This is another case of the same pattern observed for most of the C2H2 zinc finger TFs, THAP9 and NFKB2, and it too largely matches BPNet predictions (S1 Text, S46B Fig). This pattern is not replicated in any of the ChIP-seq datasets available for RXRA in HepG2, GM12878, H1-hESC, liver and SK-N-SH, generated using *α*-RXRA antibodies.

### Runt TFs

Three RUNT TFs are encoded in the human genome. Two of these have been assayed by ENCODE.

Fig 12C shows the chrM ChIP-seq profile for the RUNX3 TF in the GM12878 cell line. One strong peak is observed over the *MT-ND5* gene, two weaker ones over *MT-CO1*, as well as several other loci of slight enrichment. We were not able to train a high quality BPNet model for this TF (S1 Text, S47B Fig).

### HSF TFs

ENCODE has assayed three out of eight HSF TFs.

Fig 12D shows the chrM ChIP-seq profile for the HSF1 TF in HepG2 cells treated with forskolin + 1mM pyruvate. One peak is observed over the *MT-CO2* gene, but it is not corroborated by BPNet predictions (S1 Text, S48B Fig) and is not replicated in HSF1 ChIP-seq datasets in GM12878 and MCF-7 cells (Fig 48C–48D).

### Homeodomain TFs

Homeodomain transcription factors are the second largest class of TFs in mammalian genomes. The human genome encodes 202 of them, plus seven CUT Homeodomain TFs, 16 POU Homeodomain TFs, and nine Paired-box Homeodomain TFs (treated separately in the classification followed here [1]). We observe evidence for mtDNA occupancy for two Homeodomain TFs and one CUT Homeodomain TF.

**Fig 13:**
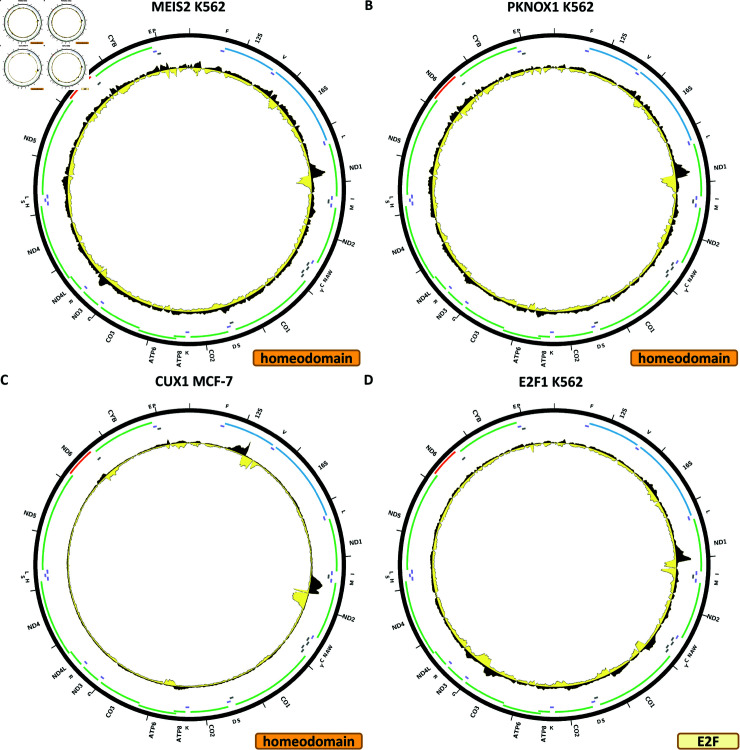
Evidence for mitochondrial genome occupancy by nuclear transcription factors. Black and yellow tracks show the forward- and reverse-strand ChIP-seq coverage over chrM. (A) MEIS2 (Homeodomain); (B) PKNOX1 (Homeodomain); (C) CUX1 (Homeodomain); (D) E2F1 (E2F).

Fig 13A shows the chrM ChIP-seq profile for the MEIS2 TF in the K562 cell line. One moderate peak is observed over the *MT-ND1* gene. BPNet predicts elevated signal over that region, but also over many other sites in the mitochondrial genome (S1 Text, S49B Fig).

Fig 13B shows the chrM ChIP-seq profile for the PKNOX1 TF in the K562 cell line. The same peak as for MEIS2 is seen, and in this case too BPNet predictions do not match well the observed profile (Fig 50B). The peak is replicated in HEK293T cells (S1 Text, S50D Fig), but not in GM12878 (S1 Text, S50C Fig) or MCF-7 (S1 Text, S50E Fig).

Fig 13C shows the chrM ChIP-seq profile for the CUX1 TF in the MCF-7 cell line. Two strong peaks are observed—over the 12S rRNA gene and over *MT-ND2*, as well as a weaker one over *MT-ATP6*. BPNet predictions include these peaks but also many others, and their relative predicted and observed strengths do not match well (S1 Text, S51B Fig). These patterns are replicated in K562 CETCH-seq experiment (S1 Text, S51D Fig), but not in K562 ChIP-seq and GM12878 experiments carried out with the same *α*-CUX1 antibody (S1 Text, S51C and S51E Fig).

### E2F TFs

The E2F family consists of 11 TFs in the human genome, for which data is available for ten.

Fig 13D shows the chrM ChIP-seq profile for the E2F1 TF in the K562 cell line. A strong peak is observed over the *MT-ND1* gene, as well as elevated signal around several other loci in the mitochondrial genome. However, this does not match the BPNet-predicted profile (S1 Text, S52B Fig), and it is also not replicated in any of the other available datasets—ChIP-seq in K562 cells generated using a different *α*-E2F1 antibody (S1 Text, S52C Fig), ChIP-seq in MCF-7 cells carried out using HA-tagged E2F1 (S1 Text, S52D Fig), ChIP-seq in HeLaS3 cells carried out using a third *α*-E2F1 antibody (S1 Text, S52E Fig), and CETCH-seq experiments in HepG2 and HeLa-S3 cells (S1 Text, S52F–S52G Fig).

### ARID/BRIGHT TFs

Eight out of 15 ARID/BRIGHT TFs in the human genome have been assayed by ENCODE.

**Fig 14:**
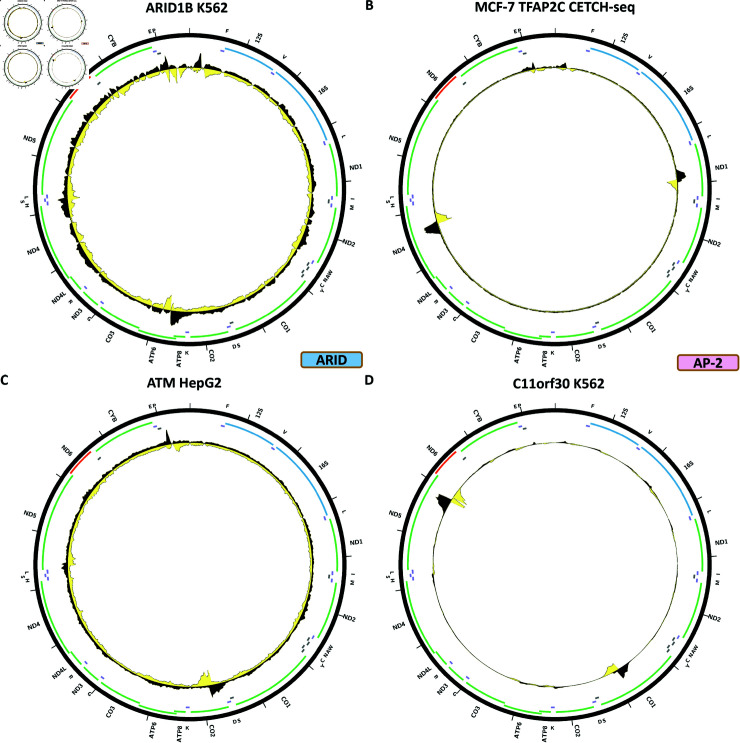
Evidence for mitochondrial genome occupancy by nuclear transcription factors. Black and yellow tracks show the forward- and reverse-strand ChIP-seq coverage over chrM. (A) ARID1B (ARID/BRIGHT) (B) TFAP2C (AP-2); (C) C11orf30; (D) ATM.

Fig 14A shows the chrM ChIP-seq profile for the ARID1B TF in the K562 cell line. A single peak is observed over the *MT-ATP6* gene, but we do not have BPNet support for it (S1 Text, S53B Fig), and there are no other datasets available for orthogonal evidence.

### AP-2 TFs

One of the five AP-2 TFs in the human genome has been assayed by ENCODE.

Fig 14B shows the chrM profile for the TFAP2C TF in a CETCH-seq experiment in the MCF-7 cell line. Two very strong peaks are observed—over the *MT-ND1* and *MT-ND4* genes. In this case we were not able to train a good BPNet model, and there are no other experiments available for TFAP2C.

### Other proteins

**Fig 15:**
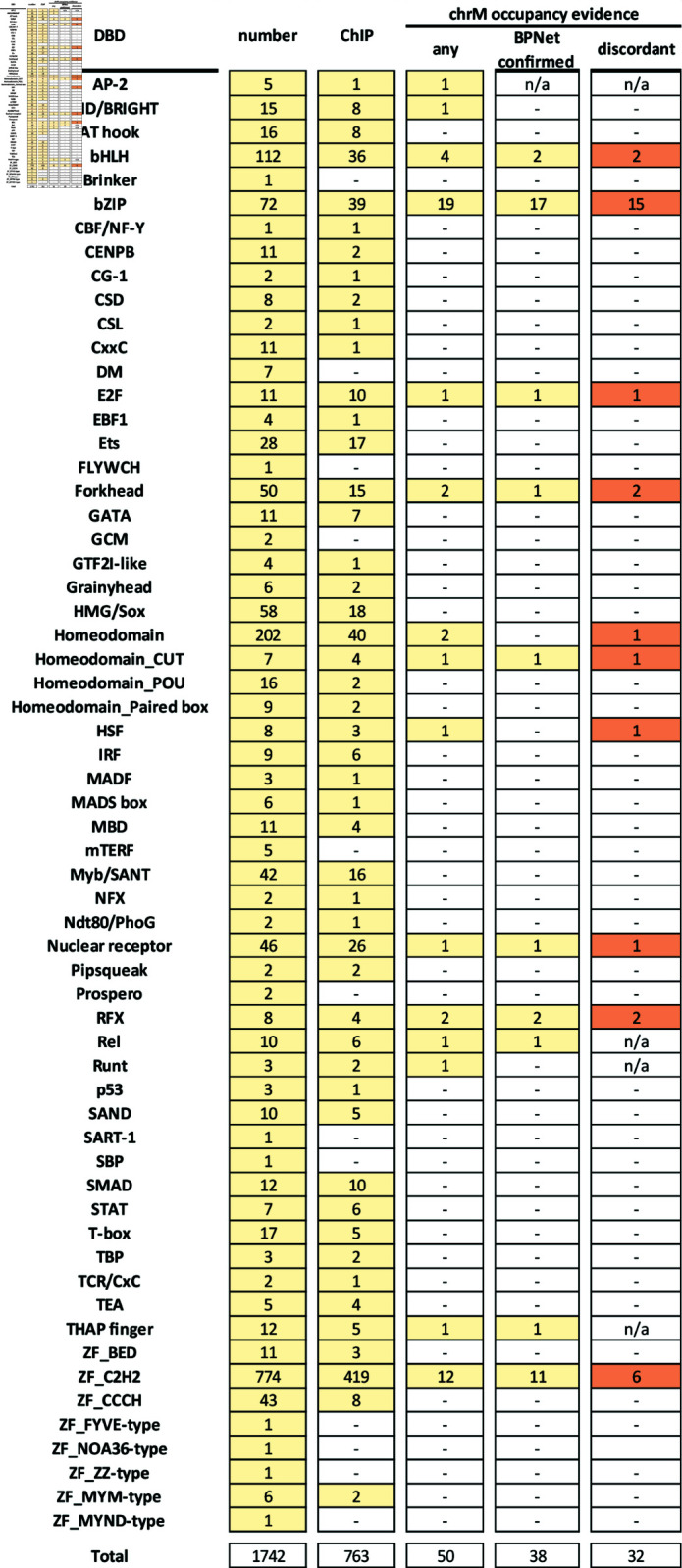
Summary of the available evidence for the physical association of nuclear TFs with the mitochondrial genome. All TFs with ChIP-seq evidence for chrM occupancy are listed in the “any” column. “BPNet confirmed” indicates that the observed ChIP-Seq pattern is corroborated in its key aspects by BPNet models. The “discordant” TFs are those for which not all available ChIP-seq experiments show chrM peaks.

We did not find any putative chrM peaks for TFs in the following families (Fig 15): AT hook (8/16 assayed), CBF/NF-Y (1/1), CENPB (2/11), CG-1 (1/2), CSD (2/8), CSL (1/2), CxxC (1/11), EBF1 (1/4), Ets (17/28), GATA (7/11), GTF2I-like (1/4), Grainyhead (2/6), HMG/Sox (18/58), IRF (6/9), MADF (1/3), MADS box (1/6), MBD (4/11), Myb/SANT (16/42), NFX (1/2), Ndt80/PhoG (1/2), Pipsqueak (2/2), p53 (1/3), SAND (5/10), SMAD (10/12), STAT (6/7), T-box (5/17), TBP (2/3), TCR/CxC (1/2), TEA (4/5), BED ZF (3/11), CCCH ZF (8/43), and MYM-type ZF (2/16).

We observed notable chrM peaks for two other chromatin proteins—C11orf30/EMSY and ATM.

Fig 14C shows the chrM ChIP-seq profile for the C11orf30/EMSY protein in the K562 cell line. Two very strong peaks are observed—over the *MT-CO1* and *MT-ND5* genes. We trained a BPNet model which predicts peaks over a large set of loci, but does include these two peaks (S1 Text, S48B Fig). No other datasets are available as orthogonal evidence.

Fig 14D shows the chrM ChIP-seq profile for the ATM TF in the HepG2 cell line. A peak is observed over the *MT-CO2* gene, but the existing BPNet model does not make high-quality predictions over chrM (S1 Text, S56B Fig)

## Discussion

In this work we review the current evidence for mtDNA occupancy by nuclear transcription factors using the vastly expanded collection of ChIP-seq datasets generated by the most recent phases of the ENCODE Project Consortium together with interpretable deep neural network modeling of TF occupancy, continuing from our previous work on the subject a decade ago [27,28]. Some evidence for physical association with mtDNA is found for 50 sequence-specific TFs and two other proteins. However, the interpretation of these observations is less straightforward than it was in the past.

The updated ENCODE collection is qualitatively distinct relative to the much smaller second-phase ENCODE set in that now many TFs have been assayed multiple times, in many different cell lines, and using different combinations of distinct antibodies and/or endogenous epitope tagging. This potentially provides stronger evidence than the more limited data previously available as it can mitigate against the several major concerns that have always existed about observed ChIP-seq peaks over chrM. These are:

1. Whether the experimental protocol used, specifically the fixation step might have involved some kind of permeabilization that allows nuclear TFs to “leak” into the mitochondrial compartment and occupy mtDNA. This should in principle be unlikely if fixation is carried out directly on intact cells, and it would also be expected to result in ChIP-seq profiles showing elevated signal over most of the cognate sequence motifs present in the mitochondrial genome (rather than the one or just a handful of peaks observed in most cases). Still, concerns about such experimental variation are alleviated of mtDNA occupancy is replicated widely across a large number of datasets generated by different productions groups and in different cell lines. On the other hand, the absence of chrM peaks in all cell lines assayed might represent true biological variation.
2. Whether non-specific binding by antibodies, especially polyclonal ones, may be the source of the observed ChIP-seq peaks, i.e. the peaks are real and mediated by some unknown protein that localizes to mtDNA, but it is not the TF that is being assayed that is occupying those sites. Observing the same peaks with multiple different antibodies or a combination of an antibody and endogenous epitope tagging greatly boosts confidence in the physical occupancy of mtDNA by a given TF.
3. Whether epitope tagging affects subcellular localization and/or expression levels. Most of the epitope tagging datasets examined here were generated by C-terminal tagging of the endogenous TF gene. This means that subcellular localization and expression levels should not be affected, but nevertheless such concerns cannot be completely dismissed.

The expanded ENCODE collection provides numerous examples of lack of complete concordance between the different available experiments for each TF. It is thus reasonable to provisionally consider TFs to be likely *in vivo* mtDNA binders if there are at least two orthogonal lines of evidence for their chrM occupancy, i.e. ChIP-seq peaks in datasets generated with at least two different antibodies or an antibody and endogenous tagging, or what is the ideal gold evidential standard—a combination of peaks observed in ChIP-seq datasets and demonstration of localization to mitochondria using immunogold electron microscopy. The latter is however not entirely possible for e.g. many of the C2H2 ZFs, for which epitope tagging was used for ChIP-seq due to the unavailability of immune reagents; in such cases aberrant localization to mitochondria as a result of the tagging cannot be entirely excluded.

With these considerations in mind, we can summarize the available evidence for mtDNA occupancy as follows.

What immediately stands out in the current data is the large number of bZIP factors for which chrM peaks are observed—nearly half (19/39) of the ones that have been assayed. This is unlikely to be an artifact as numerous lines of evidence converge onto bZIP factors playing a role in mitochondria, even though the evidence for each individual TF can be contradictory. For example, ATF2 chrM peaks are seen with multiple antibodies, but not with all or in all epitope tagging experiments; ATF7 peaks are seen in multiple cell lines, but only with one of two antibodies used; FOSL1, FOSL2 and NFE2L1 peaks are not replicated beyond a single dataset, and CEBPG peaks are observed in one epitope-tagged cell line but not in others. On the other hand, CREB1 and MAFK chrM peaks are replicated with multiple different antibodies and NFE peaks in both ChIP-seq and CETCH-seq (although not in all cell lines).

The bZIP factors also include the three TFs for which direct microscopy evidence exists for localization to mitochondria—MAFK, JUN and JUND.

They also exhibit colocalization to a few distinct sites in the mitochondrial genome, which lends mutual support to each other’s mtDNA occupancy because many bZIP factors form heterodimers in the form of the AP-1 transcription factor [81]. Thus, they would be expected to co-occupy the same sites. AP-1’s nuclear functions also happen to be generally associated with the regulation of growth and proliferation; these are processes in which mitochondria play important roles. While it is currently not clear how AP-1 might be playing a regulatory role in mitochondria mechanistically, this is an obvious functional connection to consider.

On the other extreme of reliability of the available evidence lies the set of C2H2 ZF TFs (DZIP1, HIVEP1, ZNF225, ZNF263, ZNF274, ZNF280B, ZNF350, ZNF598, ZNF768, ZNF839, ZNF891) together with THAP9, NFKB2 and RXRA. All of these datasets exhibit almost the same ChIP-seq profile, are almost all derived from epitope tagging experiments, and they also show elevated signal over nearly all predicted occupancy sites. These peaks are not replicated by any antibody ChIP-seq datasets where available, and thus they are most likely an artifact, although it is not clear why only these CETCH-seq experiments would generate such an artifact and not the hundreds of others.

Evidence is also currently weak for mtDNA occupancy by BHLHE40, MITF, FOXA1, FOXA2, HSF1, E2F1, due to lack of replication and/or lack of support from BPNet predictions.

For factors such as MAX, RFX1, RFX5 and PKNOX1 ChIP-seq peaks are seen in multiple cell lines, although not in others. They are provisionally more likely to be truly occupying mtDNA than not.

Yet other factors—SREBF1, RFX1, RUNX3, CUX1, ARID1B, TFAP2C, C11orf30/EMSY and ATM—have only been assayed in a single cell line, and thus the available evidence is simply too limited to say much more about them.

Nevertheless, some broad trends emerge. While the bZIP factors appear to be particularly enriched for potential mitochondrial moonlighting, other large and important TF families show very little such evidence. Even if the 12 C2H2 ZFs turn out not to be the result of an experimental artifact, they would represent only a small fraction (12/419) of the huge diversity of such TFs. Other large TF families that, although not exhaustively sampled, don’t seem to bind to chrM include HMG/Sox, nuclear receptors, Homeodomain TFs, and Myb/SANT. A few of the smaller TF families have also been almost exhaustively sampled and they too show no evidence for mitochondrial localization. These include GATA, IRF, SMAD, STAT, and TEA.

Remaining unresolved in the key question regarding the biological role, if any, of these TFs. It is of course entirely possible, and in fact in our opinion quite likely that there is none, i.e. TFs just happen to be transported to organelles due to a confluence of molecular properties they possess, where they proceed to bind to the genome via their intrinsic sequence preference, but this has little to no functional consequences. The fact that they are all found away from the D-loop regulatory region would support such a view. Nevertheless, it is also very much possible that they do play some functional role, and in some ways our results have increased the likelihood that this is the case, as the presence of so many bZIP TFs in mitochondria, i.e. factors that generally co-bind together and often play a role in regulating growth and metabolism in the nucleus, would be consistent with some function in the mitochondrial domain.

What is needed are direct experimental tests of the hypothesis that nuclear TFs have a function in mitochondria. Unfortunately, this is where we run into serious experimental and epistemological challenges, because of the nature of the mitochondrial genome and its relationship to the nucleus. One naively obvious way to test nuclear TF function in mitochondria would be to knock out TFs and observe the effects on gene expression in mitochondria. Many such datasets in fact are readily available already. However, any such experiments are completely uninterpretable. If they show changes in chrM gene expression, there is no way to deconvolve any effects on mitochondrial transcription from the effects of the TF knockout on gene expression in the nucleus. Worse, bZIP TFs very much do regulate a lot of nuclear genes that affect the state of mitochondria. On the other hand, if no effects are seen, that is in no way evidence for the absence of a functional role in mitochondria, especially for bZIP TFs. This is because so many of them bind the same sites, i.e. there is huge redundancy, and perturbing just one TF likely has no effect.

The more refined approach is to carry out perturbations directly and specifically in the mitochondria, e.g. by disrupting the binding sites for the nuclear TFs. However, this is both very difficult technically and also plagued by confounding issues. First, the mitochondrial genome is very densely packed, and most of the observed sites are inside genes. The only type of binding site perturbation that would not be completely confounded by the disruptive effects it would have on those genes is precise base editing of non-synonymous positions in codons, and it would have to be very precisely executed with minimal editing of any nearby sites. Second, direct manipulation of the mitochondrial genome has been traditionally very difficult due to challenge of delivery of genome editing agents to the inner mitochondrial compartment. In recent years, great strides have been made towards resolving that problem, e.g. through the use of deaminases fused to TALE sequence targeting proteins [82–88], and these could in principle be used to engineer mutations in at least some cases in which a single base happens to be both located in a degenerate codon position and is predicted to completely disrupt TF occupancy. However, what is also required in the case of dissecting the functional effects of nuclear TF binding sites is high penetrance of the induced edits, i.e. most of the potentially many thousands of mitochondrial genome copies in cells have to be edited the same way, as in this case the goal is not to generate mutations and then select for them, as is the case with genome editing, but to examine the phenotypic effects of these mutations. For all these reasons, we are still quite some way from being able to conclusively answer the question of whether nuclear TFs moonlight in mitochondria as mere aimless wanderers or they actually carry out an important function.

## Conclusion

In summary, our work represents the most comprehensive catalog of human TFs potentially occupying mitochondrial DNA compiled so far, and provides the foundation for the subsequent direct validation and characterization of the possible functions of these factors in mitochondrial gene regulation.

## Supporting information

S1 Text Contains the Supplementary Figures. (PDF)

## Acknowledgments

The authors would like to thank members of the Kundaje and Greenleaf labs for useful comments and discussions.
