# Supplementary material for: An updated compendium and reevaluation of the evidence for nuclear transcription factor occupancy over the mitochondrial genome
Source: PLoS One. 2025 Mar 31;20(3):e0318796. doi: 10.1371/journal.pone.0318796 (PMC11957562; doi:10.1371/journal.pone.0318796)
Supplement: S1 Text — (PDF) [file pone.0318796.s001.pdf]

Supplementary Materials

Supplementary Figures

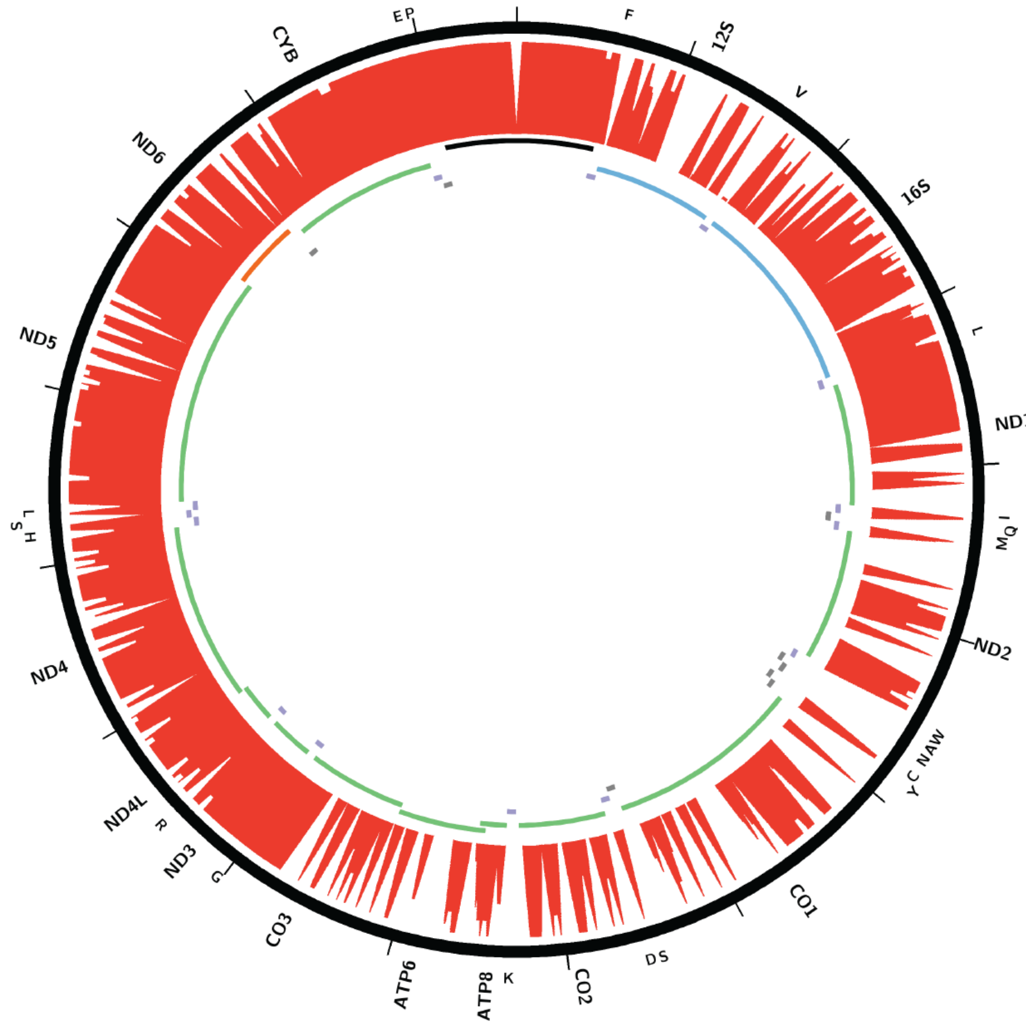

Supplementary Figure 1: Unique mappability (for 1×36mer reads) of the mitochondrial genome in the combined nuclear plus mitochondrial genomic space.

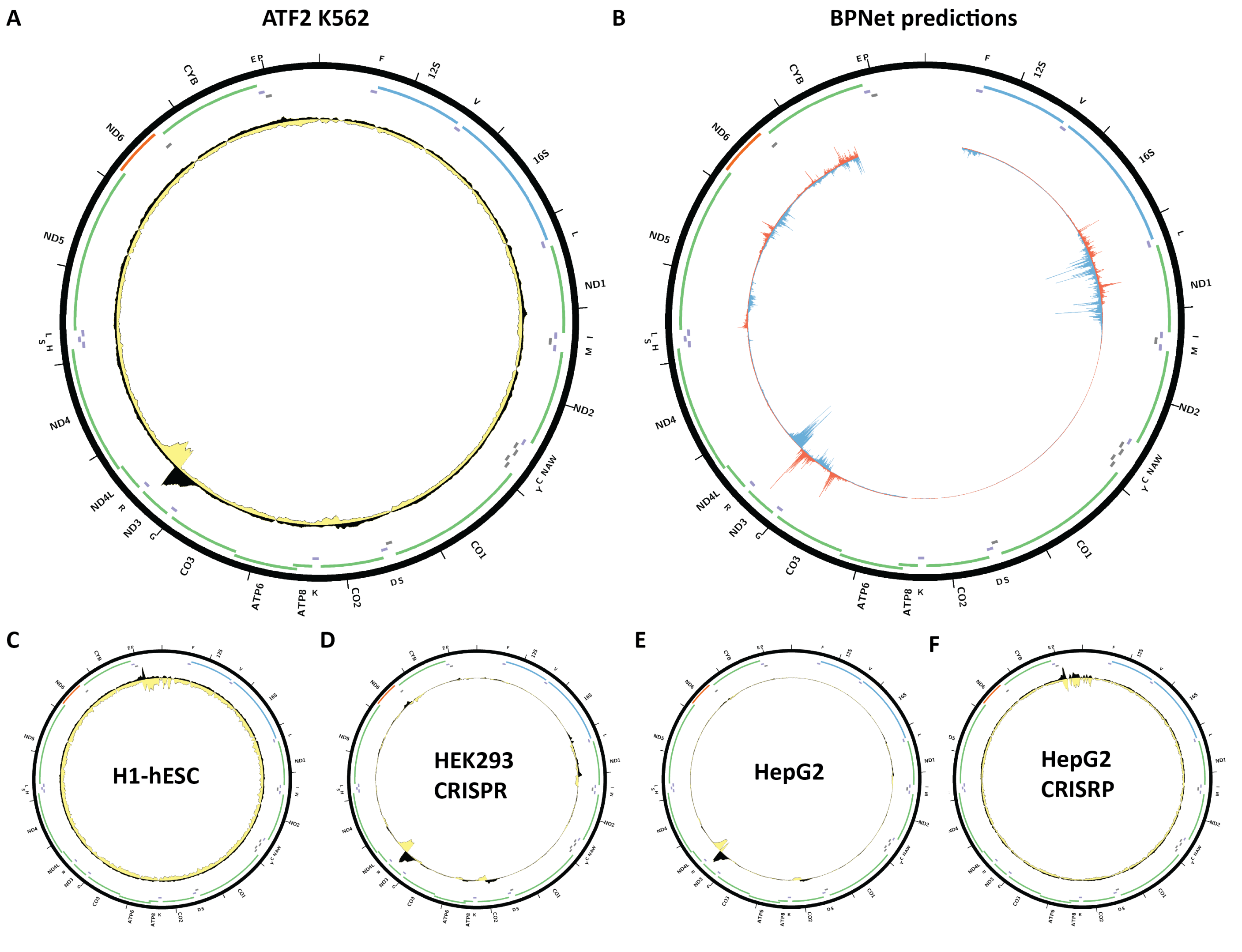

Supplementary Figure 2: Evidence for mitochondrial genome occupancy by the ATF2 transcription factor. Black and yellow tracks show the forward- and reverse-strand ChIP-seq coverage over chrM. (A) K562 ChIP-seq (ENCODE ID ENCSR869IUD; antibody Bethyl Labs A301-649A). (B) BPNet predictions over chrM (ENCODE ID ENCSR762MDC); (C) H1-hESC ChIP-seq (ENCODE ID ENCSR000BQU; antibody Santa Cruz Biotech sc-81188, Lot ID H0609); (D) HEK293 CETCH-seq (ENCODE ID ENCSR217HTK). (E) HepG2 ChIP-seq (ENCODE ID ENCSR047BUZ; antibody: Bethyl Labs A301-649A); (F) HepG2 CETCH-seq (ENCODE ID ENCSR908HWZ);

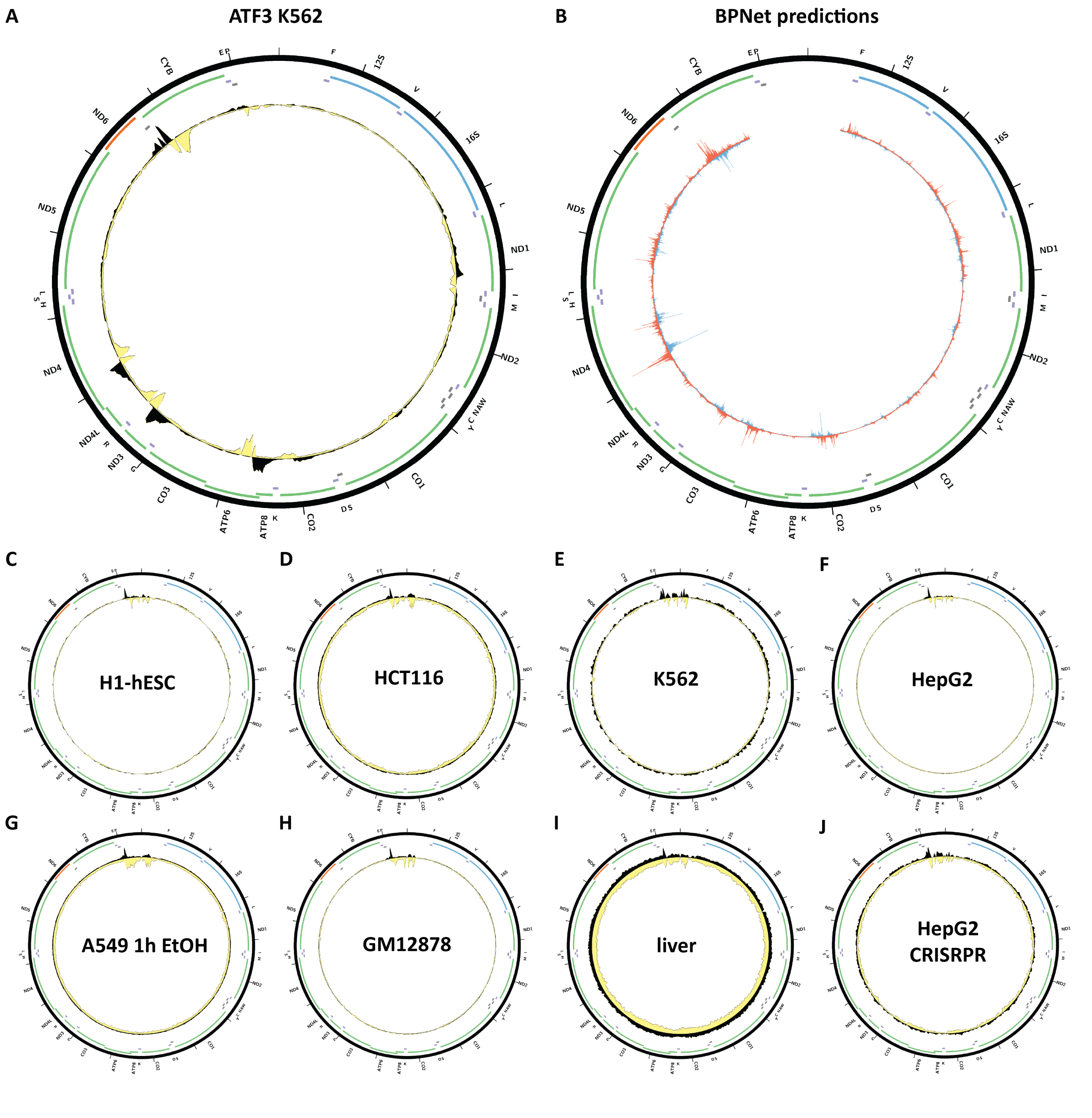

Supplementary Figure 3: Evidence for mitochondrial genome occupancy by the ATF3 transcription factor. Black and yellow tracks show the forward- and reverse-strand ChIP-seq coverage over the mitochondrial genome. (A) K562 ChIP-seq (ENCODE ID ENCSR028UIU; antibody: Active Motif 2/61715); (B) BPNet predictions over chrM (ENCODE ID ENCSR405ECI); (C) H1-hESC ChIP-seq (ENCODE ID ENCSR000BKC; antibody: Santa Cruz Biotech sc-188, Lot ID J2209); (D) HCT116 ChIP-seq (ENCODE ID ENCSR000BUG; antibody: Santa Cruz Biotech sc-188, Lot ID J2209); (E) K562 ChIP-seq (ENCODE ID ENCSR000BNU; antibody: Santa Cruz Biotech sc-188, Lot ID J2209); (F) HepG2 ChIP-seq (ENCODE ID ENCSR000BKE; antibody: Santa Cruz Biotech sc-188, Lot ID J2209); (G) A549 0.02% EtOH 1h ChIP-seq (ENCODE ID ENCSR000BPS; antibody: Santa Cruz Biotech sc-188, Lot ID J2209); (H) GM18278 ChIP-seq (ENCODE ID ENCSR000BJY; antibody: Santa Cruz Biotech sc-188, Lot ID J2209); (I) liver ChIP-seq (ENCODE ID ENCSR480LIS; antibody: Santa Cruz Biotech sc-188, Lot ID J2209); (J) HepG2 CETCH-seq (ENCODE ID ENCSR402ZCY).

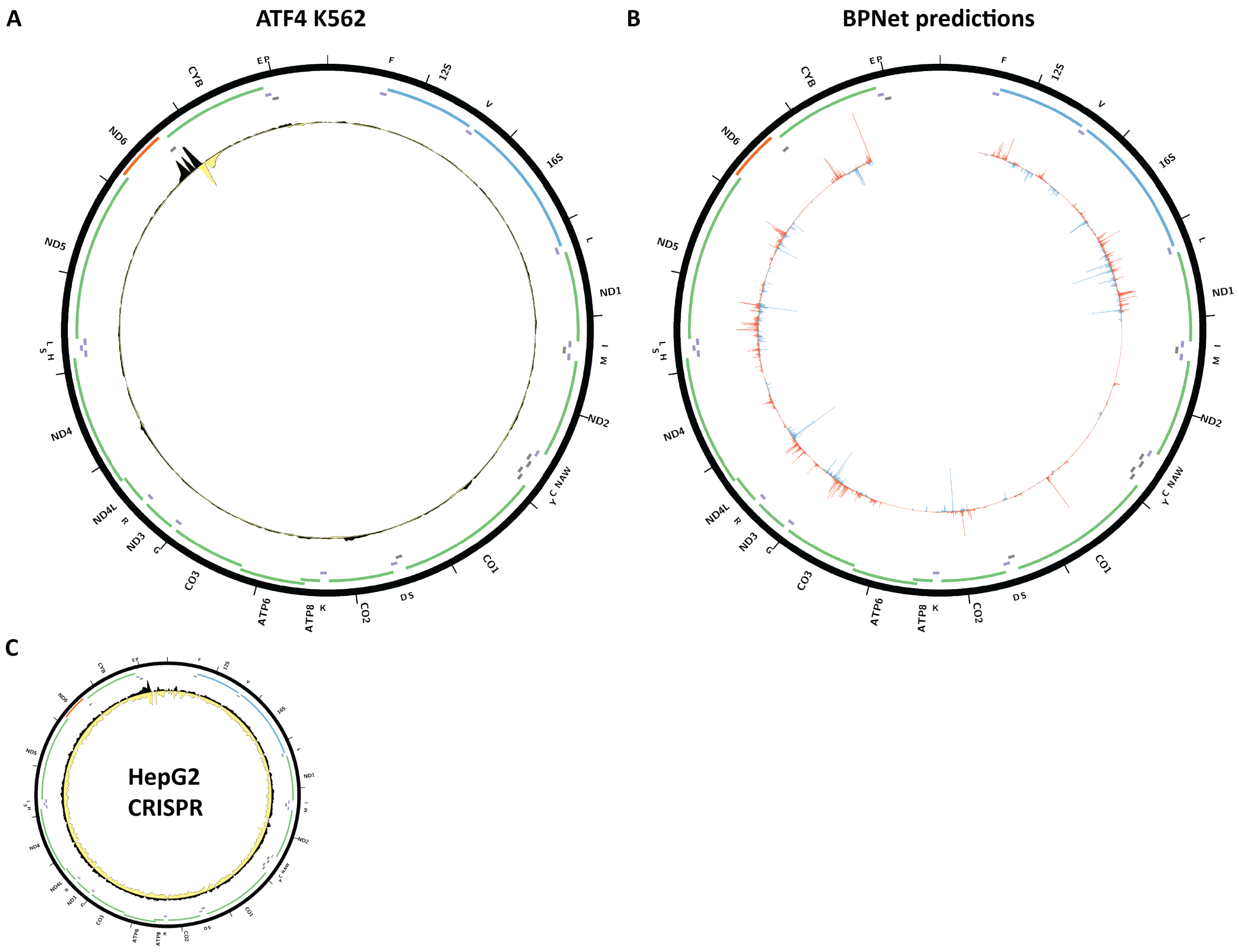

Supplementary Figure 4: Evidence for mitochondrial genome occupancy by the ATF4 transcription factor. Black and yellow tracks show the forward- and reverse-strand ChIP-seq coverage over chrM. (A) K562 ChIP-seq (ENCODE ID ENCSR145TSJ; antibody: Cell Signaling 11815S); (B) BPNet predictions over chrM (ENCODE ID ENCSR254DFG); (C) HepG2 CETCH-seq (ENCODE ID ENCSR288ZFV).

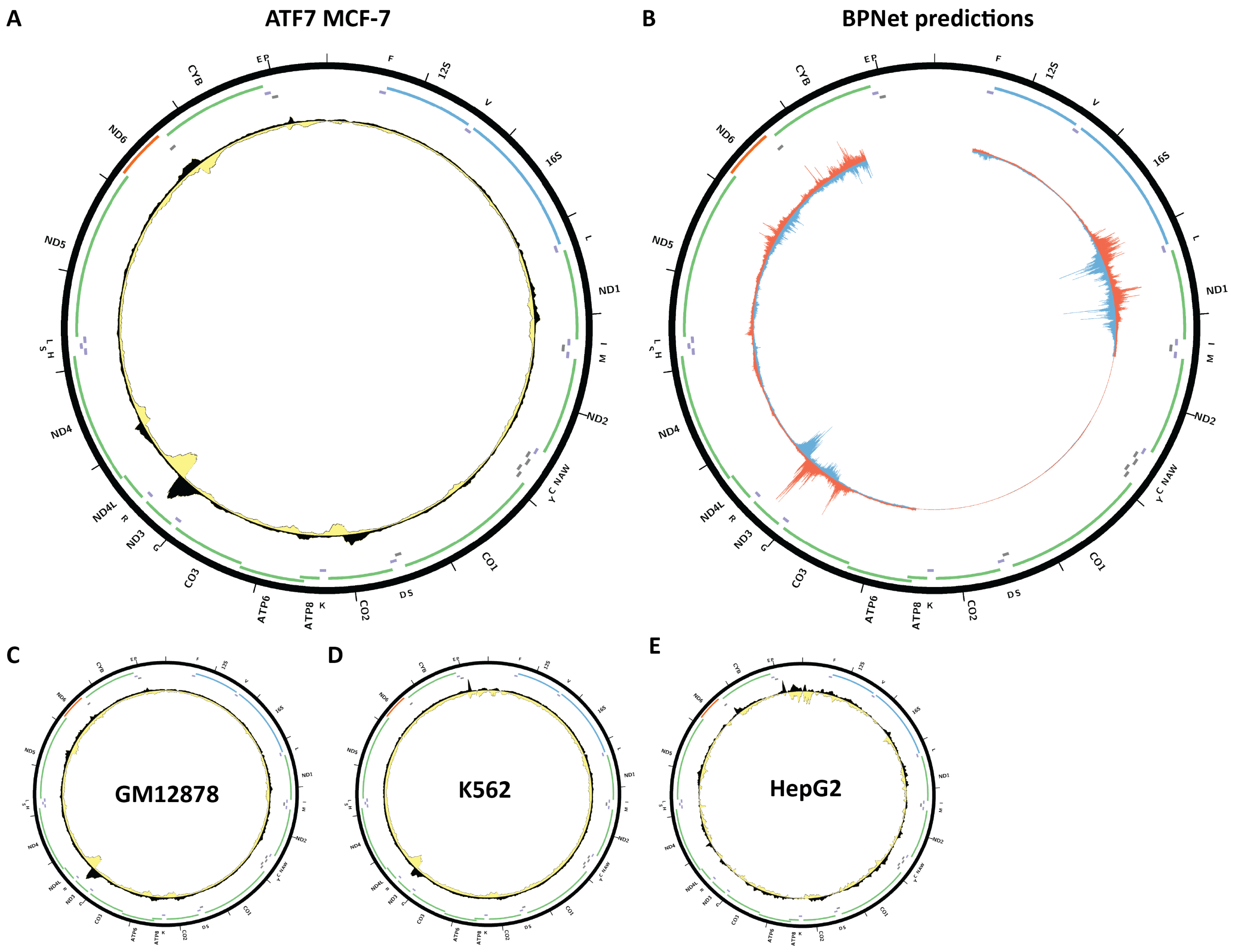

Supplementary Figure 5: Evidence for mitochondrial genome occupancy by the ATF7 transcription factor. Black and yellow tracks show the forward- and reverse-strand ChIP-seq coverage over chrM. (A) MCF-7 ChIP-Seq (ENCODE ID ENCSR866QPZ; antibody Sigma HPA003384, Lot ID R04563); (B) BPNet predictions over chrM; (C) GM12878 ChIP-seq (ENCODE ID ENCSR014YCR; antibody Sigma HPA003384, Lot ID R04563); (D) K562 ChIP-seq (ENCODE ID ENCSR972ZBV; antibody Sigma HPA003384, Lot ID R04563); (E) HepG2 ChIP-seq (ENCODE ID ENCSR516DDO; antibody: Sigma F1804, Lot ID SLBK1346V).

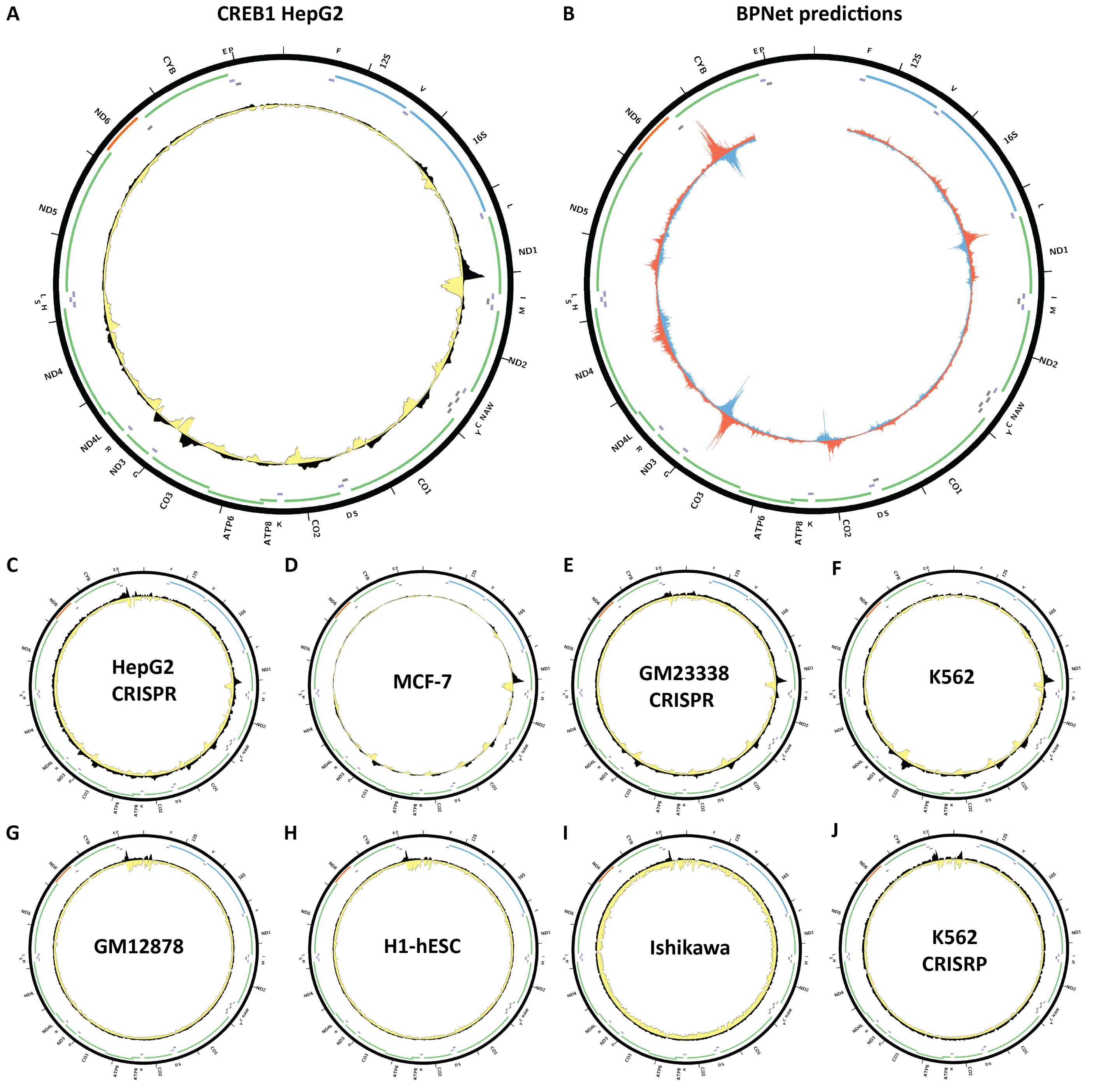

Supplementary Figure 6: Evidence for mitochondrial genome occupancy by the CREB1 transcription factor. Black and yellow tracks show the forward- and reverse-strand ChIP-seq coverage over chrM. (A) HepG2 ChIP-seq (ENCODE ID ENCSR112ALD; antibody: Cell Signaling 9197S, Lot ID 16); (B) BPNet predictions over chrM (ENCODE ID ENCSR590FMJ); (C) HepG2 CETCH-seq (ENCODE ID ENCSR027JQN); (D) MCF-7 ChIP-seq (ENCODE ID ENCSR897JAS; antibody: Cell Signaling 9197S, Lot ID 16); (E) GM23338 CETCH-seq (ENCODE ID ENCSR214ZAV); (F) K562 ChIP-seq (ENCODE ID ENCSR000BSO; antibody: Santa Cruz Biotech sc-240, Lot ID C2306); (G) GM12878 ChIP-seq (ENCODE ID ENCSR000BUF; antibody: Santa Cruz Biotech sc-240, Lot ID C2306); (H) H1-hESC ChIP-seq (ENCODE ID ENCSR000BSN; antibody: Santa Cruz Biotech sc-240, Lot ID C2306); (I) Ishikawa ChIP-seq (ENCODE ID ENCSR000BUR; antibody: Santa Cruz Biotech sc-240, Lot ID C2306); (F) K562 CETCH-seq (ENCODE ID ENCSR016RFR).

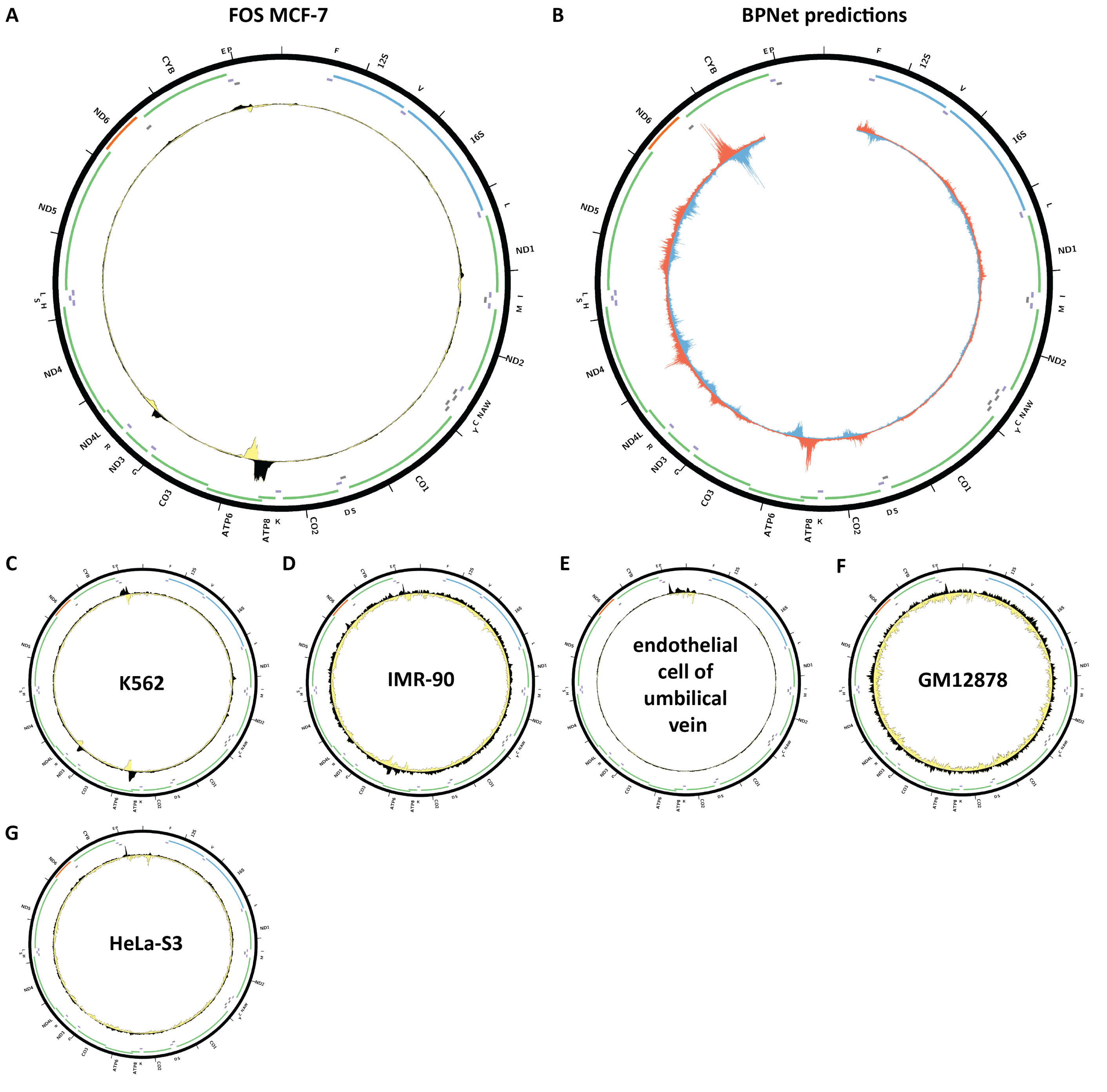

Supplementary Figure 7: Evidence for mitochondrial genome occupancy by the FOS transcription factor. Black and yellow tracks show the forward- and reverse-strand ChIP-seq coverage over chrM. (A) MCF-7 ChIP-seq (ENCODE ID ENCSR569XNP; antibody: Santa Cruz Biotech sc-7202, Lot ID K0810); (B) BPNet predictions over chrM (ENCODE ID ENCSR335UTI); (C) K562 ChIP-seq (ENCODE ID ENCSR000FAI; antibody: Santa Cruz Biotech sc-7202, Lot ID K0810); (D) IMR-90 ChIP-seq (ENCODE ID ENCSR124AIG; antibody: Santa Cruz Biotech sc-7202, Lot ID K0810); (E) endothelial cell of umbilical vein ChIP-seq (ENCODE ID ENCSR000EVU; antibody: Santa Cruz Biotech sc-7202, Lot ID K0810); (F) GM12878 ChIP-seq (ENCODE ID ENCSR000EYZ; antibody: Santa Cruz Biotech sc-7202, Lot ID K0810); (G) HeLa-S3 ChIP-seq (ENCODE ID ENCSR000EZE; antibody: Santa Cruz Biotech sc-7202, Lot ID K0810).

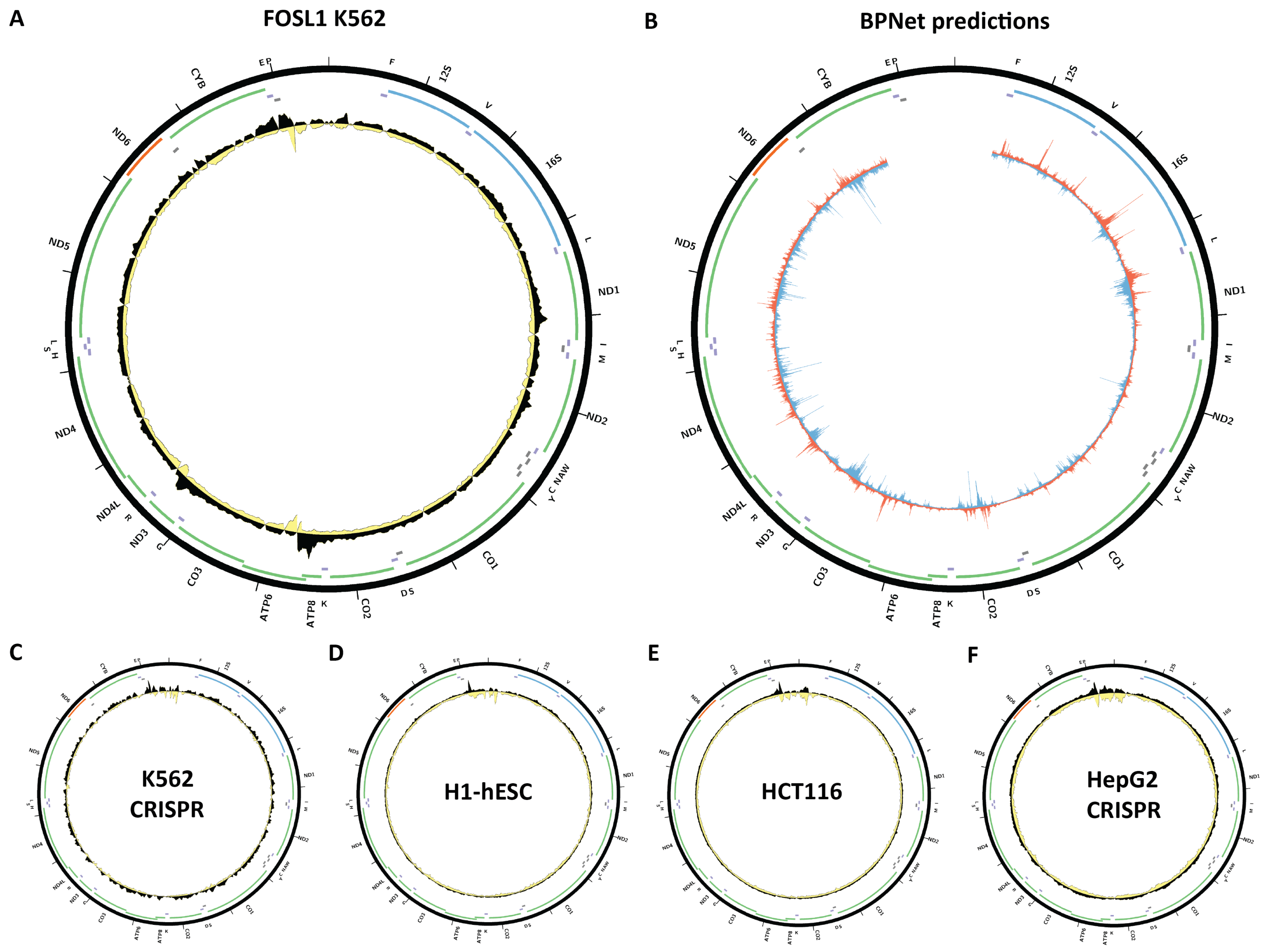

Supplementary Figure 8: Evidence for mitochondrial genome occupancy by the FOSL1 transcription factor. Black and yellow tracks show the forward- and reverse-strand ChIP-seq coverage over chrM. (A) K562 ChIP-seq (ENCODE ID ENCSR239ZLZ; GFP-tagged); (B) BPNet predictions over chrM (ENCODE ID ENCSR285NIO); (C) K562 CETCH-seq (ENCODE ID ENCSR000BMV); (D) H1-hESC ChIP-seq (ENCODE ID ENCSR000BNS; antibody: Santa Cruz Biotech sc-183, Lot ID I0809); (E) HCT116 ChIP-seq (ENCODE ID ENCSR000BTE; antibody: Santa Cruz Biotech sc-183, Lot ID I0809); (F) HepG2 CETCH-seq (ENCODE ID ENCFF660RIA).

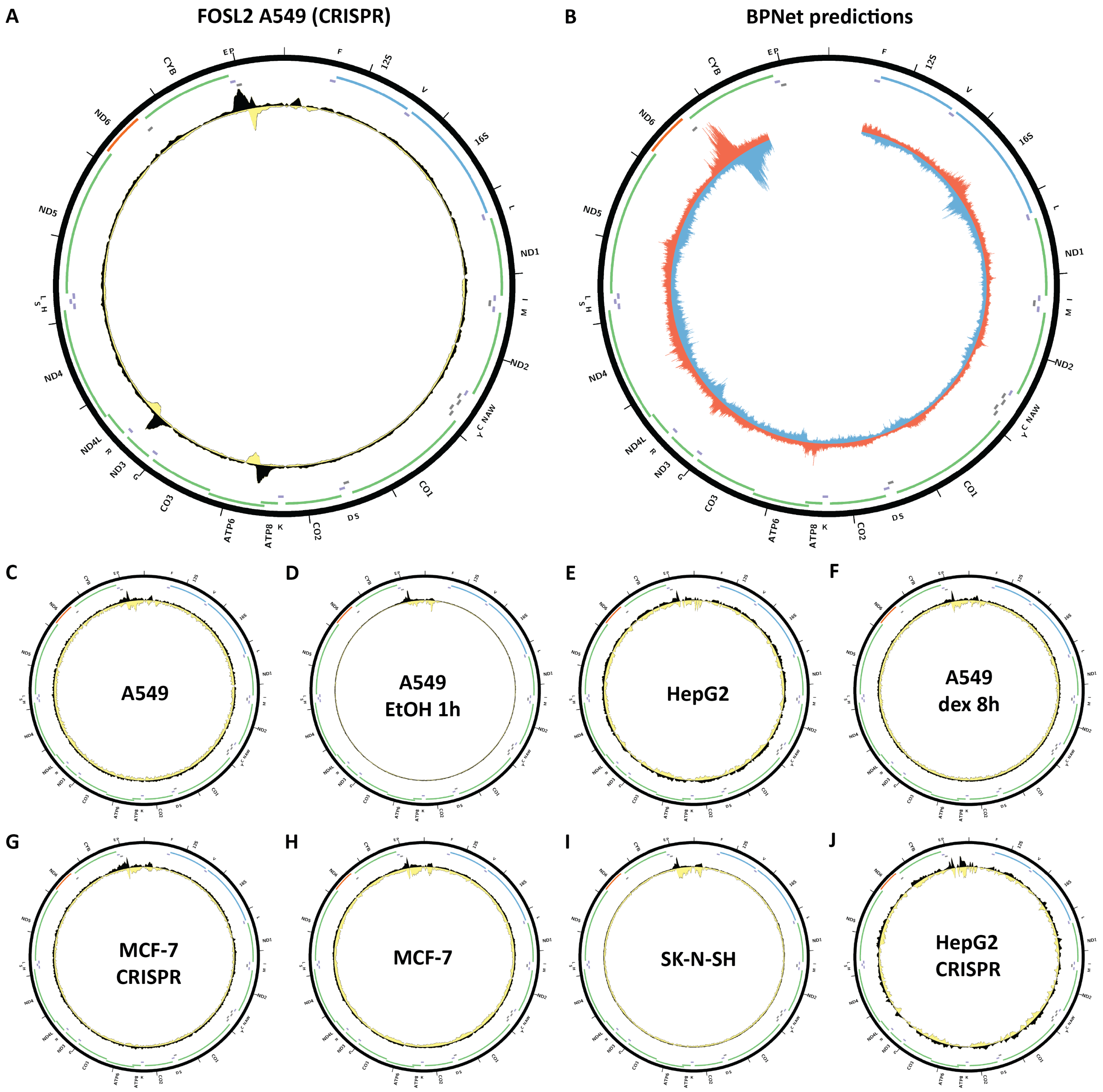

Supplementary Figure 9: Evidence for mitochondrial genome occupancy by the FOSL2 transcription factor. Black and yellow tracks show the forward- and reverse-strand ChIP-seq coverage over chrM. (A) A549 CETCH-seq (ENCODE ID ENCSR448TVS); (B) BPNet predictions over chrM (ENCODE ID ENCSR554ZTS); (C) A549 ChIP-seq (ENCODE ID ENCSR593DGU; antibody: Santa Cruz Biotech sc-160, Lot ID H2713); (D) A549 EtOH 1 hour ChIP-seq (ENCODE ID ENCSR000BQO; antibody: Santa Cruz Biotech sc-604); (E) HepG2 ChIP-seq (ENCODE ID ENCSR000BHP; antibody: Santa Cruz Biotech sc-604); (F) A549 Dex 8 hours ChIP-seq (ENCODE ID ENCSR242EWU; antibody: Santa Cruz Biotech sc-160, Lot ID H2713); (G) MCF-7 CETCH-seq (ENCODE ID ENCSR546KCN); (H) MCF-7 ChIP-seq (ENCODE ID ENCSR000BUI; antibody: Santa Cruz Biotech sc-604); (I) SK-N-SH ChIP-seq (ENCODE ID ENCSR000BVB; antibody: Santa Cruz Biotech sc-604); (J) HepG2 CETCH-seq (ENCODE ID ENCSR249EYB).

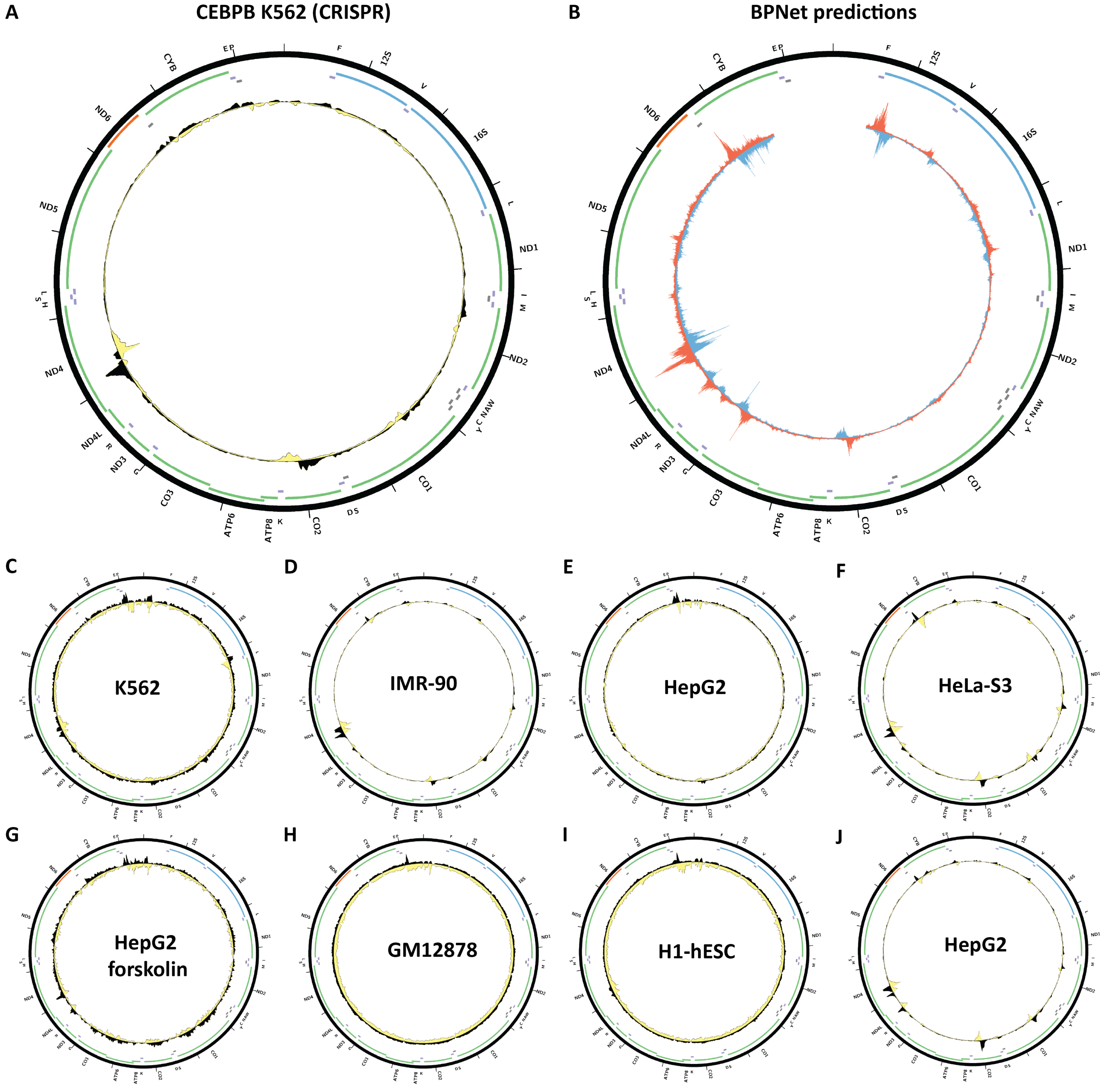

Supplementary Figure 10: Evidence for mitochondrial genome occupancy by the CEBPB transcription factor. Black and yellow tracks show the forward- and reverse-strand ChIP-seq coverage over chrM. (A) K562 CETCH-seq (ENCODE ID ENCSR416QLJ); (B) BPNet predictions over chrM (ENCODE ID ENCSR700UVP); (C) K562 ChIP-seq (ENCODE ID ENCSR000EHE; antibody: Santa Cruz Biotech sc-150, Lot ID I1010); (D) IMR-90 ChIP-seq (ENCODE ID ENCSR000EFM; antibody: Santa Cruz Biotech sc-150, Lot ID I1010); (E) HepG2 ChIP-seq (ENCODE ID ENCSR000EEE; antibody: Santa Cruz Biotech sc-150, Lot ID I1010); (F) HeLa-S3 ChIP-seq (ENCODE ID ENCSR000EDA; antibody: Santa Cruz Biotech sc-150, Lot ID I1010); (G) HepG2 forskolin ChIP-seq (ENCODE ID ENCSR000EEX; antibody: Santa Cruz Biotech sc-150, Lot ID I1010); (H) GM12878 ChIP-seq (ENCODE ID ENCSR000BRX; antibody: Santa Cruz Biotech sc-150, Lot ID I1010); (I) H1-hESC ChIP-seq (ENCODE ID ENCSR000EBV; antibody: Santa Cruz Biotech sc-150, Lot ID I1010); (J) HepG2 ChIP-seq (ENCODE ID ENCSR000BQI; antibody: Santa Cruz Biotech sc-150, Lot ID I1010).

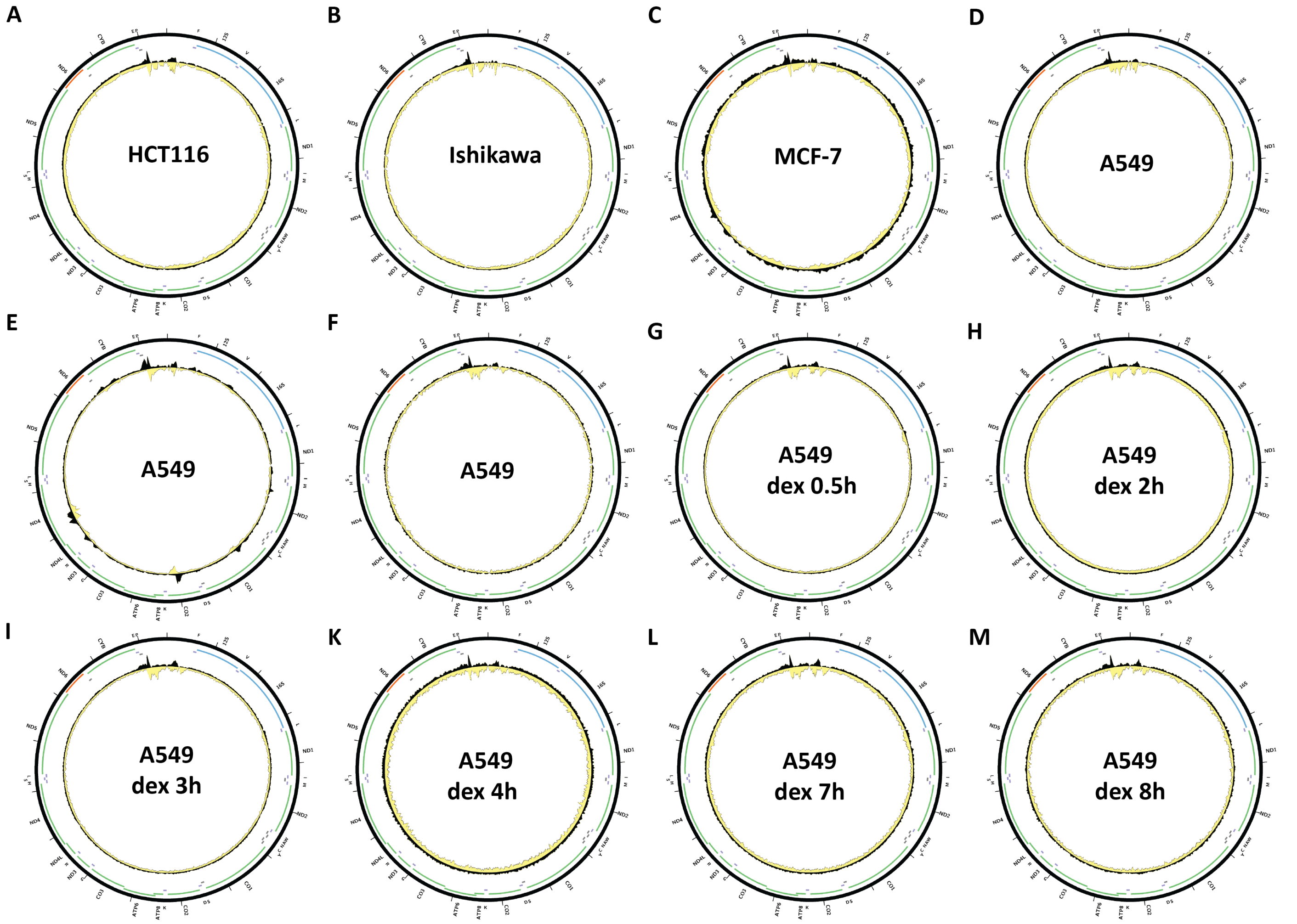

Supplementary Figure 11: Evidence for mitochondrial genome occupancy by the CEBPB transcription factor. Black and yellow tracks show the forward- and reverse-strand ChIP-seq coverage over chrM. (A) HCT116 ChIP-seq (ENCODE ID ENCSR000BSD; antibody: Santa Cruz Biotech sc-150, Lot ID I1010); (B) Ishikawa ChIP-seq (ENCODE ID ENCSR000BTT; antibody: Santa Cruz Biotech sc-150, Lot ID I1010); (C) MCF-7 ChIP-seq (ENCODE ID ENCSR000BSR; antibody: Santa Cruz Biotech sc-150, Lot ID I1010); (D) A549 ChIP-seq (ENCODE ID ENCSR000BUB; antibody: Santa Cruz Biotech sc-150, Lot ID I1010); (E) A549 ChIP-seq (ENCODE ID ENCSR000DYI; antibody: Santa Cruz Biotech sc-150, Lot ID I1010); (F) A549 ChIP-seq (ENCODE ID ENCSR701TCU; antibody: Santa Cruz Biotech sc-150, Lot ID I1010); (G) A549 dexamethasone 0.5 hours ChIP-seq (ENCODE ID ENCSR447ZMS; antibody: Santa Cruz Biotech sc-150, Lot ID D2315); (H) A549 dexamethasone 2 hours ChIP-seq (ENCODE ID ENCSR182OZC; antibody: Santa Cruz Biotech sc-150, Lot ID D2315); (I) A549 dexamethasone 3 hours ChIP-seq (ENCODE ID ENCSR216GEB; antibody: Santa Cruz Biotech sc-150, Lot ID D2315); (K) A549 dexamethasone 4 hours ChIP-seq (ENCODE ID ENCSR606ZTC; antibody: Santa Cruz Biotech sc-150, Lot ID D2315); (L) A549 dexamethasone 7 hours ChIP-seq (ENCODE ID ENCFF887KKK; antibody: Santa Cruz Biotech sc-150, Lot ID D2315); (M) A549 dexamethasone 8 hours ChIP-seq (ENCODE ID ENCSR474DCX4; antibody: Santa Cruz Biotech sc-150, Lot ID D2315).

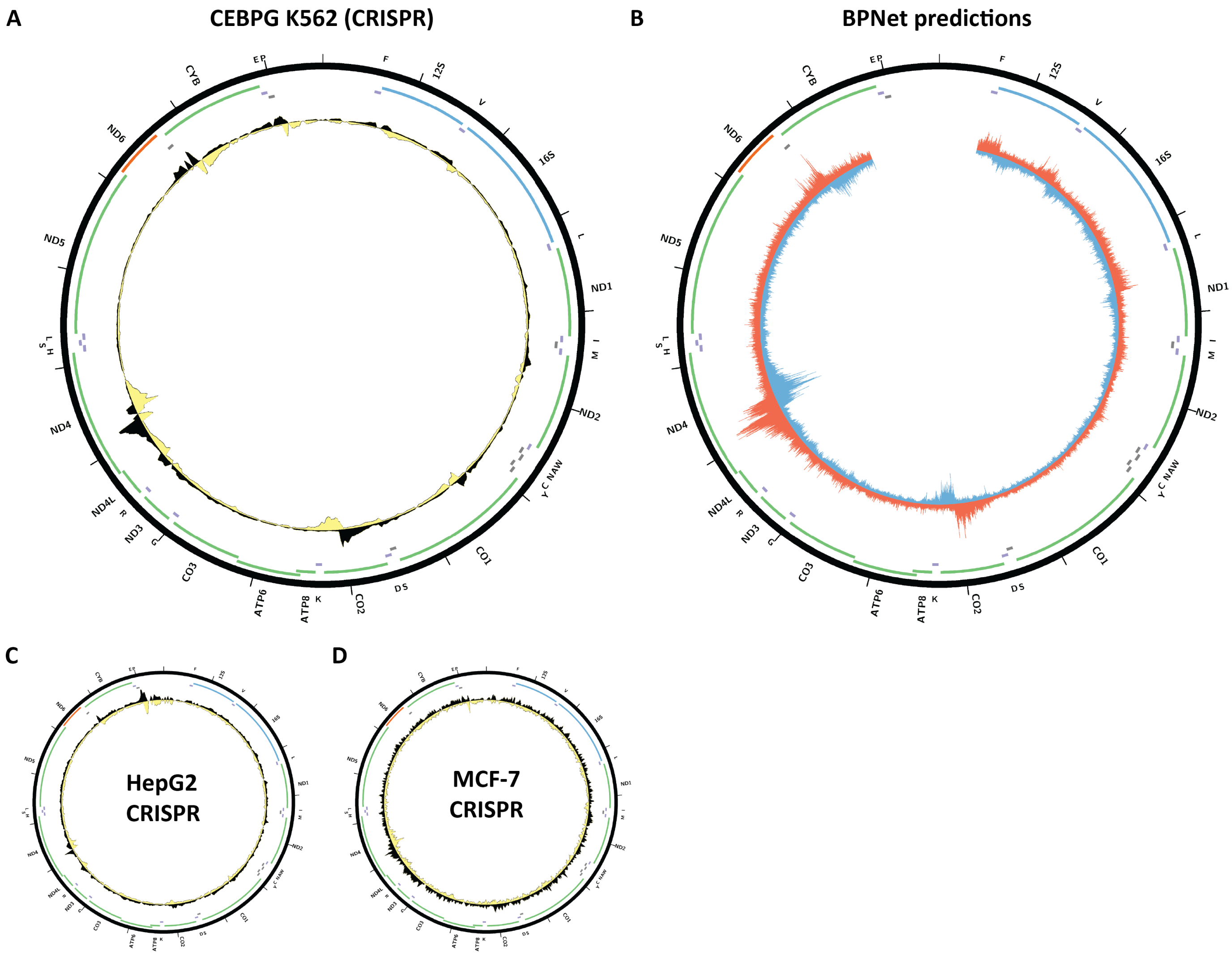

Supplementary Figure 12: Evidence for mitochondrial genome occupancy by the CEBPG transcription factor. Black and yellow tracks show the forward- and reverse-strand ChIP-seq coverage over chrM. (A) K562 CETCH-seq (ENCODE ID ENCSR620VIC); (B) BPNet predictions over chrM (ENCODE ID ENCSR136AYM); (C) HepG2 ChIP-seq (ENCODE ID ENCSR639IIZ); (D) MCF-7 ChIP-seq (ENCODE ID ENCSR094ZCF).

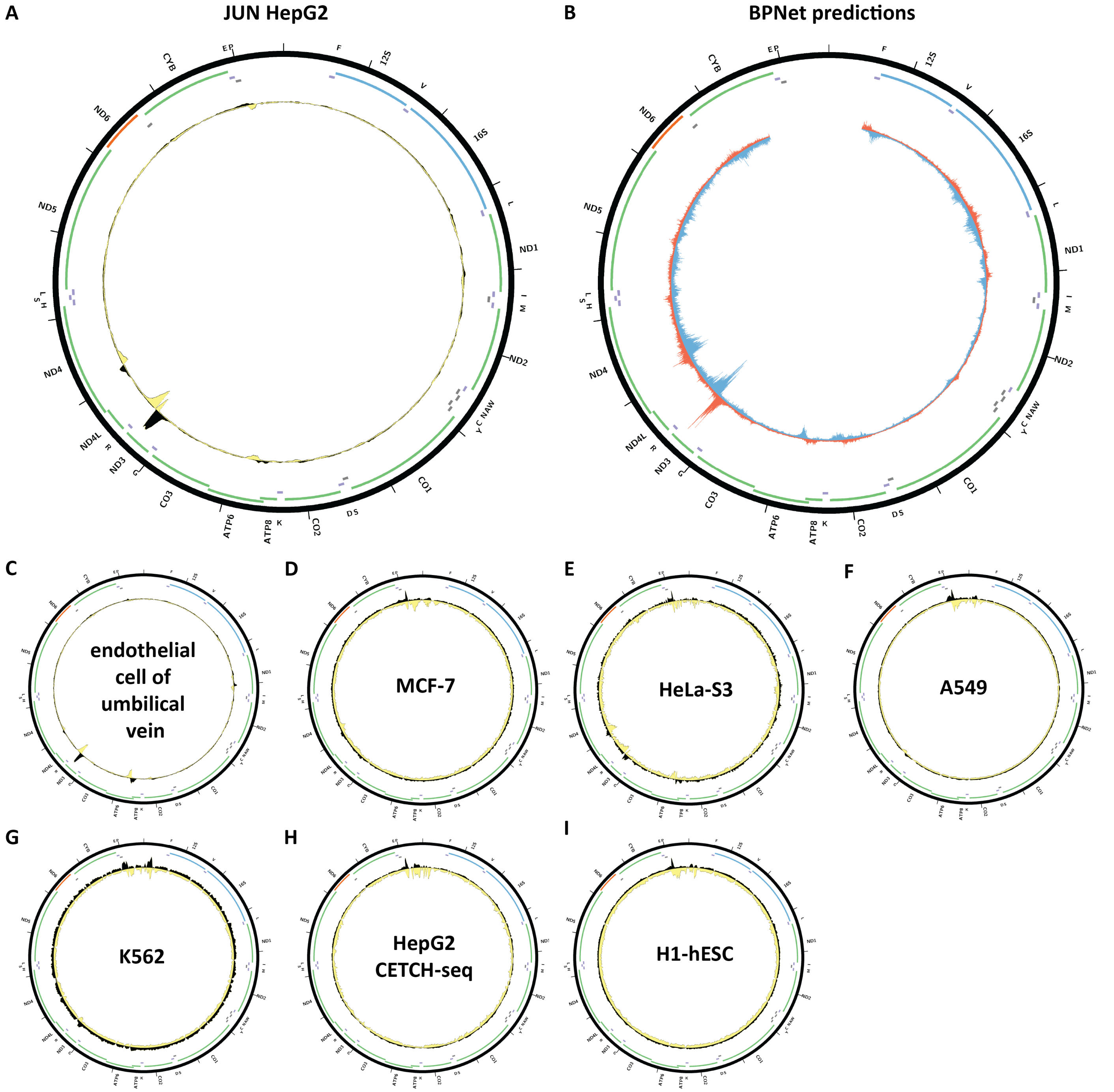

Supplementary Figure 13: Evidence for mitochondrial genome occupancy by the JUN transcription factor. Black and yellow tracks show the forward- and reverse-strand ChIP-seq coverage over chrM. (A) HepG2 ChIP-seq (ENCODE ID ENCSR000EEK; antibody: Santa Cruz Biotech sc-1694, Lot ID C2206); (B) BPNet predictions over chrM (ENCODE ID ENCSR701HFV); (C) endothelial cell of umbilical vein ChIP-seq (ENCODE ID ENCSR000EFA; antibody: Santa Cruz Biotech sc-1694, Lot ID C2206); (D) MCF-7 ChIP-seq (ENCODE ID ENCSR176EXN; antibody: Santa Cruz Biotech sc-1694, Lot ID C2206); (E) HeLa-S3 ChIP-seq (ENCODE ID ENCSR000EDG; antibody: Santa Cruz Biotech sc-1694, Lot ID C2206); (F) A549 ChIP-seq (ENCODE ID ENCSR996DUT; antibody: Santa Cruz Biotech sc-1694, Lot ID C2206); (G) K562 ChIP-seq (ENCODE ID ENCSR000EFS; antibody: Santa Cruz Biotech sc-1694, Lot ID C2206); (H) HepG2 CETCH-seq (ENCODE ID ENCSR747VUU); (I) H1-hESC ChIP-seq (ENCODE ID ENCSR000ECA; antibody: Santa Cruz Biotech sc-1694, Lot ID C2206).

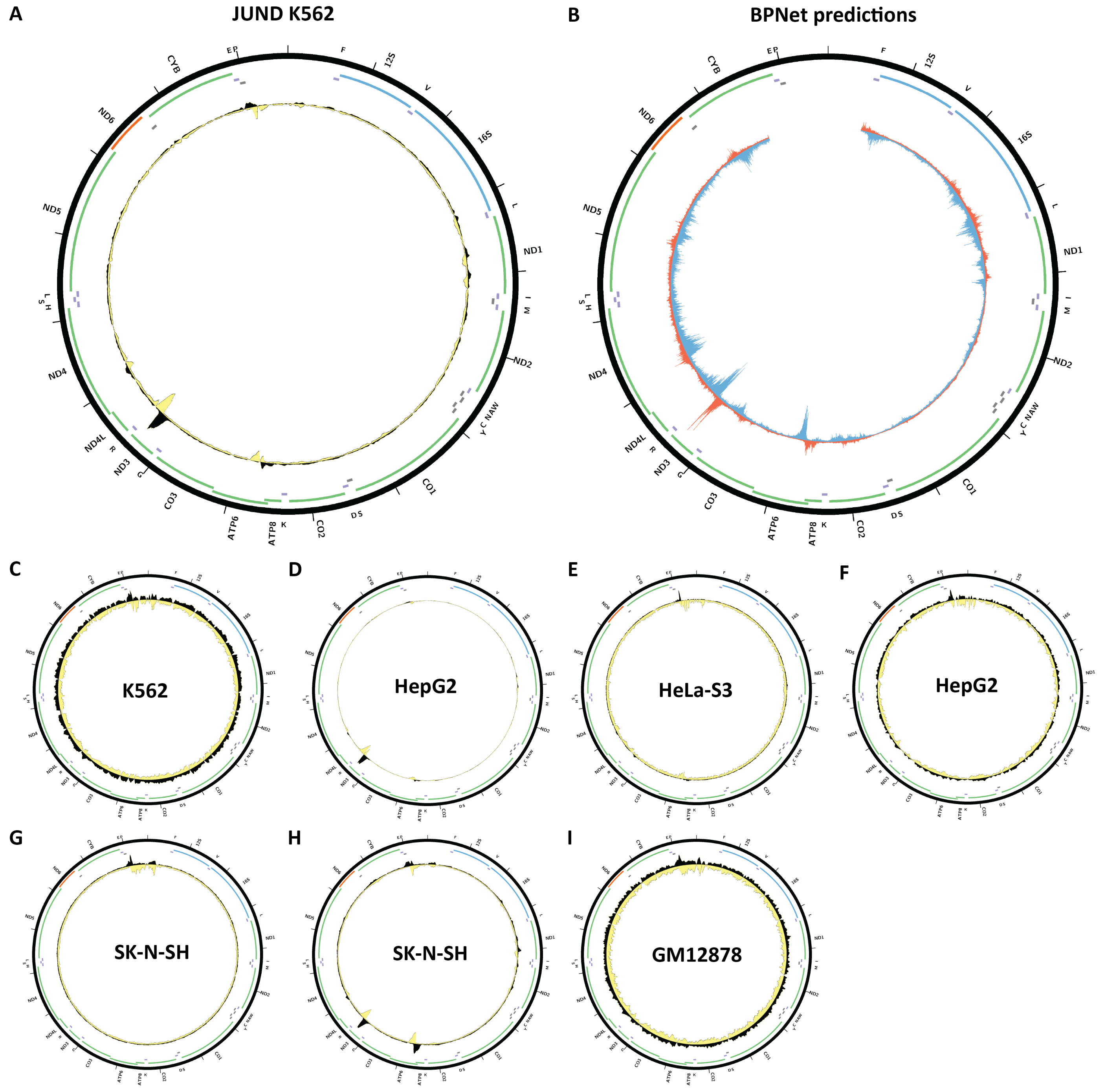

Supplementary Figure 14: Evidence for mitochondrial genome occupancy by the JUND transcription factor. Black and yellow tracks show the forward- and reverse-strand ChIP-seq coverage over chrM. (A) K562 ChIP-seq (ENCODE ID ENCSR000DJX; GFP-tagged); (B) BPNet predictions over chrM (ENCODE ID ENCSR767UUB); (C) K562 ChIP-seq (ENCODE ID ENCSR000DJX; antibody: GFP-tagged); (D) HepG2 ChIP-seq (ENCODE ID ENCSR000EEI; antibody: Santa Cruz Biotech sc-74); (E) HeLa-S3 ChIP-seq (ENCODE ID ENCSR000EDH; antibody: Santa Cruz Biotech sc-74); (F) HepG2 ChIP-seq (ENCODE ID ENCSR000BGK; antibody: Santa Cruz Biotech sc-74); (G) SK-N-SH ChIP-seq (ENCODE ID ENCSR000BSK; antibody: Santa Cruz Biotech sc-74); (H) SK-N-SH ChIP-seq (ENCODE ID ENCSR000EIB; antibody: Santa Cruz Biotech sc-74); (I) GM18278 ChIP-seq (ENCODE ID ENCSR000DYS; antibody: Santa Cruz Biotech sc-74).

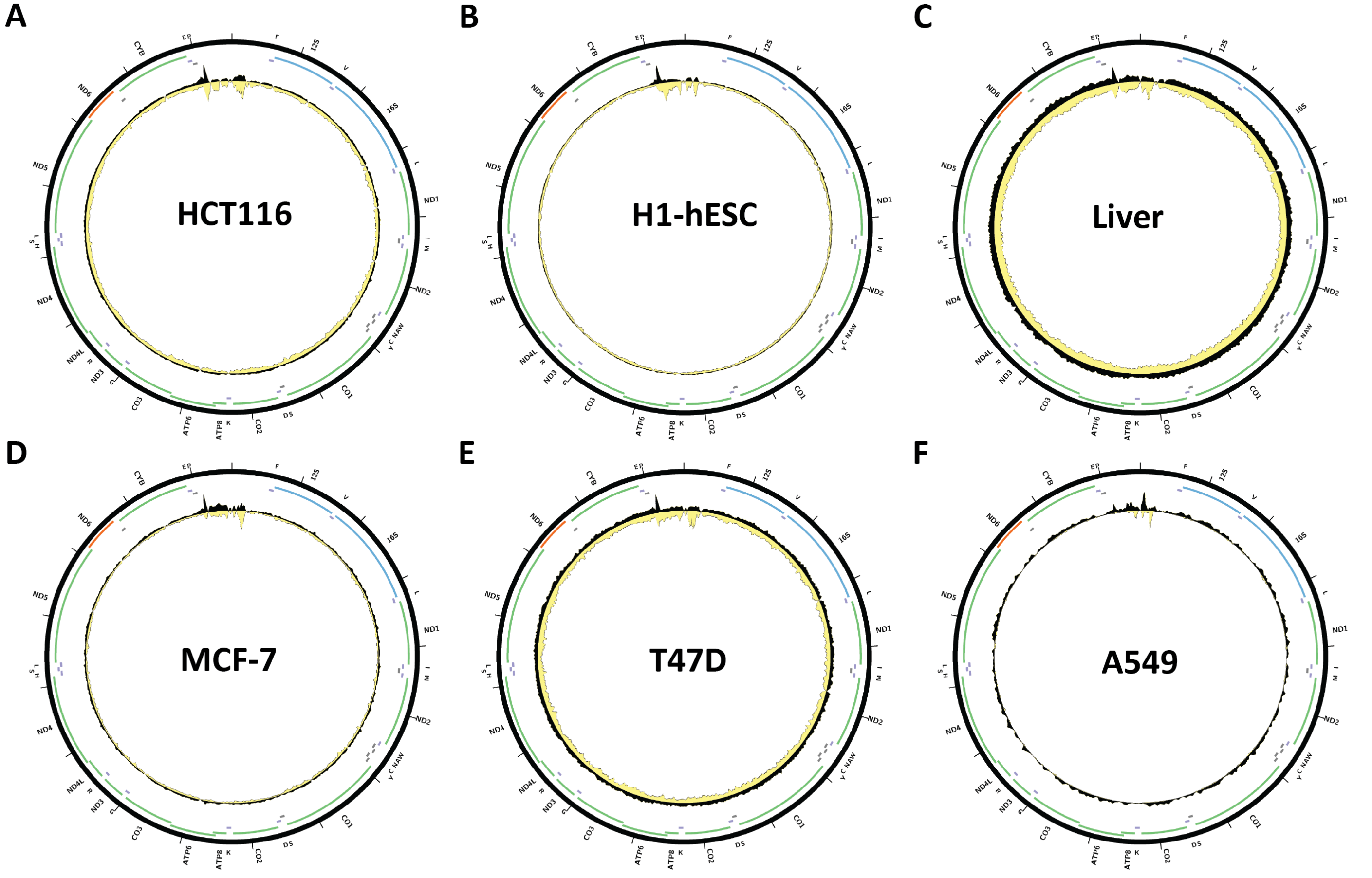

Supplementary Figure 15: Evidence for mitochondrial genome occupancy by the JUND transcription factor. Black and yellow tracks show the forward- and reverse-strand ChIP-seq coverage over chrM. (A) HCT116 ChIP-seq (ENCODE ID ENCSR000BSA; antibody: Santa Cruz Biotech sc-74); (B) H1-hESC ChIP-seq (ENCODE ID ENCSR000BKP; antibody: Santa Cruz Biotech sc-74); (C) Liver ChIP-seq (ENCODE ID ENCSR837GTK; antibody: Santa Cruz Biotech sc-74); (D) MCF-7 ChIP-seq (ENCODE ID ENCSR000BSU; antibody: Santa Cruz Biotech sc-74); (E) T47D ChIP-seq (ENCODE ID ENCSR000BVO; antibody: Santa Cruz Biotech sc-74); (F) A549 ChIP-seq (ENCODE ID ENCSR000BRF; antibody: Santa Cruz Biotech sc-74).

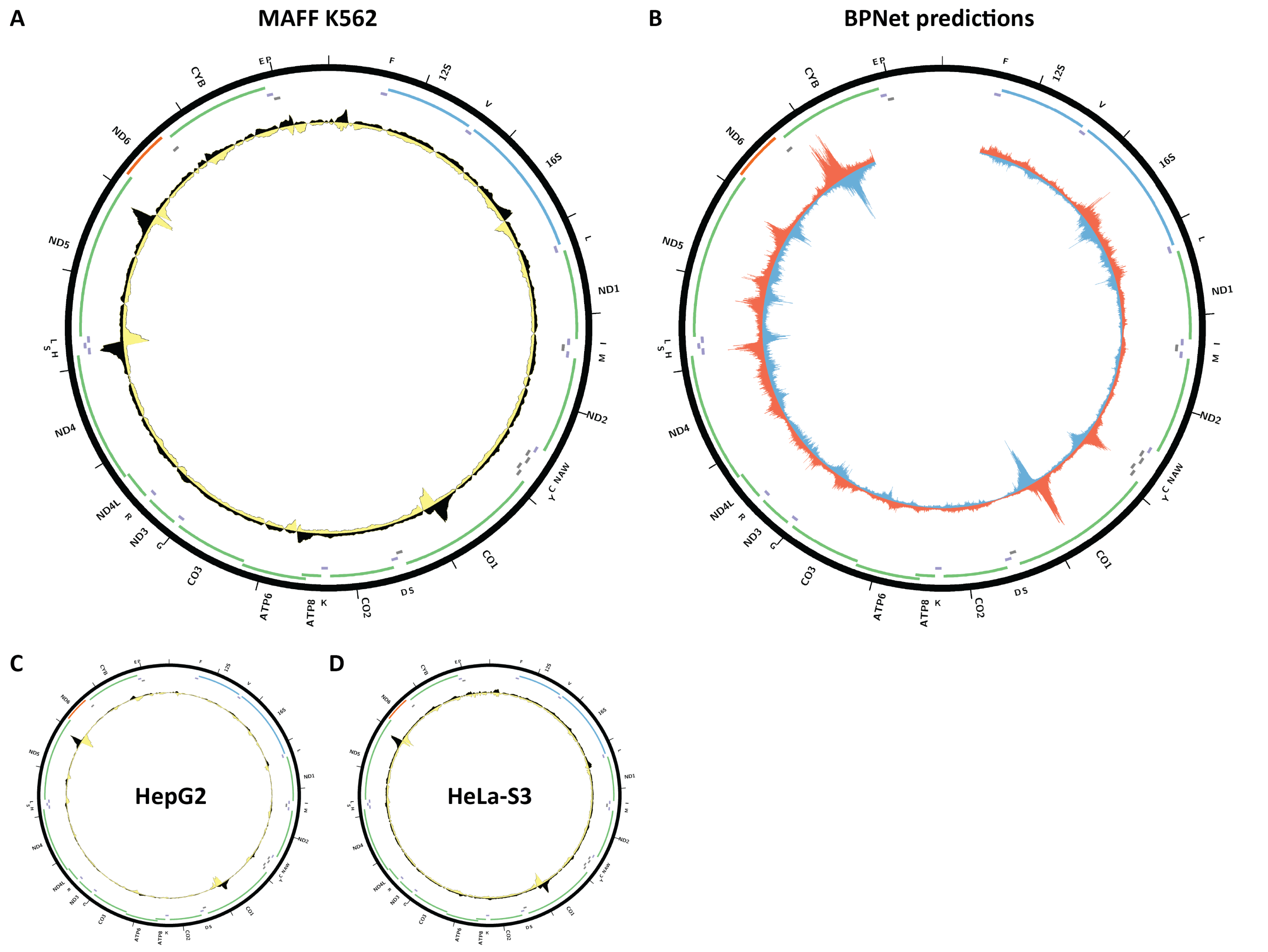

Supplementary Figure 16: Evidence for mitochondrial genome occupancy by the MAFF transcription factor. Black and yellow tracks show the forward- and reverse-strand ChIP-seq coverage over chrM. (A) K562 ChIP-seq (ENCODE ID ENCSR000EGI; antibody: Sigma M8194, Lot ID 125K4837); (B) BPNet predictions over chrM (ENCODE ID ENCSR512XRO); (C) HepG2 ChIP-seq (ENCODE ID ENCSR000EEC; antibody: Sigma M8194, Lot ID 125K4837); (D) HeLa-S3 ChIP-seq (ENCODE ID ENCSR140DSL; antibody: Sigma M8194, Lot ID 125K4837).

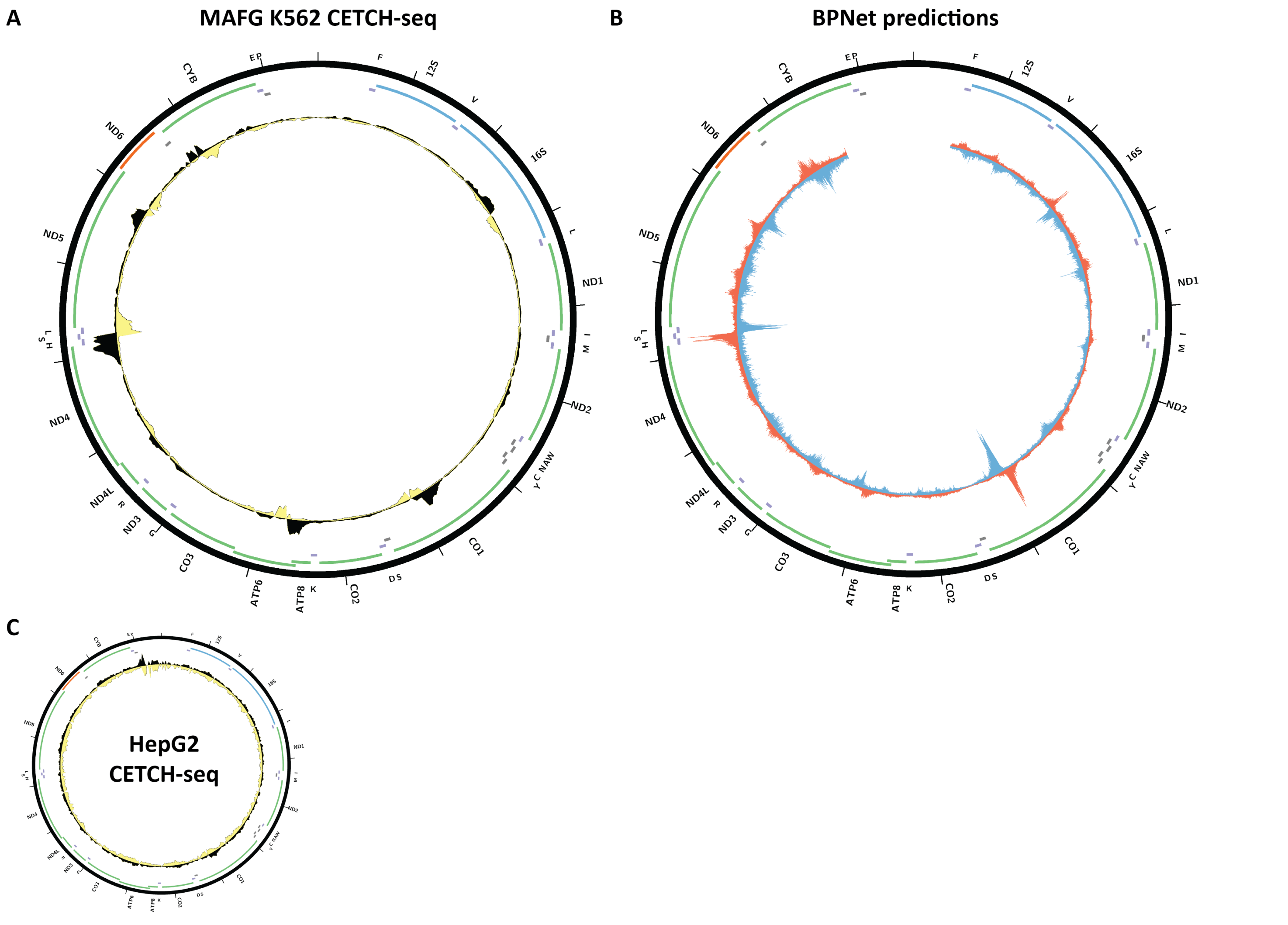

Supplementary Figure 17: Evidence for mitochondrial genome occupancy by the MAFG transcription factor. Black and yellow tracks show the forward- and reverse-strand ChIP-seq coverage over chrM. (A) K562 CETCH-seq (ENCODE ID ENCSR818DQV); (B) BPNet predictions over chrM (ENCODE ID ENCSR324HYA); (C) HepG2 CETCH-seq (ENCODE ID ENCSR708KAA).

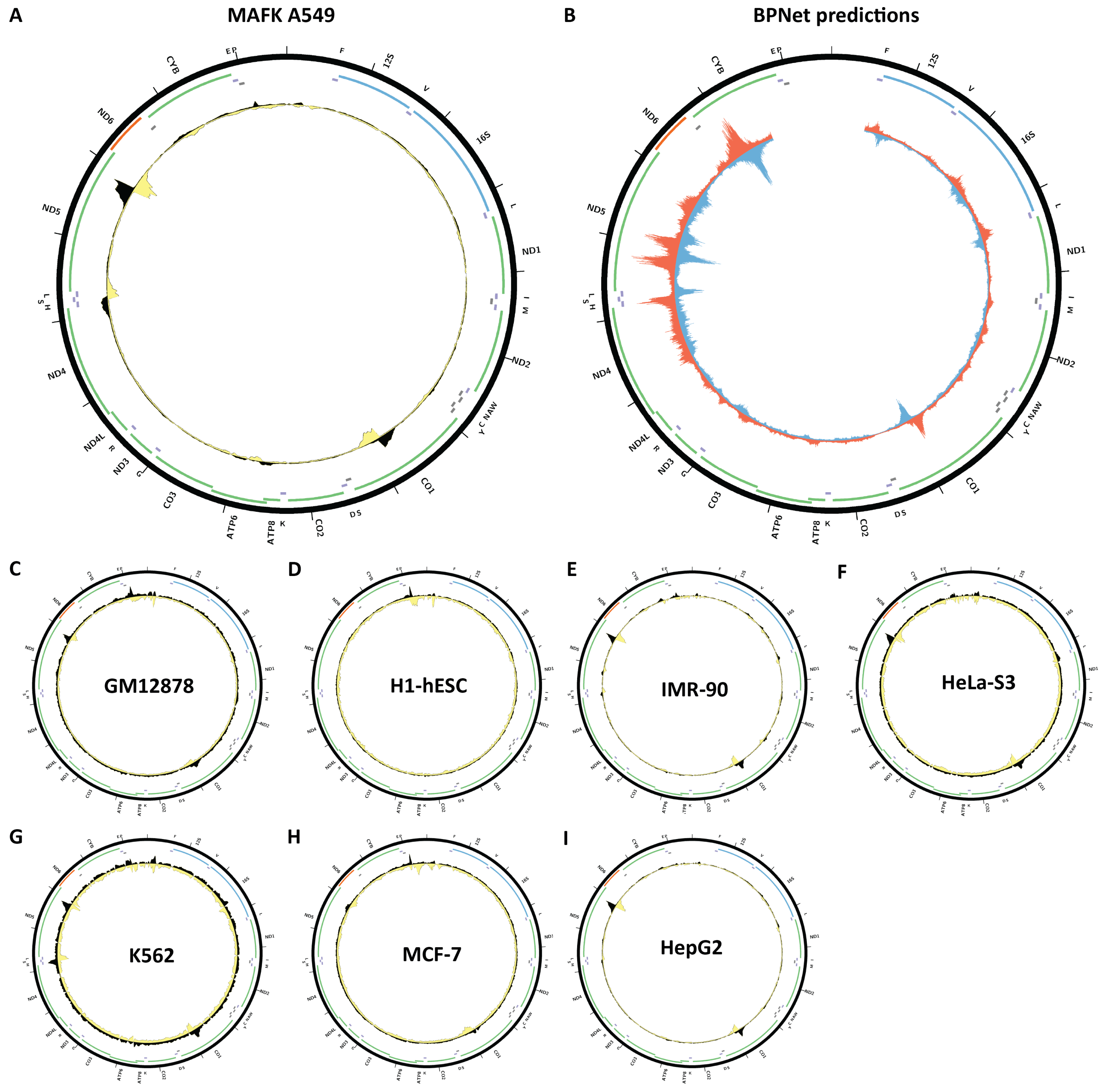

Supplementary Figure 18: Evidence for mitochondrial genome occupancy by the MAFK transcription factor. Black and yellow tracks show the forward- and reverse-strand ChIP-seq coverage over chrM. (A) A549 ChIP-seq (ENCODE ID ENCSR541WQI; antibody: Abcam ab50322, Lot ID 904274); (B) BPNet predictions over chrM (ENCODE ID ENCSR415PWX); (C) GM18278 ChIP-seq (ENCODE ID ENCSR000DYV; antibody: Abcam ab50322, Lot ID 904274); (D) H1-hESC ChIP-seq (ENCODE ID ENCSR000EBS; antibody: Abcam ab50322, Lot ID 904274); (E) IMR-90 ChIP-seq (ENCODE ID ENCSR000EFH; antibody: Abcam ab50322, Lot ID 904274); (F) HeLa-S3 ChIP-seq (ENCODE ID ENCSR000ECK; antibody: Abcam ab50322, Lot ID 904274); (G) K562 ChIP-seq (ENCODE ID ENCSR000EGX; antibody: Abcam ab50322, Lot ID 904274); (H) MCF-7 ChIP-seq (ENCODE ID ENCSR555PBN; antibody: Abcam ab50322, Lot ID 904274); (I) HepG2 ChIP-seq (ENCODE ID ENCSR000EDZ; antibody: Santa Cruz Biotech sc-477, Lot ID K1709).

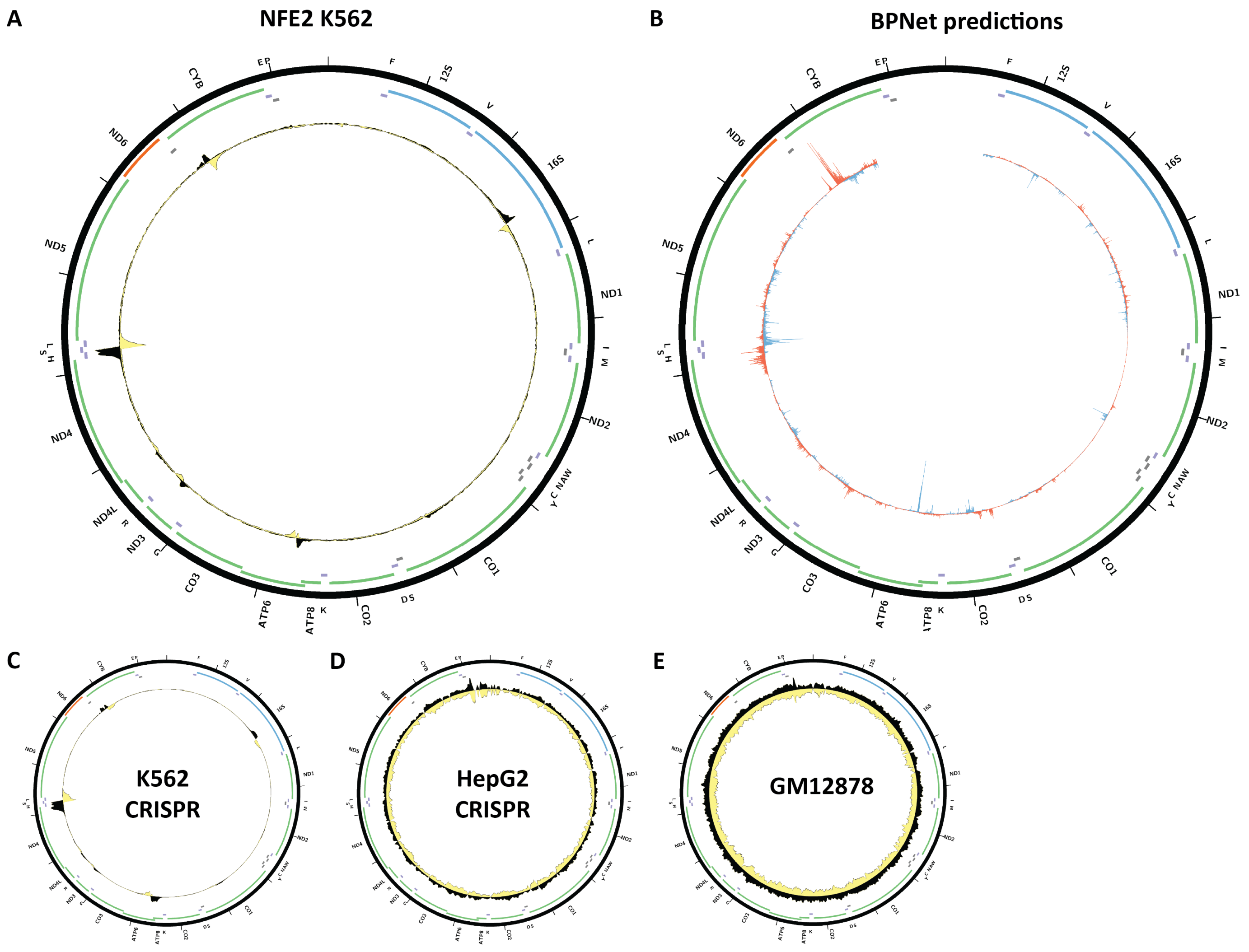

Supplementary Figure 19: Evidence for mitochondrial genome occupancy by the NFE2 transcription factor. Black and yellow tracks show the forward- and reverse-strand ChIP-seq coverage over chrM. (A) K562 ChIP-seq (ENCODE ID ENCSR000FAF; antibody Santa Cruz Biotech sc-291, Lot ID D1703); (B) BPNet predictions over chrM (ENCODE ID ENCSR833AMA); (C) K562 CETCH-seq (ENCODE ID ENCSR552YGL); (C) HepG2 CETCH-seq (ENCODE ID ENCSR983FBD); (C) GM12878 ChIP-seq (ENCODE ID ENCSR000DZY; antibody Santa Cruz Biotech sc-22827).

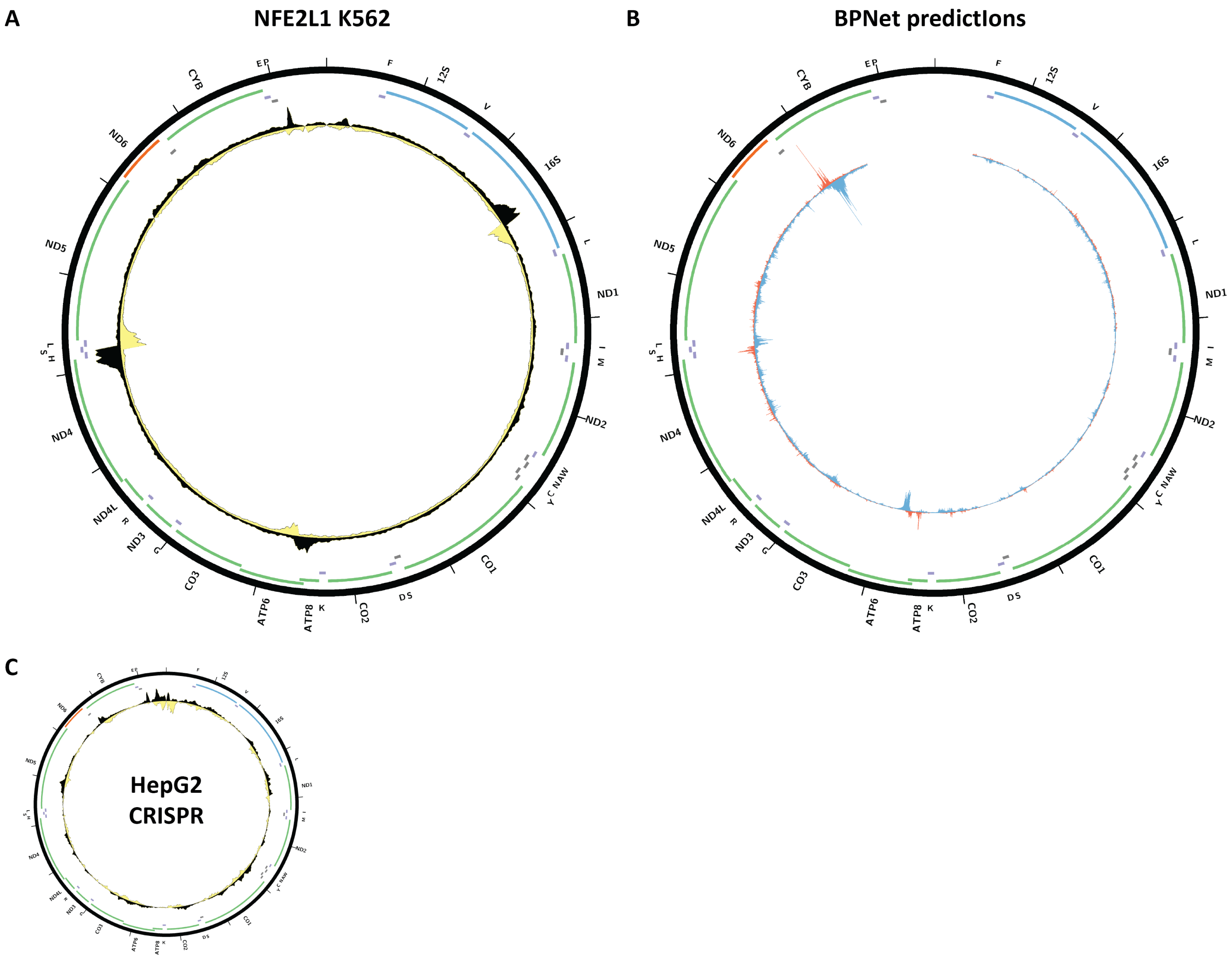

Supplementary Figure 20: Evidence for mitochondrial genome occupancy by the NFE2L1 transcription factor. Black and yellow tracks show the forward- and reverse-strand ChIP-seq coverage over chrM. (A) K562 ChIP-seq (ENCODE ID ENCSR632SHZ; GFP-tagged); (B) BPNet predictions over chrM; (C) HepG2 CETCH-seq (ENCODE ID ENCSR543SBE; antibody:).

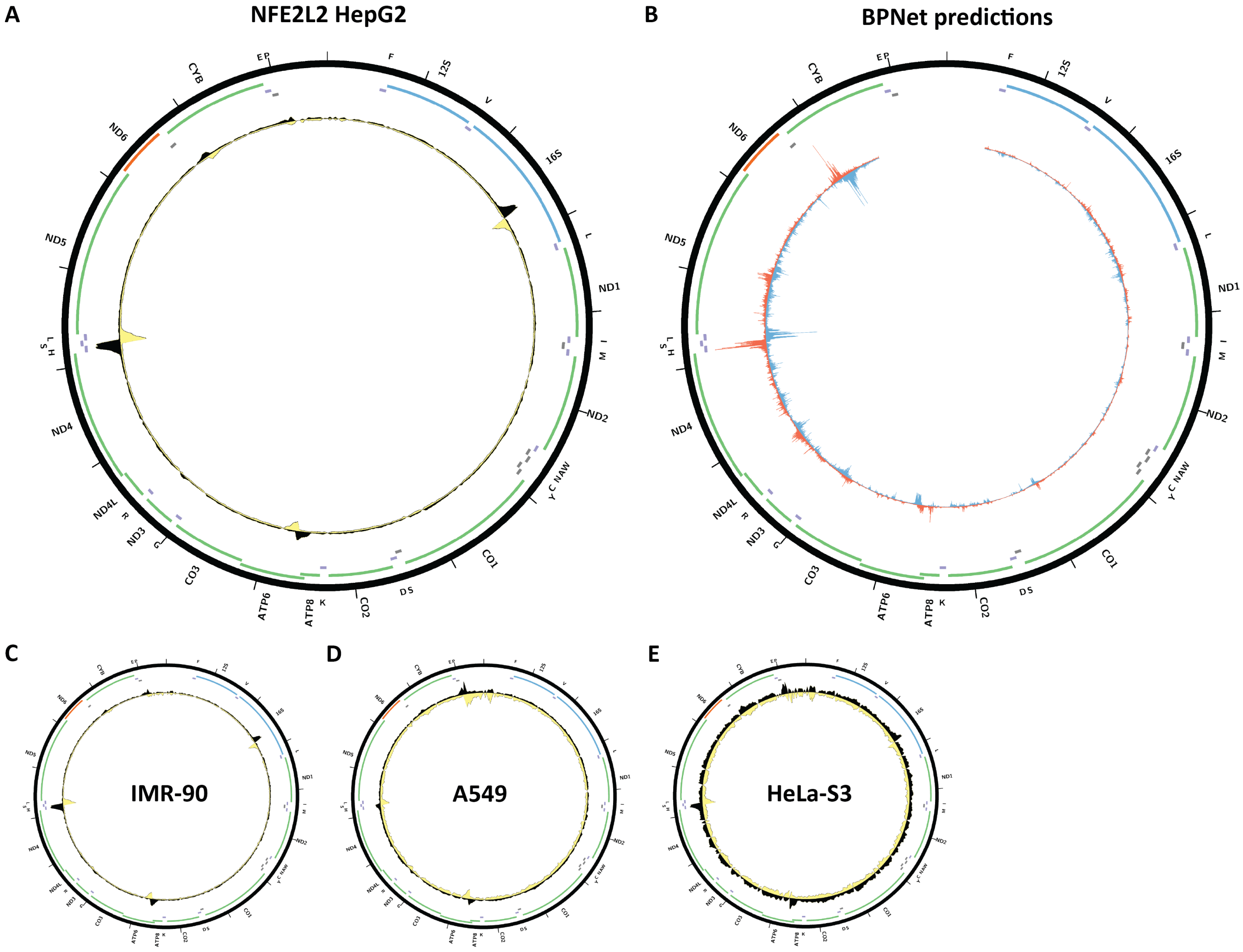

Supplementary Figure 21: Evidence for mitochondrial genome occupancy by the NFE2L2 transcription factor. Black and yellow tracks show the forward- and reverse-strand ChIP-seq coverage over chrM. (A) HepG2 ChIP-seq (ENCODE ID ENCSR488EES; antibody: Santa Cruz Biotech sc-13032, Lot ID A1711); (B) BPNet predictions over chrM (ENCODE ID ENCSR301SDD); (C) IMR-90 ChIP-seq (ENCODE ID ENCSR197WGI; antibody: Santa Cruz Biotech sc-13032, Lot ID A1711); (D) A549 ChIP-seq (ENCODE ID ENCSR584GHV; antibody: Santa Cruz Biotech sc-13032, Lot ID A1711); (E) HeLa-S3 ChIP-seq (ENCODE ID ENCSR707IUN; antibody: Santa Cruz Biotech sc-13032, Lot ID A1711).

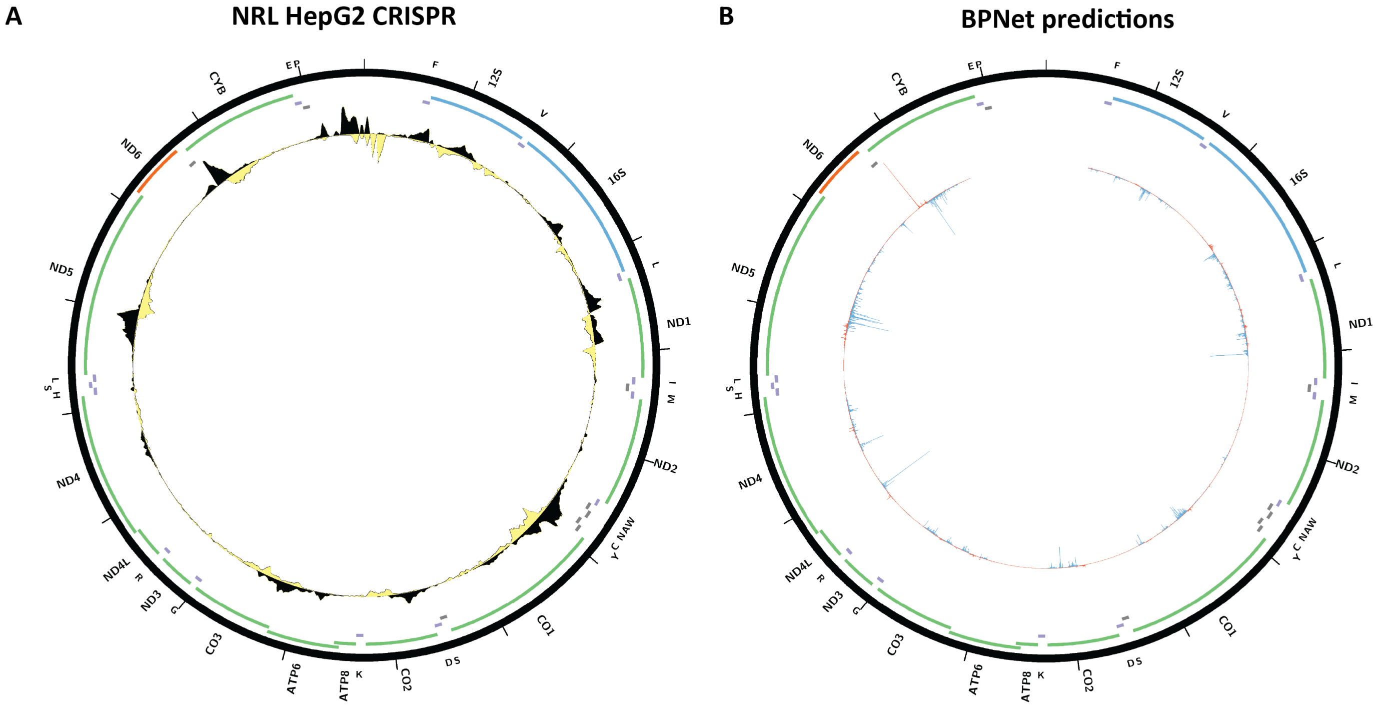

Supplementary Figure 22: Evidence for mitochondrial genome occupancy by the NRL transcription factor. Black and yellow tracks show the forward- and reverse-strand ChIP-seq coverage over chrM. (A) HepG2 CETCH-seq (ENCODE ID ENCSR518KLO); (B) BPNet predictions over chrM.

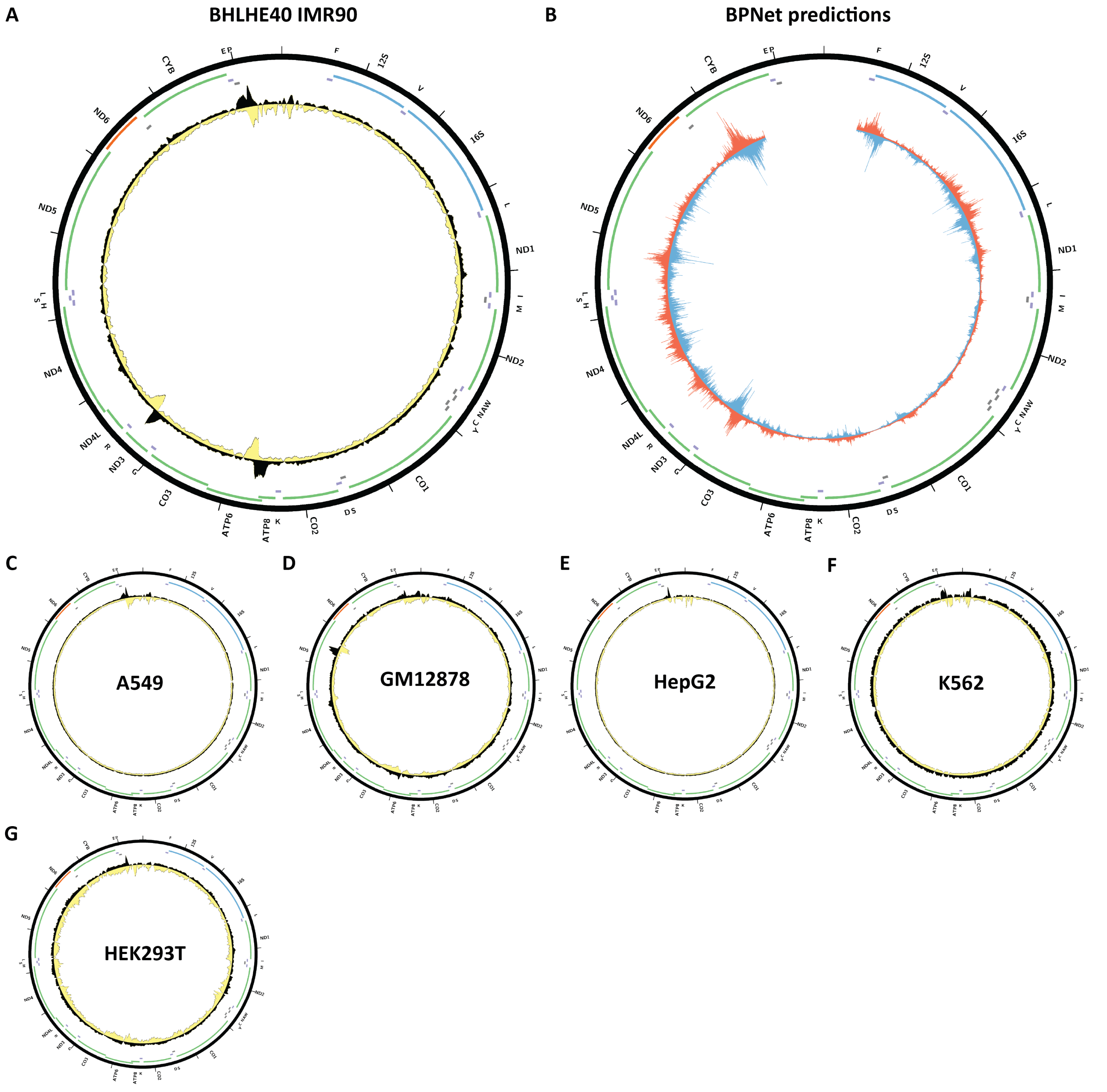

Supplementary Figure 23: Evidence for mitochondrial genome occupancy by the BHLHE40 transcription factor. Black and yellow tracks show the forward- and reverse-strand ChIP-seq coverage over chrM. (A) IMR90 ChIP-seq (ENCODE ID ENCSR957KYB; antibody: Novus NB100-1800, Lot ID A1); (B) BPNet predictions over chrM (ENCODE ID ENCSR695LTU); (C) A549 ChIP-seq (ENCODE ID ENCSR000DYJ; antibody: Santa Cruz Biotech sc-101023); (D) GM12878 ChIP-seq (ENCODE ID ENCSR987MTA; antibody: Novus NB100-1800, Lot ID A1); (E) HepG2 ChIP-seq (ENCODE ID ENCSR000BID; antibody: Santa Cruz Biotech sc-101023); (F) K562 ChIP-seq (ENCODE ID ENCSR000EGV; antibody: Novus NB100-1800, Lot ID A1); (G) HEK293T ChIP-seq (ENCODE ID ENCSR789GVU; antibody: Novus NB100-1800, Lot ID A1).

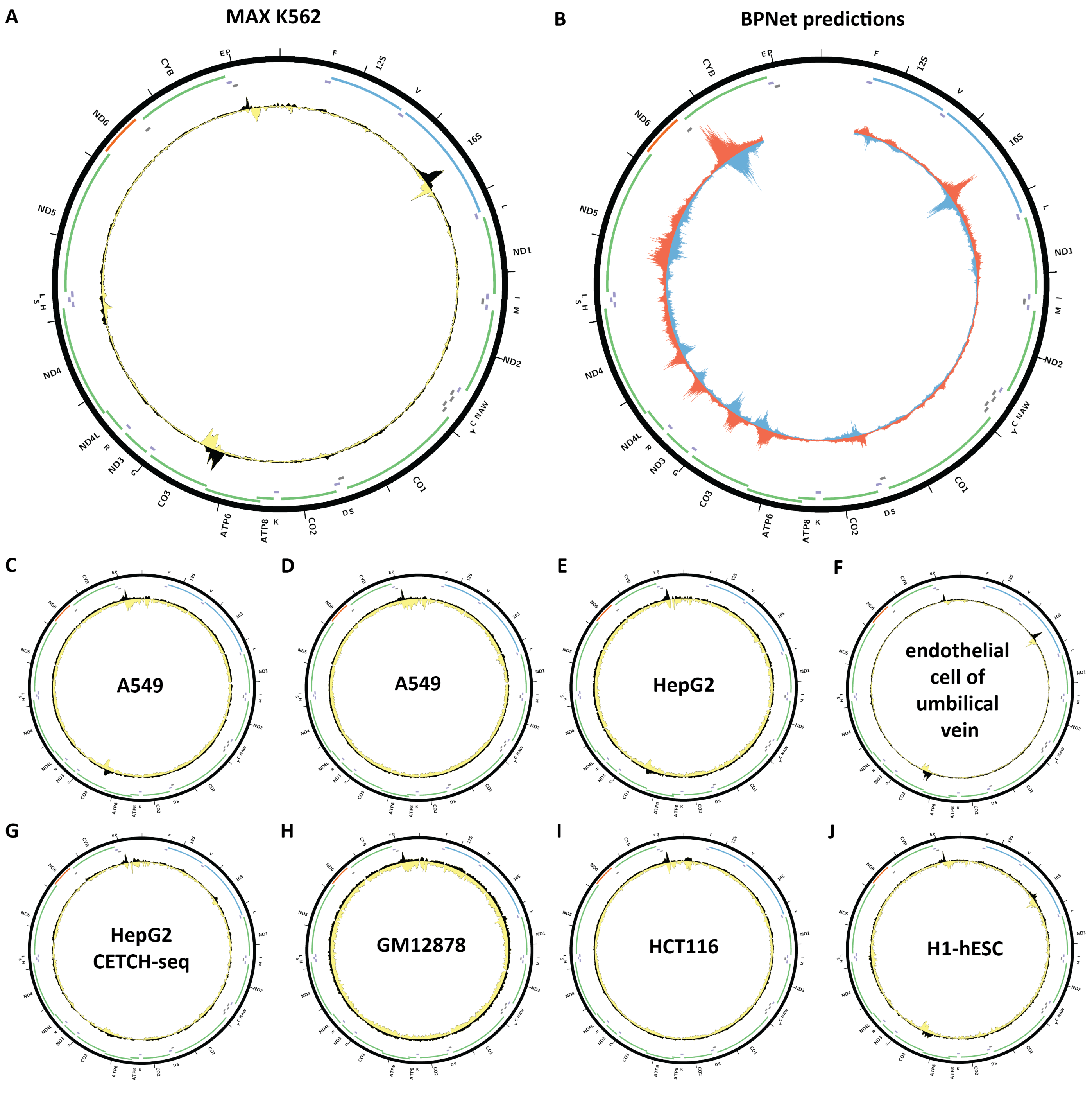

Supplementary Figure 24: Evidence for mitochondrial genome occupancy by the MAX transcription factor. Black and yellow tracks show the forward- and reverse-strand ChIP-seq coverage over chrM. (A) K562 ChIP-seq (ENCODE ID ENCSR000FAE; antibody: Santa Cruz Biotech sc-197, Lot ID J0809); (B) BPNet predictions over chrM (ENCODE ID ENCSR445ZHD); (C) A549 ChIP-seq (ENCODE ID ENCSR000DYG; antibody: Santa Cruz Biotech sc-197, Lot ID J0809); (D) A549 ChIP-seq (ENCODE ID ENCSR000BTJ; antibody: Santa Cruz Biotech sc-197, Lot ID J0809); (E) HepG2 ChIP-seq (ENCODE ID ENCSR000EDS; antibody: Santa Cruz Biotech sc-197, Lot ID J0809); (F) endothelial cell of umbilical vein ChIP-seq (ENCODE ID ENCSR000EEZ; antibody: Santa Cruz Biotech sc-197, Lot ID J0809); (G) HepG2 CETCH-seq (ENCODE ID ENCSR168DYA); (H) GM18278 ChIP-seq (ENCODE ID ENCSR000DZF; antibody: Santa Cruz Biotech sc-197, Lot ID J0809); (I) HCT116 ChIP-seq (ENCODE ID ENCSR000BSH; antibody: Santa Cruz Biotech sc-197, Lot ID J0809); (J) H1-hESC ChIP-seq (ENCODE ID ENCSR000EUP; antibody: Santa Cruz Biotech sc-197, Lot ID J0809).

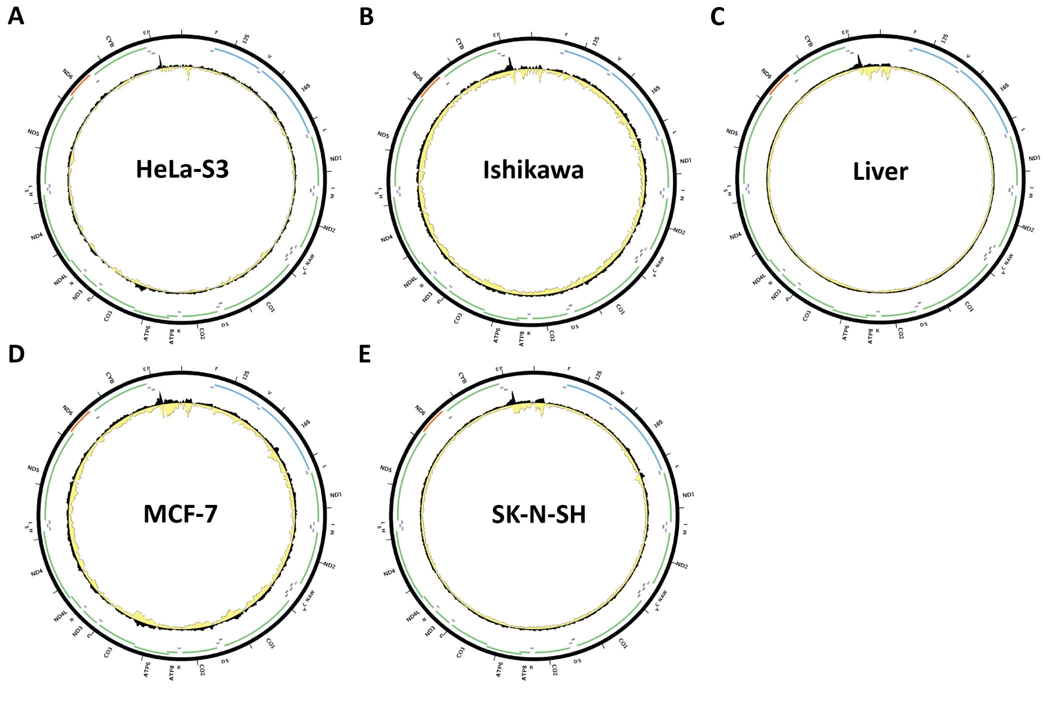

Supplementary Figure 25: Evidence for mitochondrial genome occupancy by the MAX transcription factor. Black and yellow tracks show the forward- and reverse-strand ChIP-seq coverage over chrM. (A) HeLa-S3 ChIP-seq (ENCODE ID ENCSR000EZF; antibody: Santa Cruz Biotech sc-197, Lot ID J0809); (B) Ishikawa ChIP-seq (ENCODE ID ENCSR000BTY; antibody: Santa Cruz Biotech sc-197, Lot ID J0809); (C) Liver ChIP-seq (ENCODE ID ENCSR521IID; antibody: Santa Cruz Biotech sc-197, Lot ID J0809); (D) MCF-7 ChIP-seq (ENCODE ID ENCSR000BUL; antibody: Santa Cruz Biotech sc-197, Lot ID J0809); (E) SK-N-SH ChIP-seq (ENCODE ID ENCSR000BVD; antibody: Santa Cruz Biotech sc-197, Lot ID J0809).

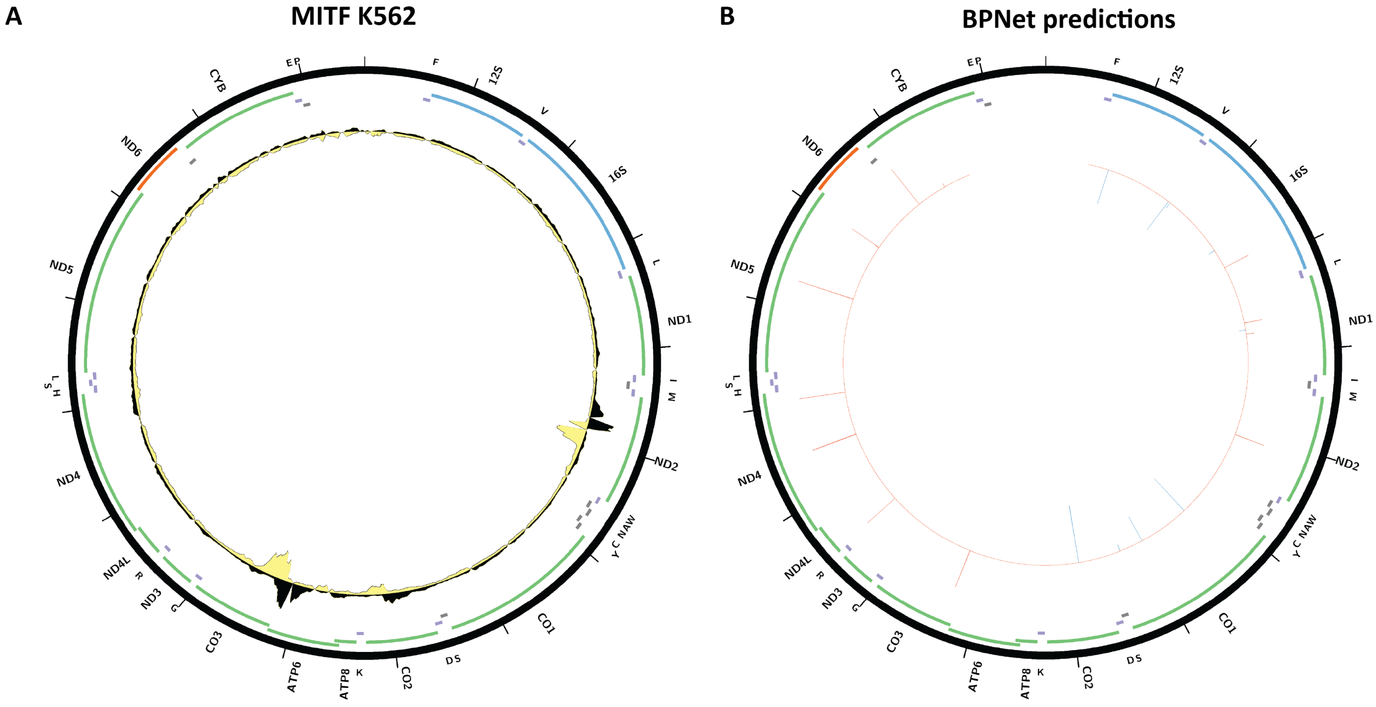

Supplementary Figure 26: Evidence for mitochondrial genome occupancy by the MITF transcription factor. Black and yellow tracks show the forward- and reverse-strand ChIP-seq coverage over chrM. (A) K562 ChIP-seq (ENCODE ID ENCSR797SWM; antibody: Active Motif 39789, Lot ID 11313002); (B) BPNet predictions over chrM (ENCODE ID ENCSR551LHV).

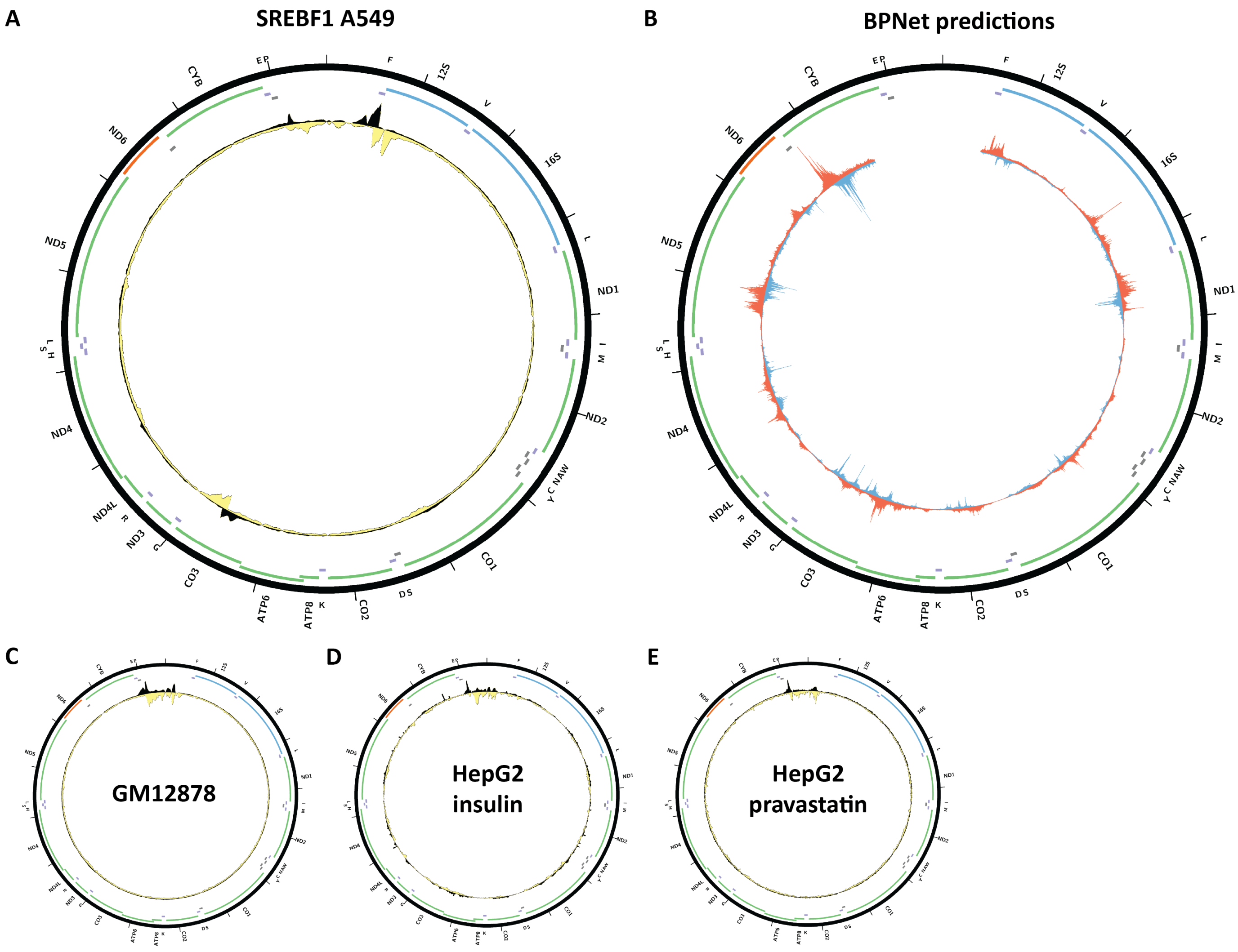

Supplementary Figure 27: Evidence for mitochondrial genome occupancy by the SREBF1 transcription factor. Black and yellow tracks show the forward- and reverse-strand ChIP-seq coverage over chrM. (A) A549 ChIP-seq (ENCODE ID ENCSR897MYK; antibody: Santa Cruz Biotech sc-8984, Lot ID 10211); (B) BPNet predictions over chrM (ENCODE ID ENCSR182NCE); (C) GM12878 ChIP-seq (ENCODE ID ENCSR000DYU; antibody: Santa Cruz Biotech sc-8984, Lot ID 10211); (D) HepG2 (10 μM insulin, 100 μM 22-hydroxycholesterol, 6 hours post-treatment) ChIP-seq (ENCODE ID ENCSR000EEO; antibody: Santa Cruz Biotech sc-8984, Lot ID 10211); (E) HepG2 (2 μM pravastatin, 16 hours post-treatment) ChIP-seq (ENCODE ID ENCSR000EZP; antibody: Santa Cruz Biotech sc-8984, Lot ID 10211).

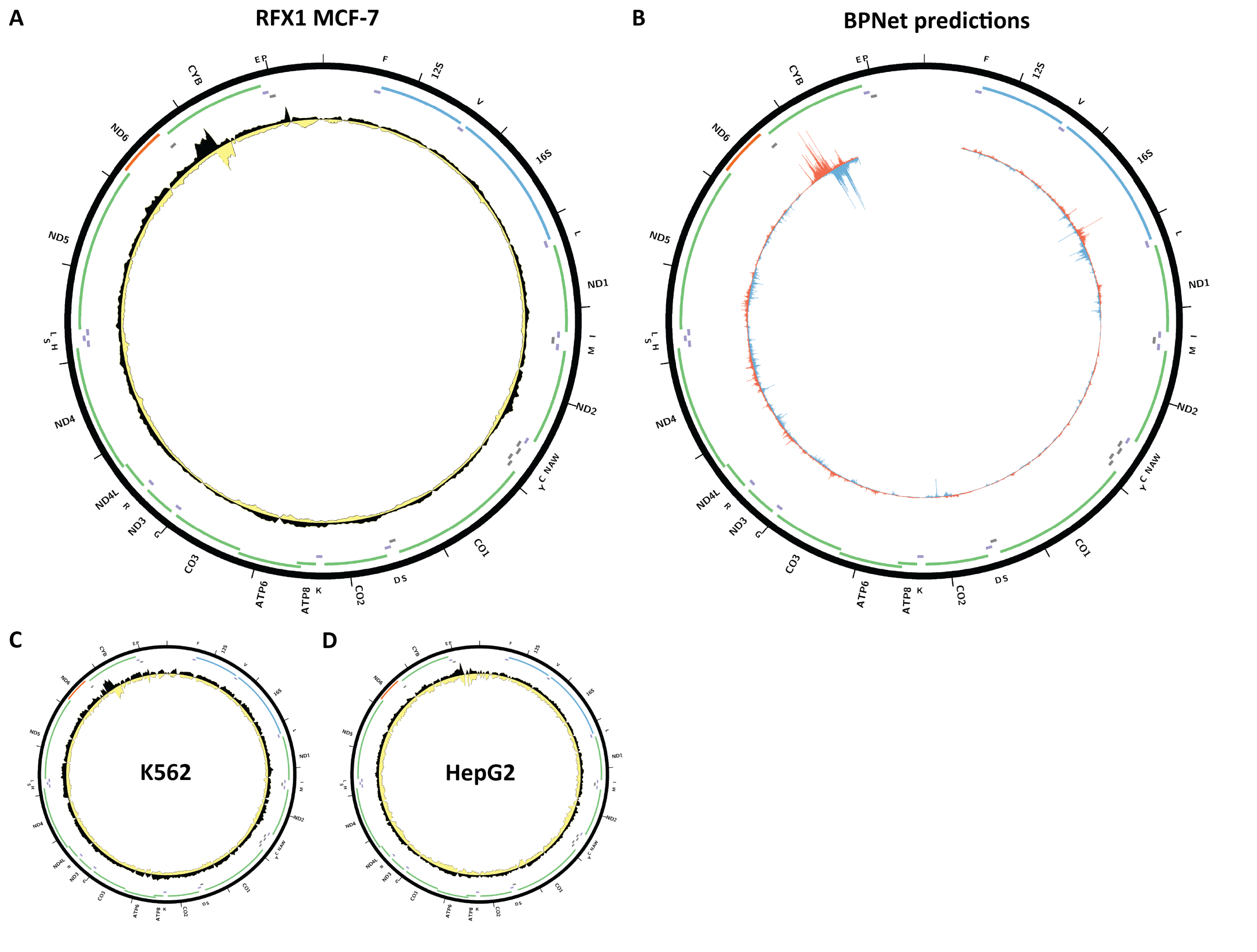

Supplementary Figure 28: Evidence for mitochondrial genome occupancy by the RFX1 transcription factor. Black and yellow tracks show the forward- and reverse-strand ChIP-seq coverage over chrM. (A) MCF-7 ChIP-seq (ENCODE ID ENCSR788XNX; antibody: Santa Cruz Biotech sc-10652, Lot ID E1911); (B) BPNet predictions over chrM (ENCODE ID ENCSR594OAT); (C) K562 ChIP-seq (ENCODE ID ENCSR968GIB; antibody: Santa Cruz Biotech sc-10652, Lot ID E1911); (D) HepG2 ChIP-seq (ENCODE ID ENCSR928API; antibody: Santa Cruz Biotech sc-10652, Lot ID E1911).

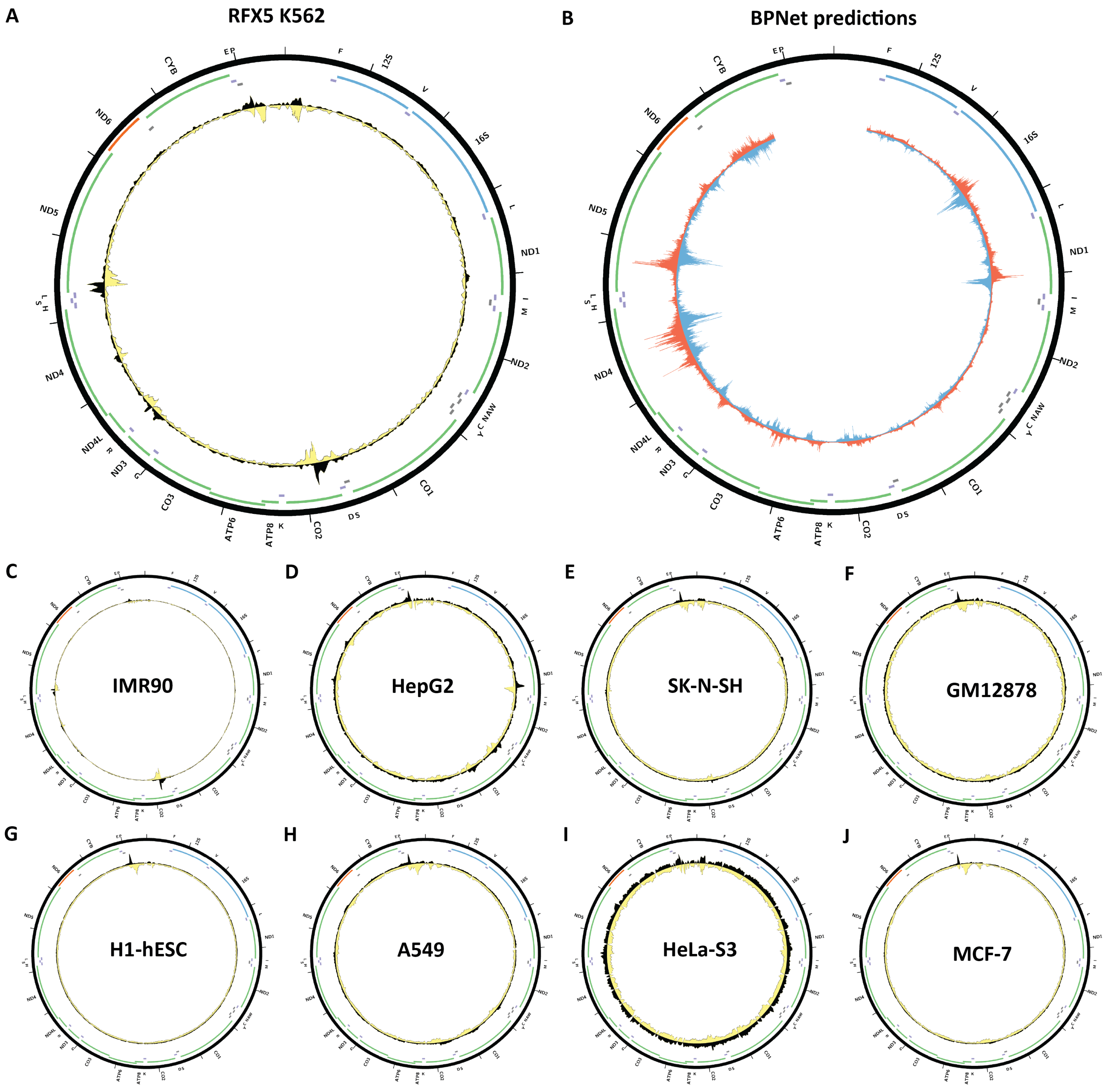

Supplementary Figure 29: Evidence for mitochondrial genome occupancy by the RFX5 transcription factor. Black and yellow tracks show the forward- and reverse-strand ChIP-seq coverage over chrM. (A) K562 ChIP-seq (ENCODE ID ENCSR000EGO; antibody: Rockland 200-401-194, Lot ID 14562); (B) BPNet predictions over chrM; (C) IMR-90 ChIP-seq (ENCODE ID ENCSR000EFD; antibody: Rockland 200-401-194, Lot ID 14562); (D) HepG2 ChIP-seq (ENCODE ID ENCSR000EEA; antibody: Rockland 200-401-194, Lot ID 14562); (E) SK-N-SH ChIP-seq (ENCODE ID ENCSR000EHY; antibody: Rockland 200-401-194, Lot ID 14562); (F) GM12878 ChIP-seq (ENCODE ID ENCSR000DZW; antibody: Rockland 200-401-194, Lot ID 14562); (G) H1-hESC ChIP-seq (ENCODE ID ENCSR000ECF; antibody: Rockland 200-401-194, Lot ID 14562); (H) A549 ChIP-seq (ENCODE ID ENCSR064LJN; antibody: Rockland 200-401-194, Lot ID 14562); (I) HeLa-S3 ChIP-seq (ENCODE ID ENCSR000ECX; antibody: Rockland 200-401-194, Lot ID 14562); (J) MCF-7 ChIP-seq (ENCODE ID ENCSR924TVL; antibody: Rockland 200-401-194, Lot ID 14562).

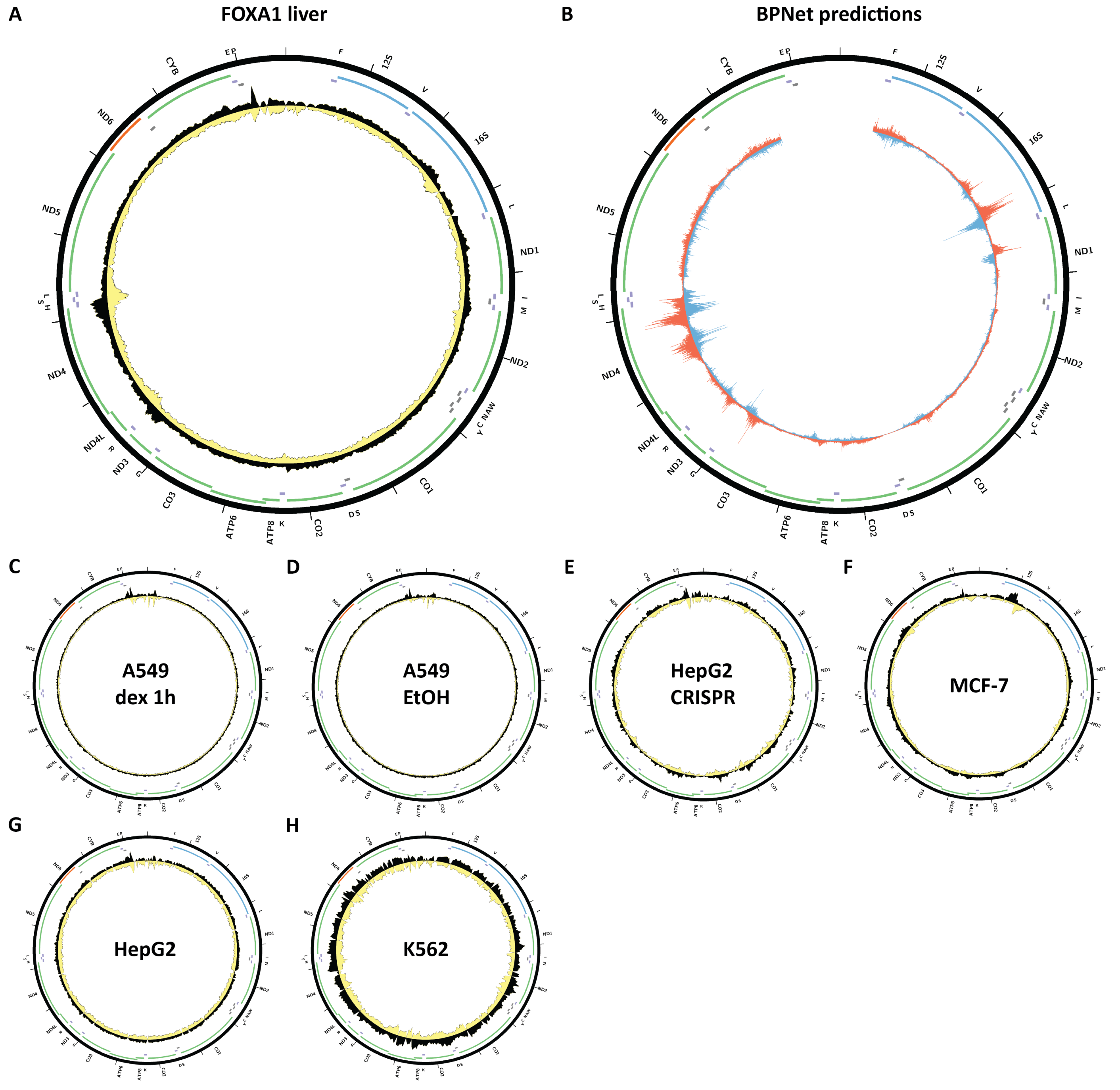

Supplementary Figure 30: Evidence for mitochondrial genome occupancy by the FOXA1 transcription factor. Black and yellow tracks show the forward- and reverse-strand ChIP-seq coverage over chrM. (A) Liver ChIP-seq (ENCODE ID ENCSR324RCI; antibody: Santa Cruz Biotech sc-6553, Lot ID H1209); (B) BPNet predictions over chrM (ENCODE ID ENCSR578NTH); (C) A549 Dex 1 hours ChIP-seq (ENCODE ID ENCSR000BPX; antibody: Santa Cruz Biotech sc-101058, Lot ID A2706); (D) A549 EtOH 1 hour ChIP-seq (ENCODE ID ENCSR000BRD; antibody: Santa Cruz Biotech sc-101058, Lot ID A2706); (E) HepG2 CETCH-seq (ENCODE ID ENCSR865RXA); (F) MCF-7 ChIP-seq (ENCODE ID ENCSR126YEB; antibody: GeneTex GTX100308, Lot ID 39435); (G) HepG2 ChIP-seq (ENCODE ID ENCSR000BLE; antibody: Santa Cruz Biotech sc-6553, Lot ID H1209); (H) K562 ChIP-seq (ENCODE ID ENCSR819LHG; antibody: GeneTex GTX100308, Lot ID 39435).

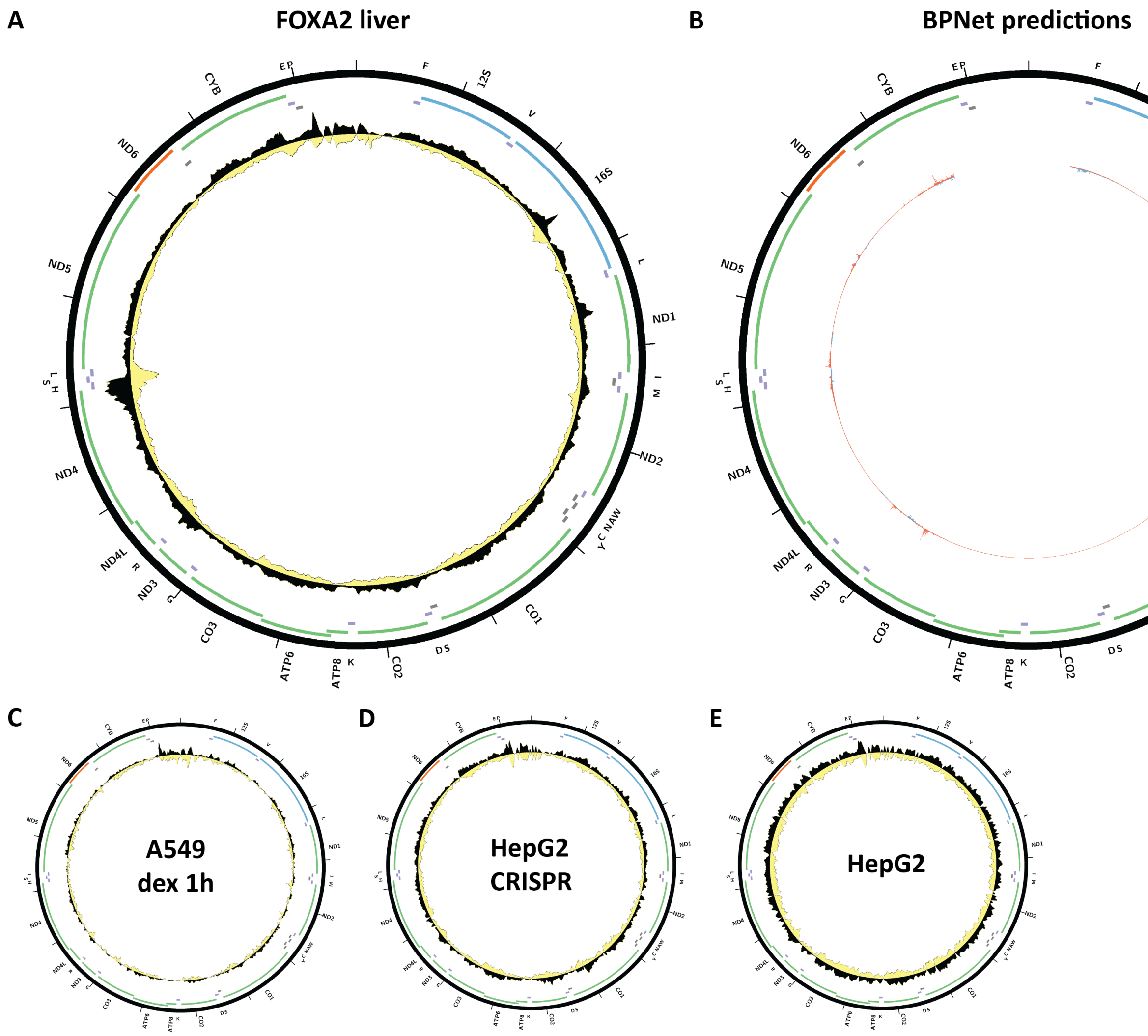

Supplementary Figure 31: Evidence for mitochondrial genome occupancy by the FOXA2 transcription factor. Black and yellow tracks show the forward- and reverse-strand ChIP-seq coverage over chrM. (A) Liver ChIP-seq (ENCODE ID ENCSR080XEY; antibody: Santa Cruz Biotech sc-6554, Lot ID L1409); (B) BPNet predictions over chrM (ENCODE ID ENCSR318CMD); (C) A549 Dex 1 hours ChIP-seq (ENCODE ID ENCSR000BRE; antibody: Santa Cruz Biotech sc-6554, Lot ID L1409); (D) HepG2 CETCH-seq (ENCODE ID ENCSR490AMH); (E) HepG2 ChIP-seq (ENCODE ID ENCSR000BNI; antibody: Santa Cruz Biotech sc-6554, Lot ID L1409).

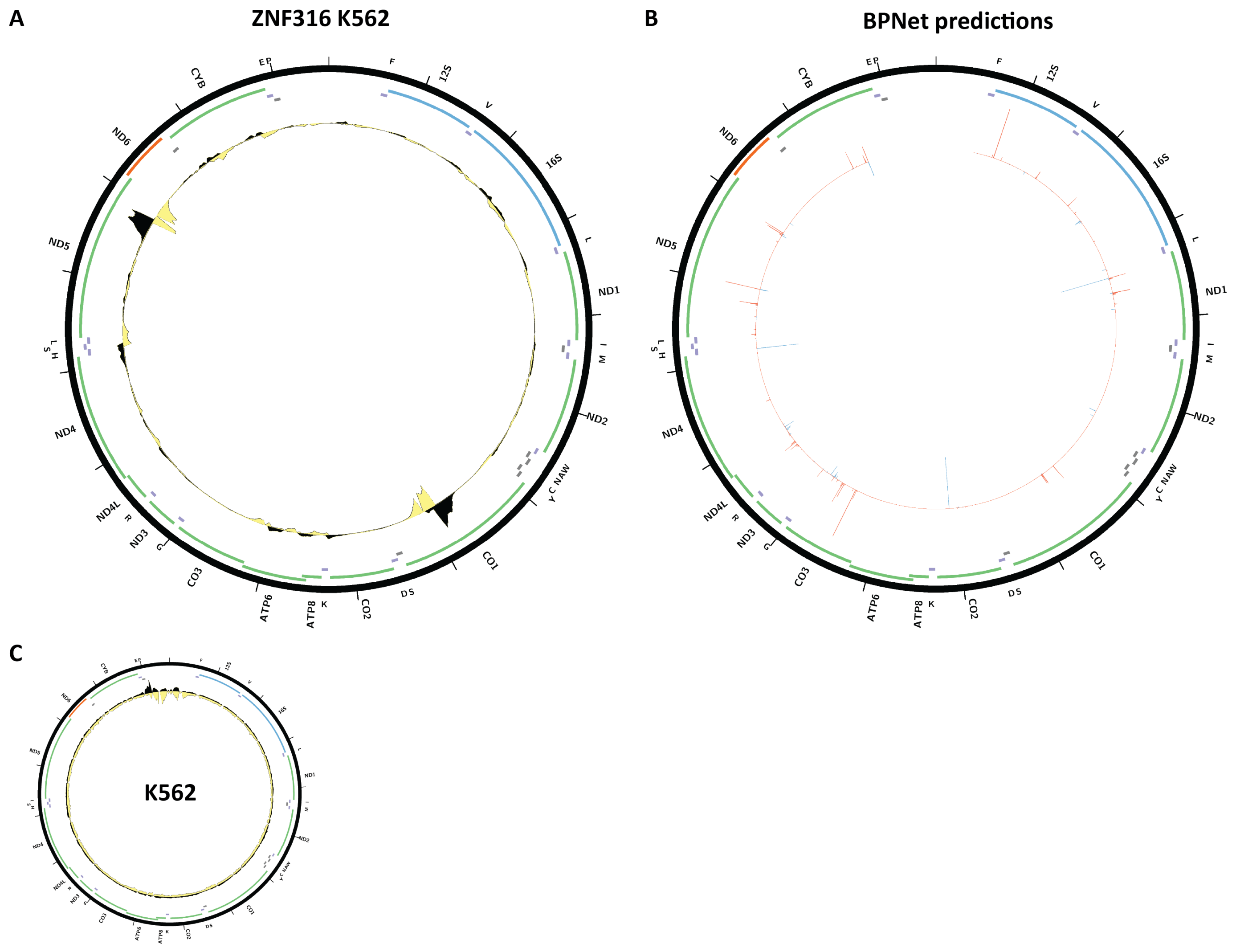

Supplementary Figure 32: Evidence for mitochondrial genome occupancy by the ZNF316 transcription factor. Black and yellow tracks show the forward- and reverse-strand ChIP-seq coverage over chrM. (A) K562 ChIP-seq (ENCODE ID ENCSR167KBO; antibody: Bethyl Labs A303-248A); (B) BPNet predictions over chrM; (C) K562 ChIP-seq (ENCODE ID ENCSR200JYP; antibody: Bethyl Labs A303-249A).

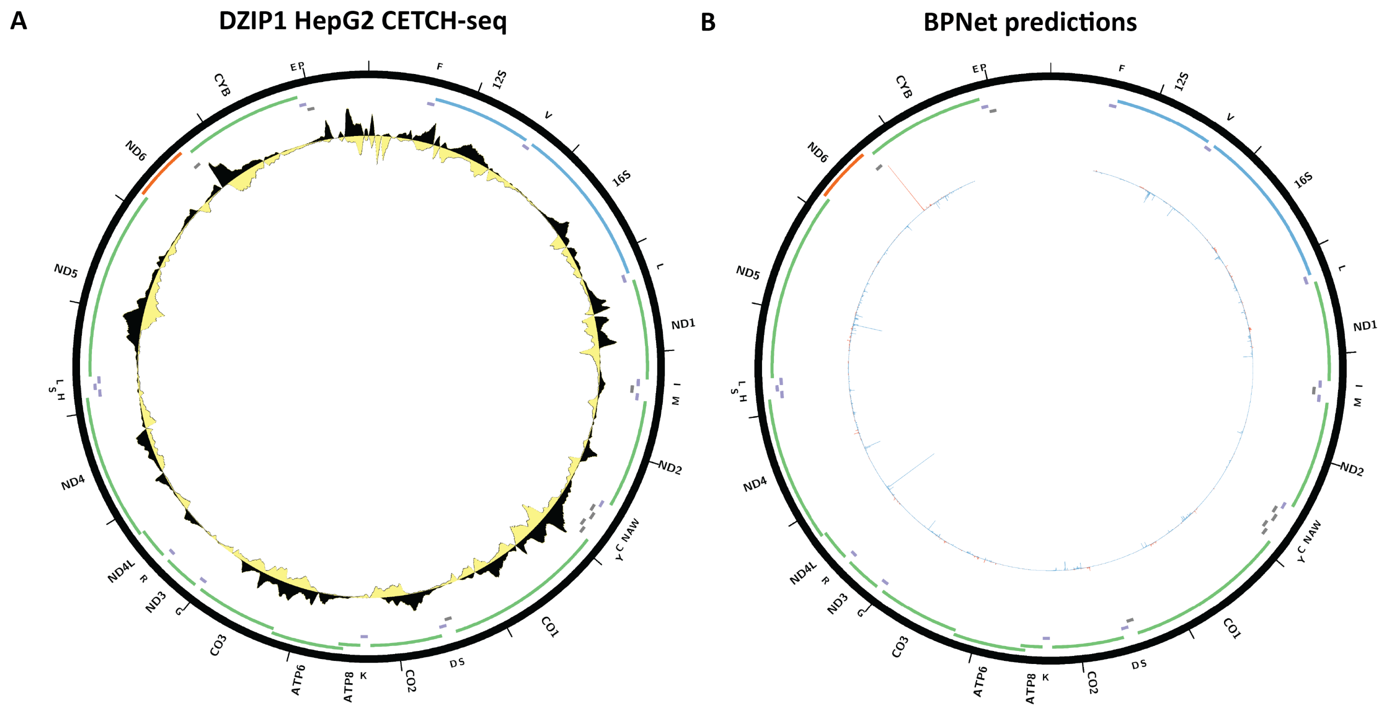

Supplementary Figure 33: Evidence for mitochondrial genome occupancy by the DZIP1 transcription factor. Black and yellow tracks show the forward- and reverse-strand ChIP-seq coverage over chrM. (A) HepG2 CETCH-seq (ENCODE ID ENCSR895KNN); (B) BPNet predictions over chrM.

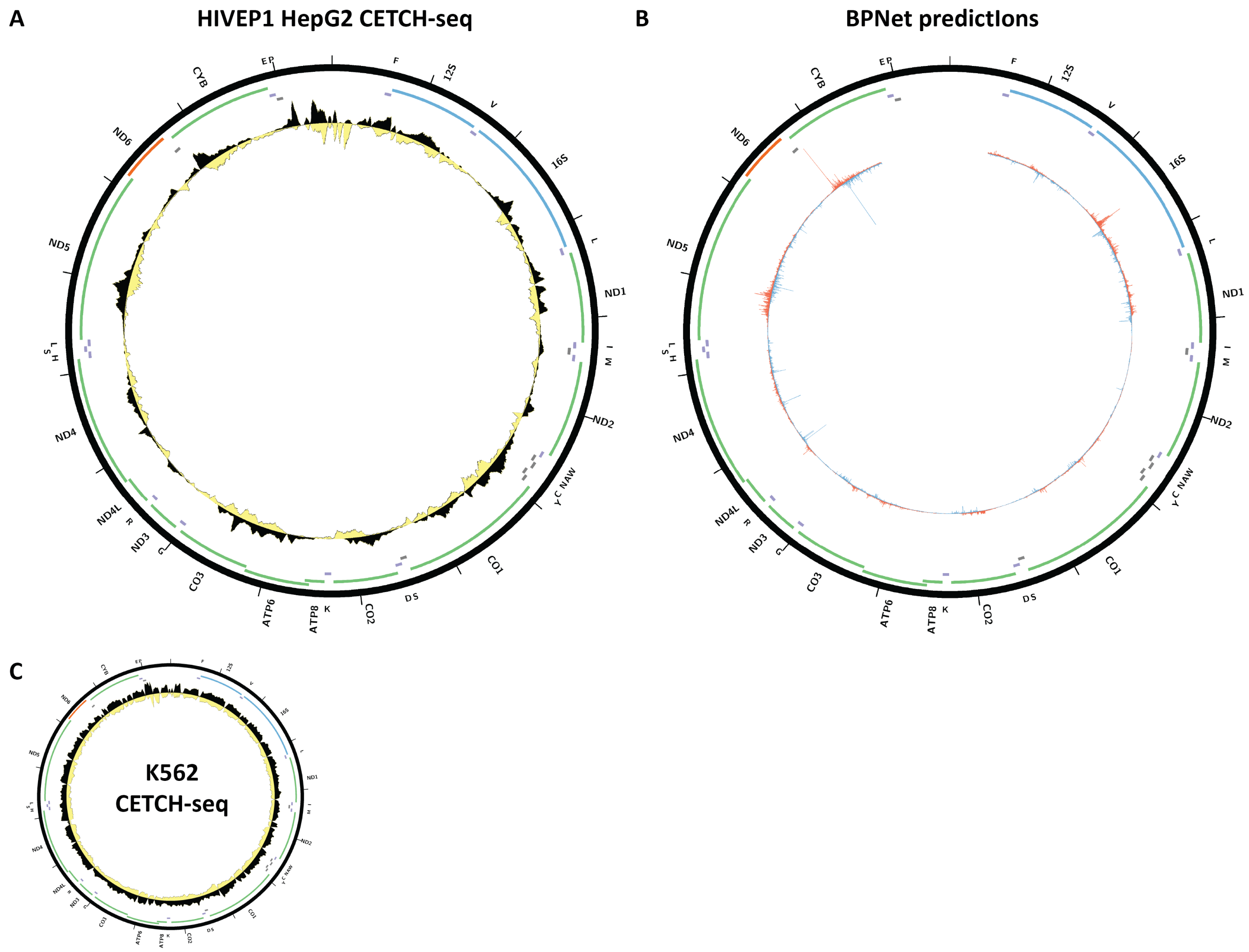

Supplementary Figure 34: Evidence for mitochondrial genome occupancy by the HIVEP1 transcription factor. Black and yellow tracks show the forward- and reverse-strand ChIP-seq coverage over chrM. (A) HepG2 CETCH-seq (ENCODE ID ENCSR697WMX); (B) BPNet predictions over chrM (ENCODE ID ENCSR272FZX); (C) K562 CETCH-seq (ENCODE ID ENCSR947PJZ).

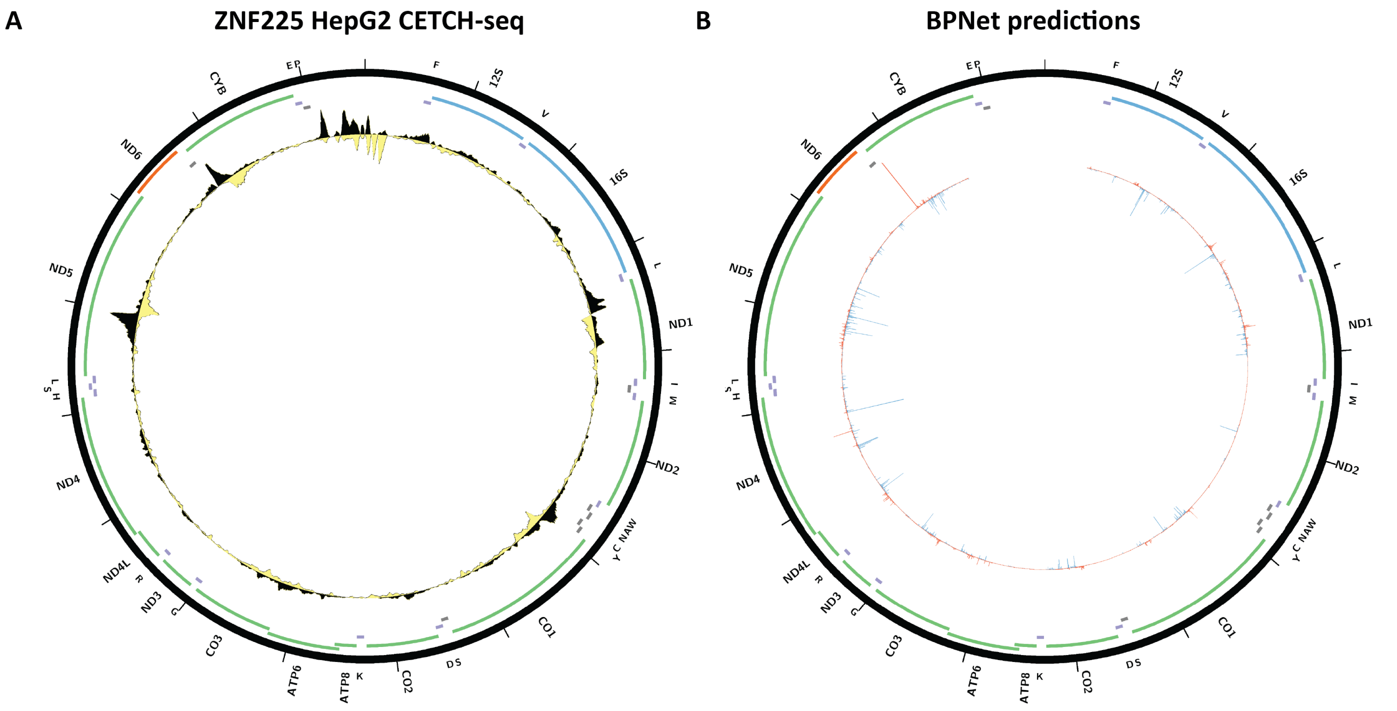

Supplementary Figure 35: Evidence for mitochondrial genome occupancy by the ZNF225 transcription factor. Black and yellow tracks show the forward- and reverse-strand ChIP-seq coverage over chrM. (A) HepG2 CETCH-seq (ENCODE ID ENCSR075PWK); (B) BPNet predictions over chrM.

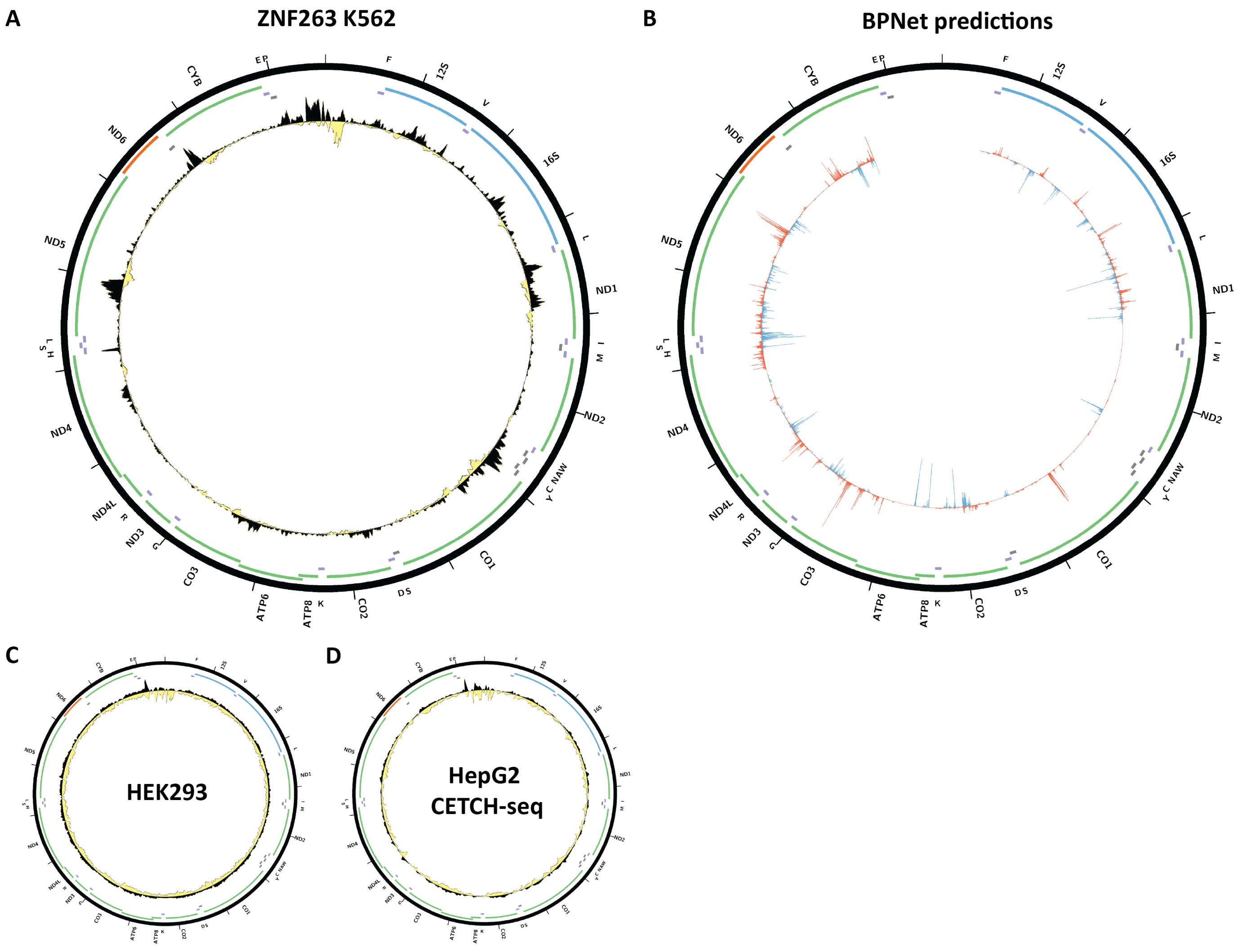

Supplementary Figure 36: Evidence for mitochondrial genome occupancy by the ZNF263 transcription factor. Black and yellow tracks show the forward- and reverse-strand ChIP-seq coverage over chrM. (A) K562 ChIP-seq (ENCODE ID ENCSR000EWN; antibody: Novus H00010127-A01); (B) BPNet predictions over chrM; (C) HEK293 ChIP-seq (ENCODE ID ENCSR000EVD; antibody: Novus H00010127-A01); (D) HepG2 CETCH-seq.

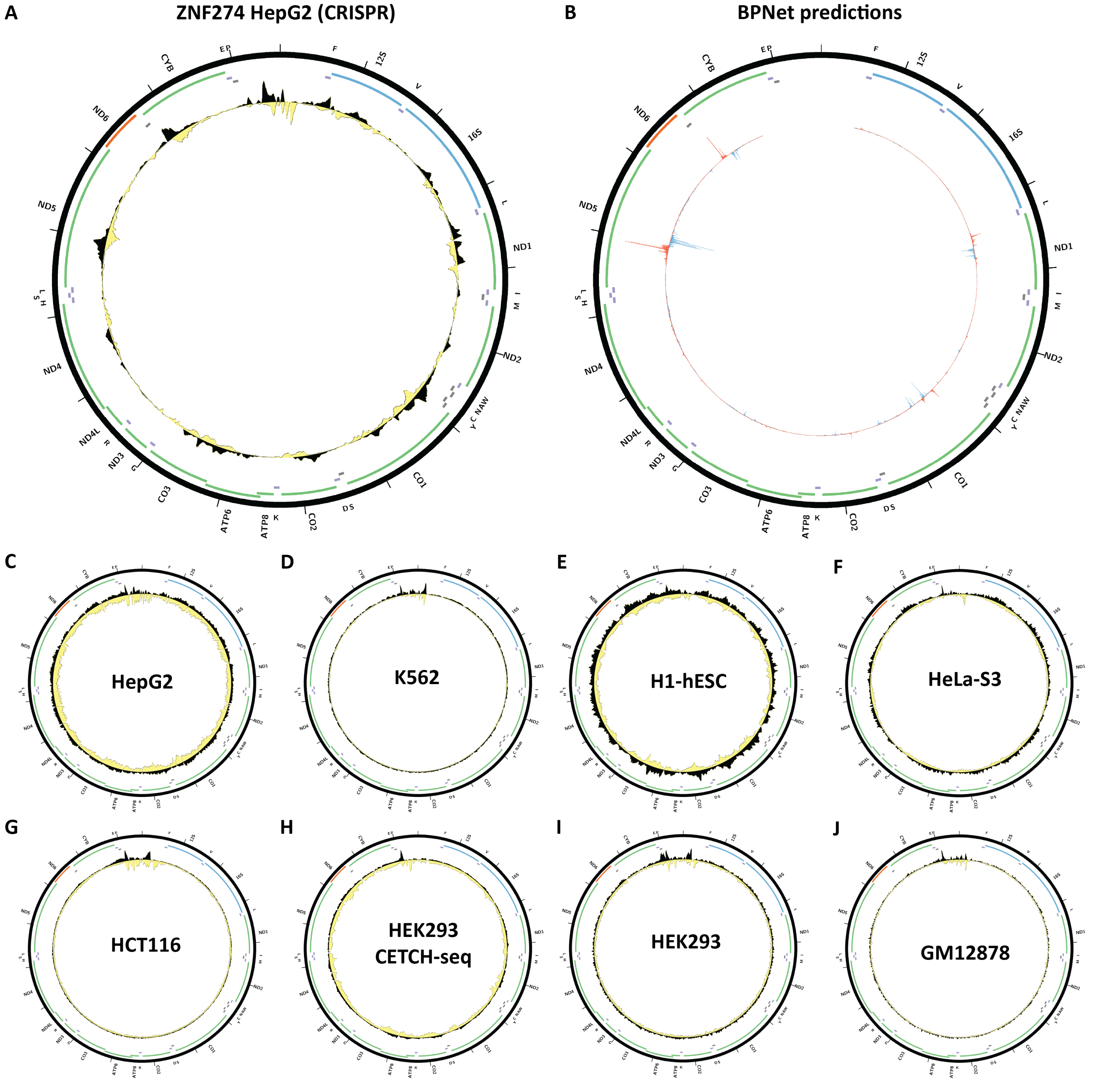

Supplementary Figure 37: Evidence for mitochondrial genome occupancy by the ZNF274 transcription factor. Black and yellow tracks show the forward- and reverse-strand ChIP-seq coverage over chrM. (A) HepG2 CETCH-seq (ENCODE ID ENCSR871VNN); (B) BPNet predictions over chrM; (C) HepG2 ChIP-seq (ENCODE ID ENCSR000EVR; antibody: Abnova H00010782-A01, Lot ID 060729QCS1); (D) K562 ChIP-seq (ENCODE ID ENCSR000EVX; antibody: Abnova H00010782-M01, Lot ID 08064-4C12); (E) H1-hESC ChIP-seq (ENCODE ID ENCSR000EUN; antibody: Abnova H00010782-M01, Lot ID 08064-4C12); (F) HeLa-S3 ChIP-seq (ENCODE ID ENCSR000EVG; antibody: Abnova H00010782-A01, Lot ID 060729QCS1); (G) HCT116 ChIP-seq (ENCODE ID ENCSR101FJM; antibody: Abnova H00010782-A01, Lot ID 060729QCS1); (H) HEK293 CETCH-seq (ENCODE ID ENCSR178QVJ); (I) HEK293 ChIP-seq (ENCODE ID ENCSR000FCI; antibody: Abnova H00010782-A01, Lot ID 060729QCS1); (J) GM12878 ChIP-seq (ENCODE ID ENCSR000EUK; antibody: Abnova H00010782-A01, Lot ID 060729QCS1).

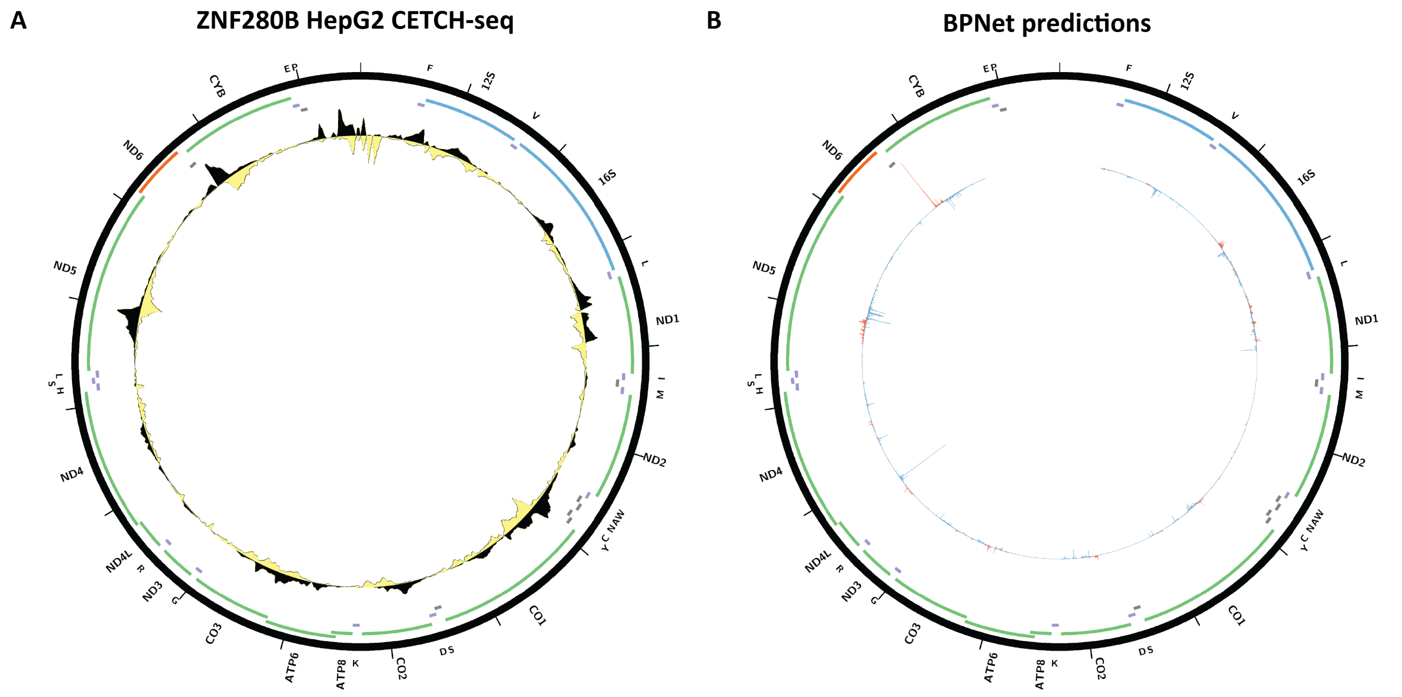

Supplementary Figure 38: Evidence for mitochondrial genome occupancy by the ZNF280B transcription factor. Black and yellow tracks show the forward- and reverse-strand ChIP-seq coverage over chrM. (A) HepG2 CETCH-seq (ENCODE ID ENCSR940EZR); (B) BPNet predictions over chrM.

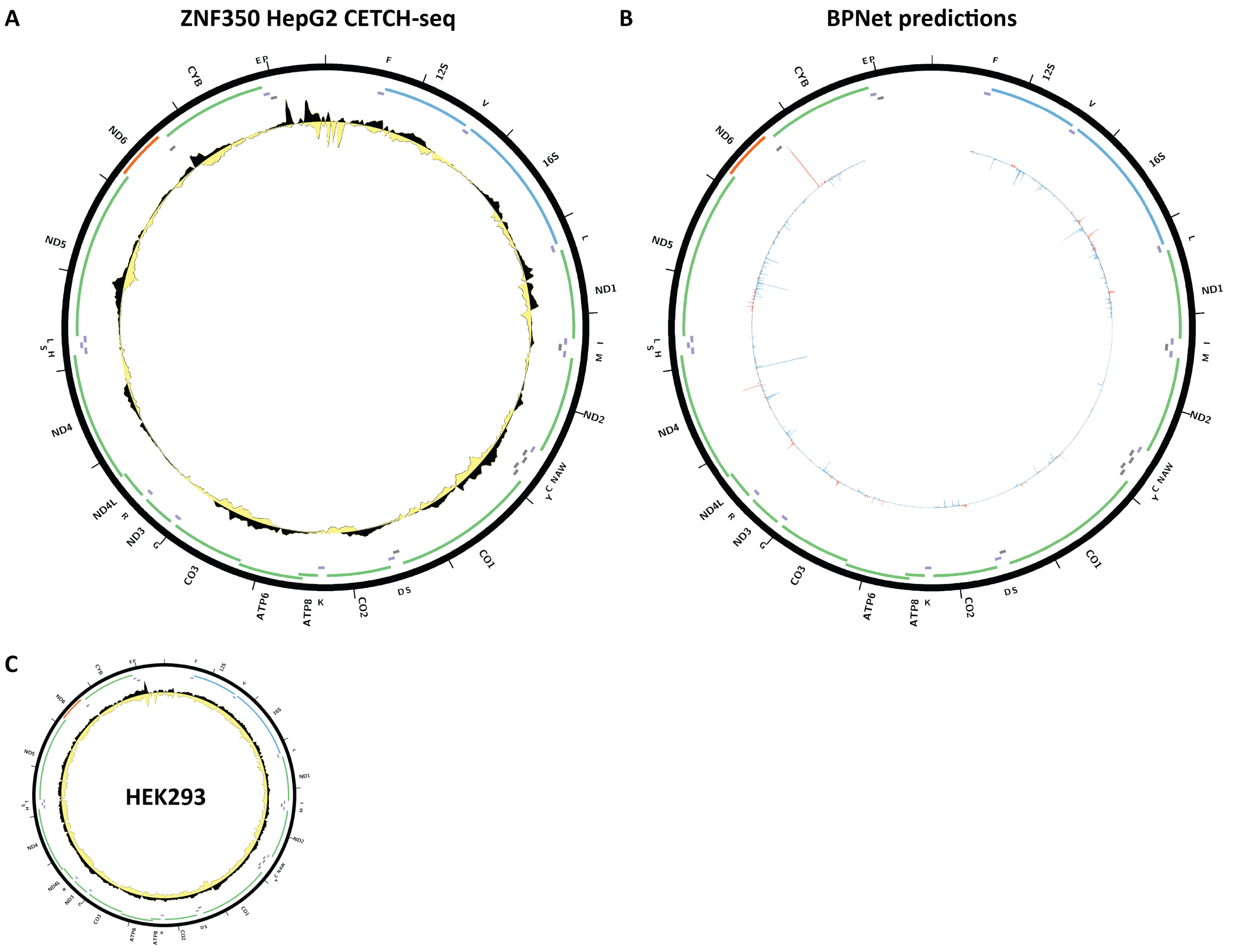

Supplementary Figure 39: Evidence for mitochondrial genome occupancy by the ZNF350 transcription factor. Black and yellow tracks show the forward- and reverse-strand ChIP-seq coverage over chrM. (A) HepG2 CETCH-seq (ENCODE ID ENCSR842SRB); (B) BPNet predictions over chrM; (C) HEK293 CETCH-seq (ENCODE ID ENCSR854ORP).

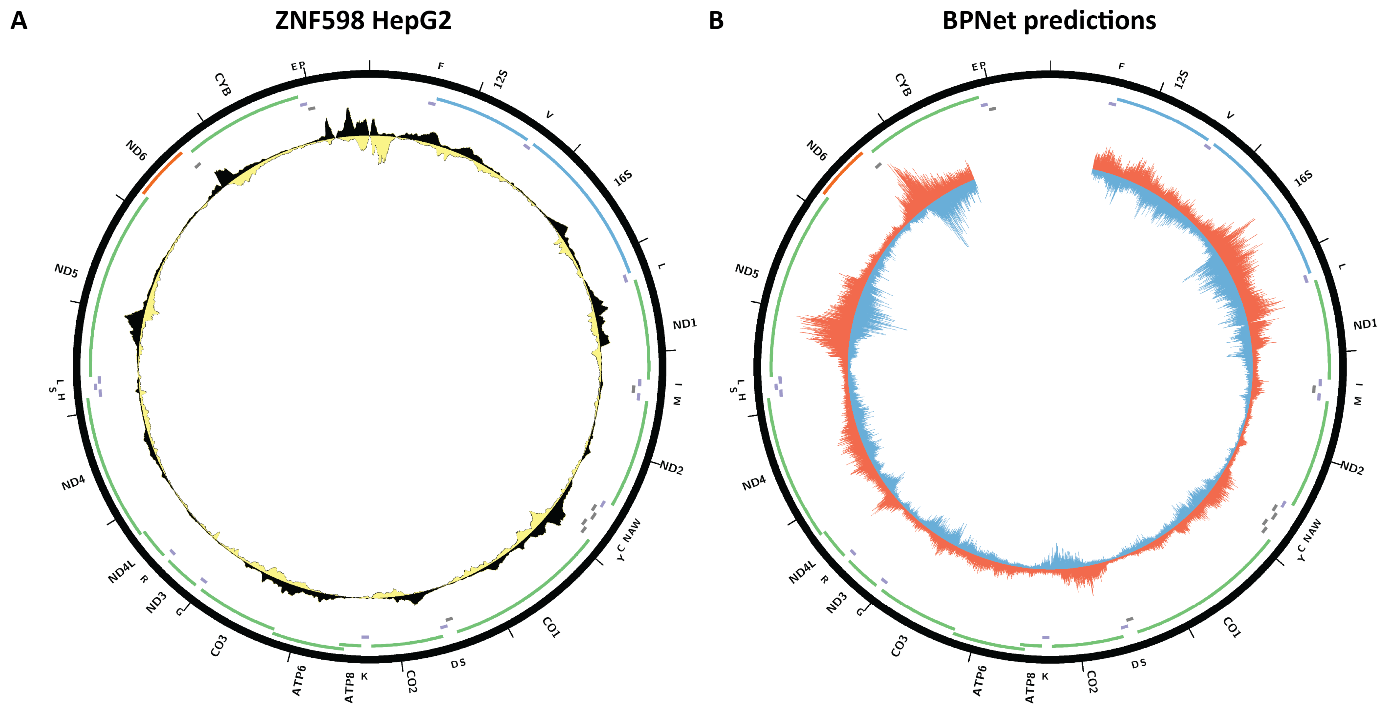

Supplementary Figure 40: Evidence for mitochondrial genome occupancy by the ZNF598 transcription factor. Black and yellow tracks show the forward- and reverse-strand ChIP-seq coverage over chrM. (A) HepG2 ChIP-seq (ENCODE ID ENCSR173NAL; antibody: Sigma F1804, Lot ID SLBK1346V); (B) BPNet predictions over chrM.

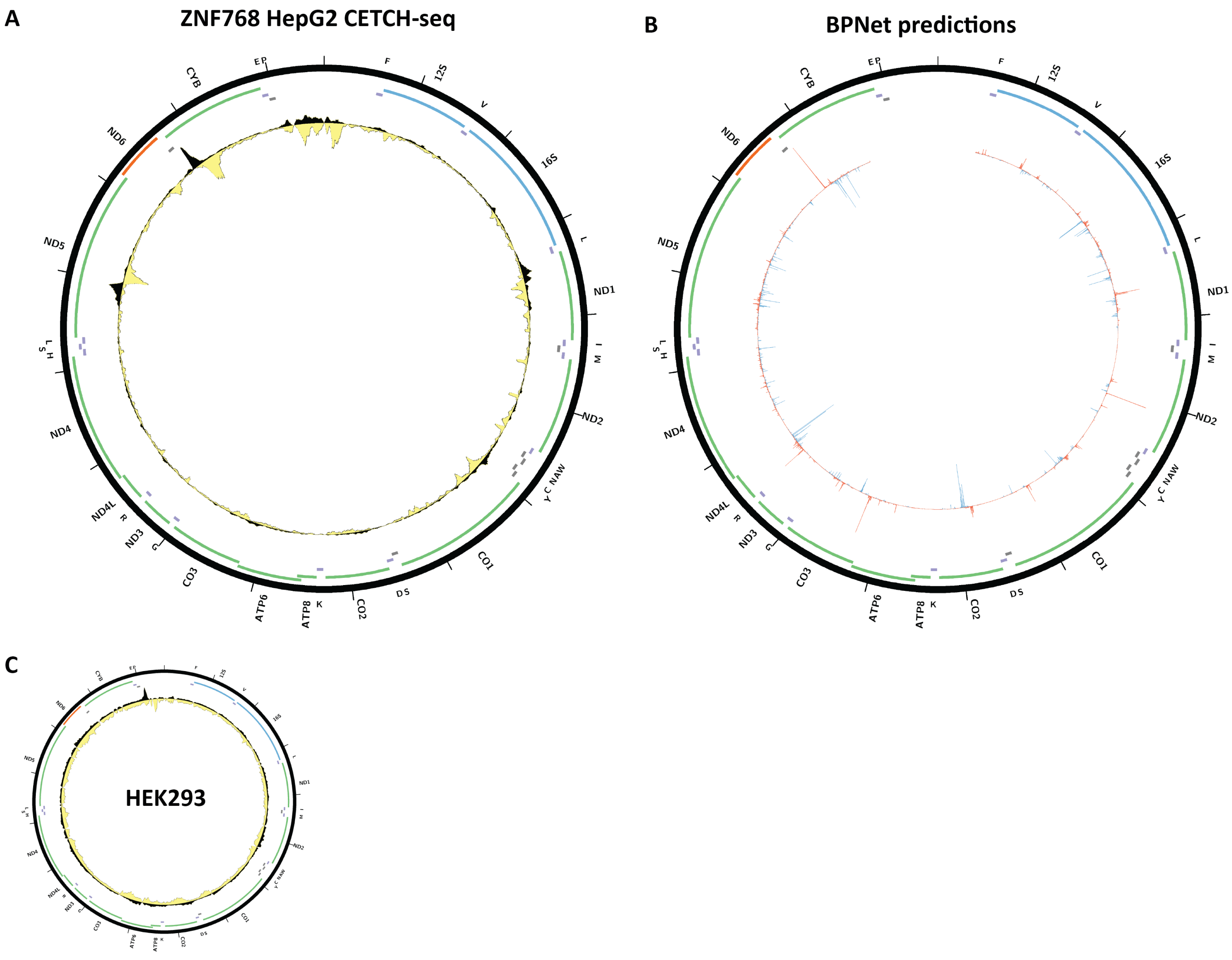

Supplementary Figure 41: Evidence for mitochondrial genome occupancy by the ZNF768 transcription factor. Black and yellow tracks show the forward- and reverse-strand ChIP-seq coverage over chrM. (A) HepG2 CETCH-seq (ENCODE ID ENCSR181ABP); (B) BPNet predictions over chrM; (C) HEK293 CETCH-seq (ENCODE ID ENCSR070HWF).

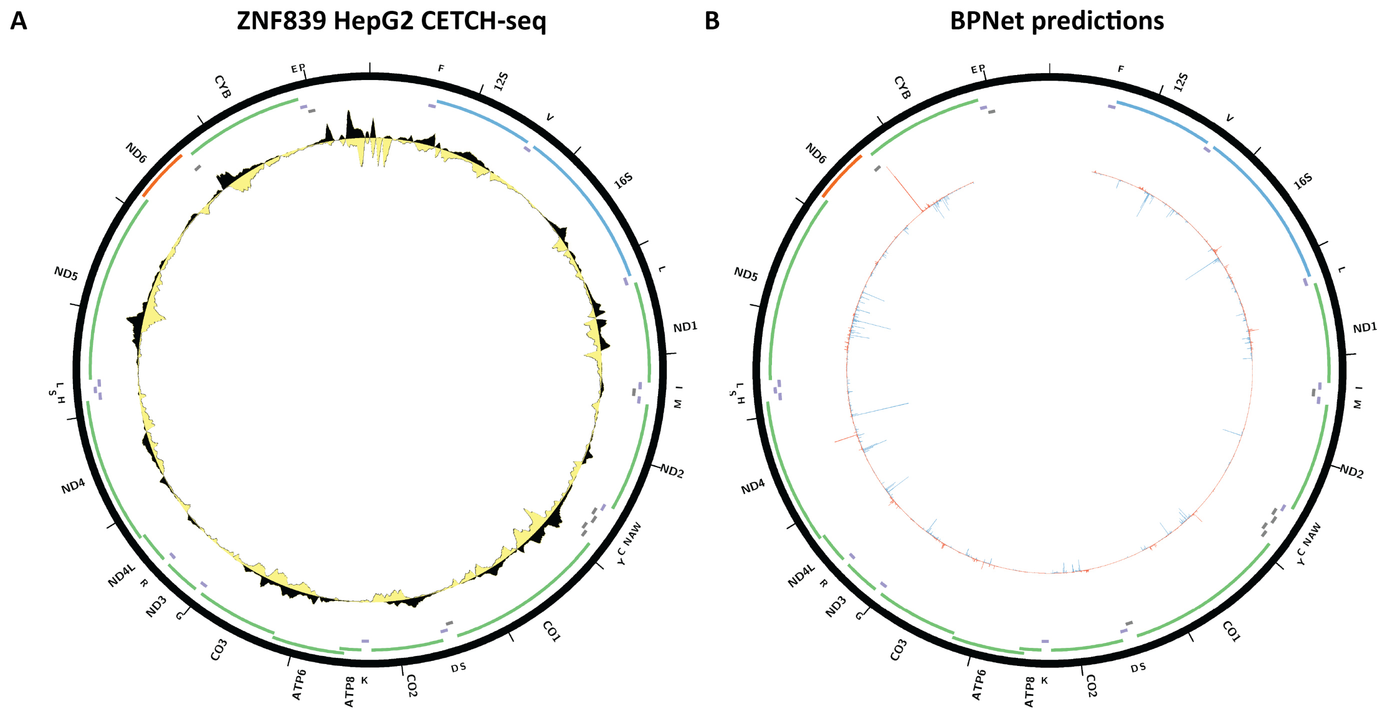

Supplementary Figure 42: Evidence for mitochondrial genome occupancy by the ZNF839 transcription factor. Black and yellow tracks show the forward- and reverse-strand ChIP-seq coverage over chrM. (A) HepG2 CETCH-seq (ENCODE ID ENCSR540LPD). (B) BPNet predictions over chrM.

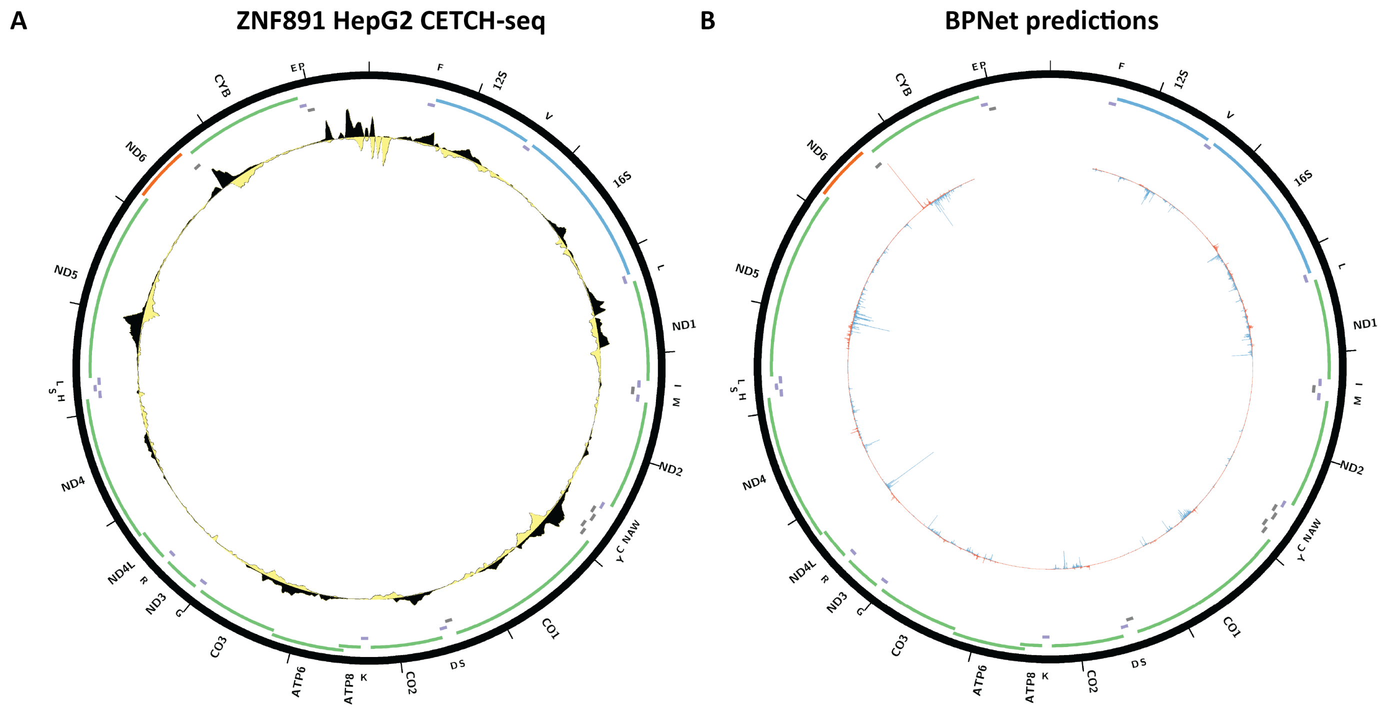

Supplementary Figure 43: Evidence for mitochondrial genome occupancy by the ZNF891 transcription factor. Black and yellow tracks show the forward- and reverse-strand ChIP-seq coverage over chrM. (A) HepG2 CETCH-seq (ENCODE ID ENCSR020CLV). (B) BPNet predictions over chrM.

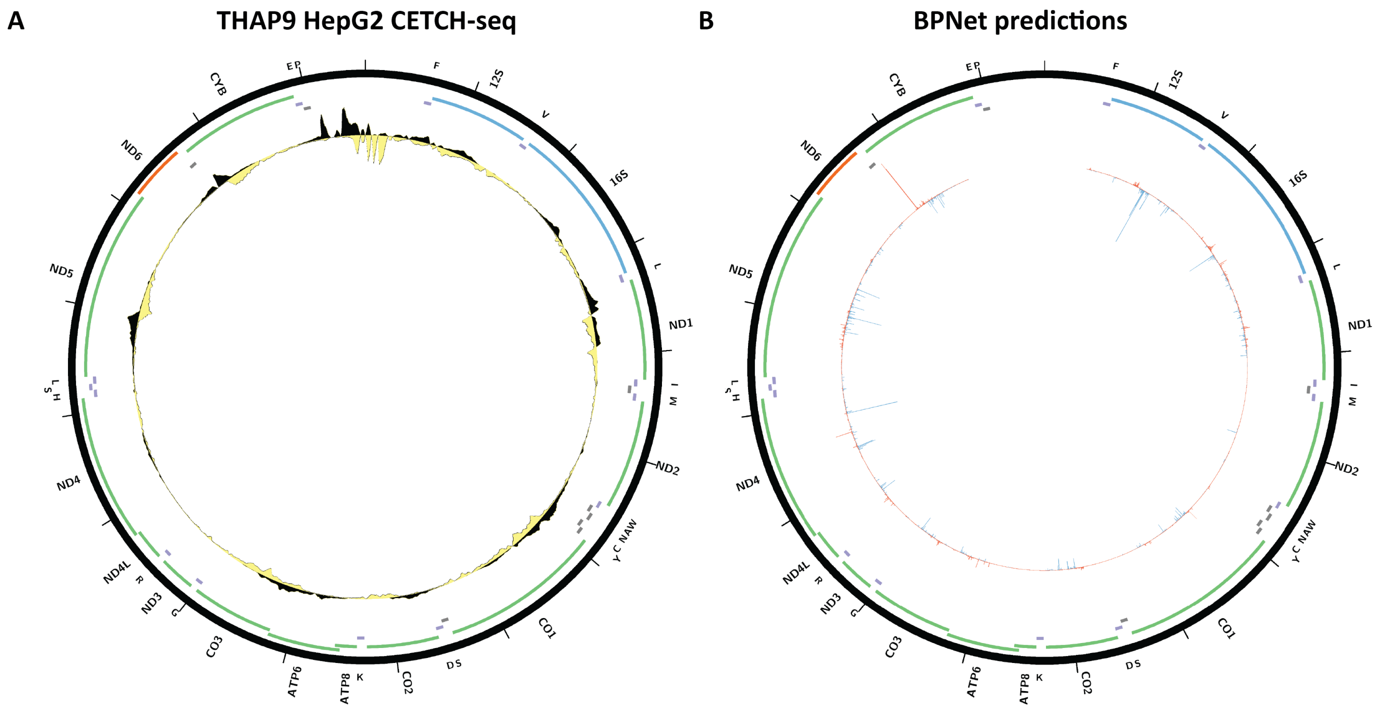

Supplementary Figure 44: Evidence for mitochondrial genome occupancy by the THAP9 transcription factor. Black and yellow tracks show the forward- and reverse-strand ChIP-seq coverage over chrM. (A) HepG2 CETCH-seq (ENCODE ID ENCSR123GPC); (B) BPNet predictions over chrM.

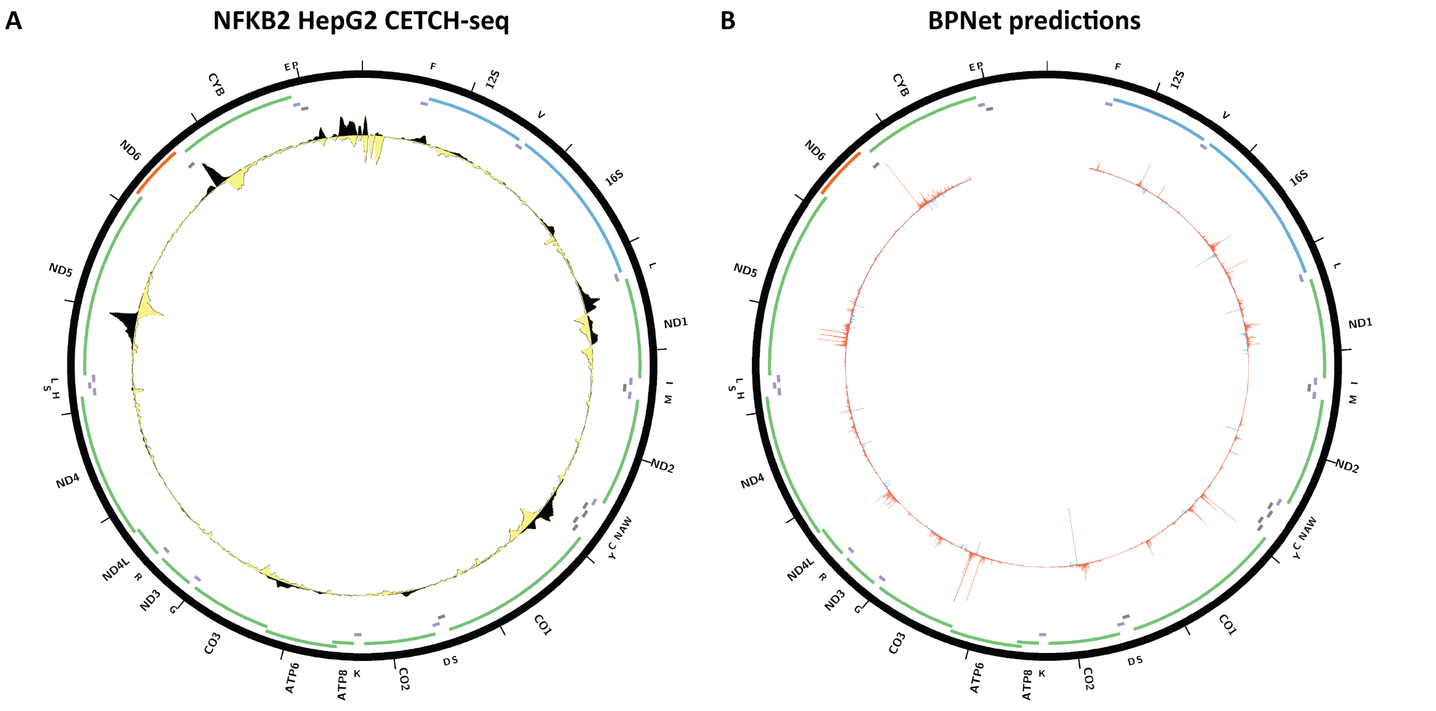

Supplementary Figure 45: Evidence for mitochondrial genome occupancy by the NFKB2 transcription factor. Black and yellow tracks show the forward- and reverse-strand ChIP-seq coverage over chrM. (A) HepG2 CETCH-seq (ENCODE ID ENCSR164YJZ); (B) BPNet predictions over chrM.

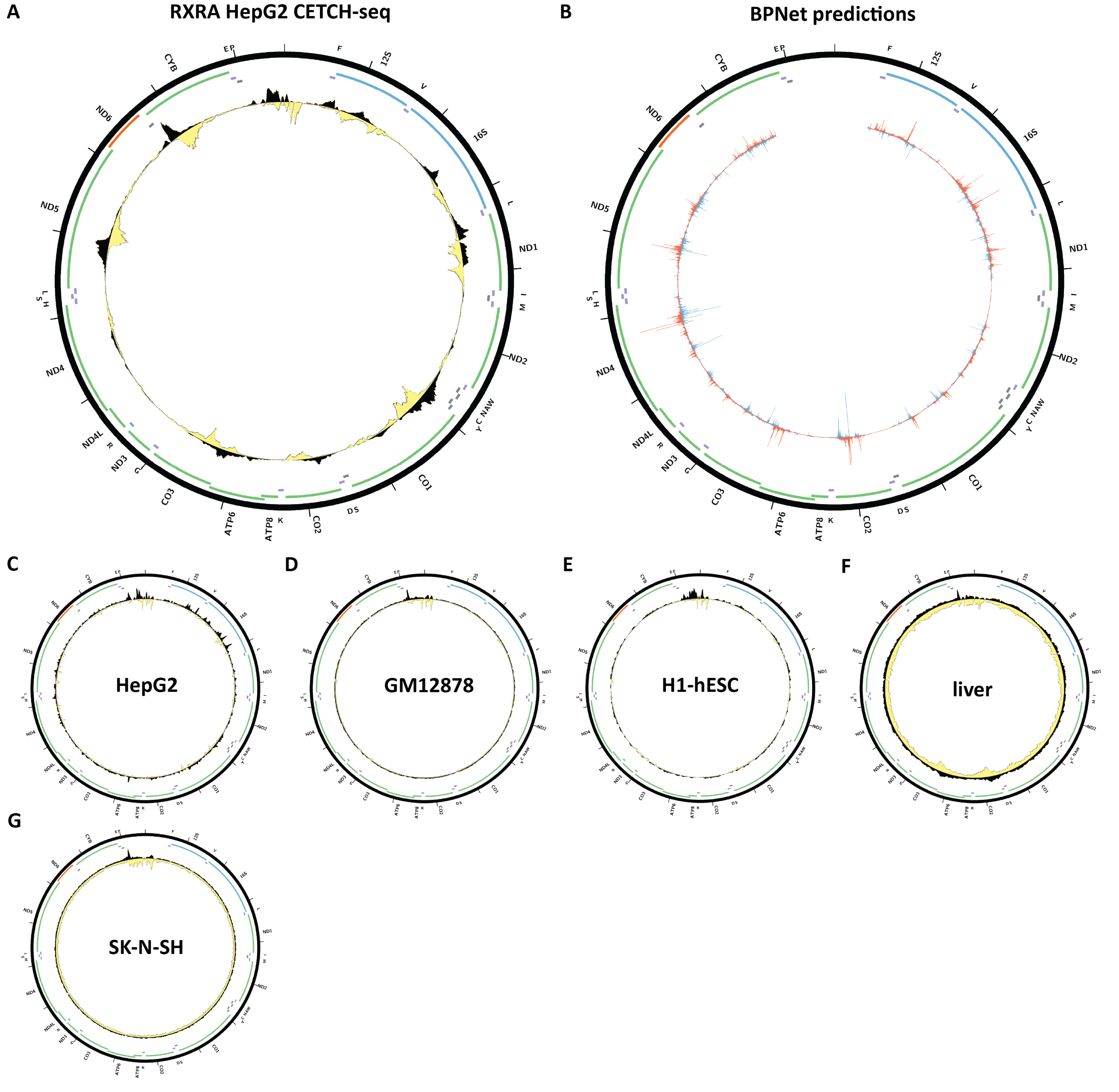

Supplementary Figure 46: Evidence for mitochondrial genome occupancy by the RXRA transcription factor. Black and yellow tracks show the forward- and reverse-strand ChIP-seq coverage over chrM. (A) HepG2 CETCH-seq (ENCODE ID ENCSR416HDG); (B) BPNet predictions over chrM (ENCODE ID ENCSR471TML); (C) HepG2 ChIP-seq (ENCODE ID ENCSR000BHU; antibody: Santa Cruz Biotech sc-553, Lot ID C1811); (D) GM12878 ChIP-seq (ENCODE ID ENCSR000BJD; antibody: Santa Cruz Biotech sc-553, Lot ID C1811); (E) H1-hESC ChIP-seq (ENCODE ID ENCSR000BJW; antibody: Santa Cruz Biotech sc-553, Lot ID C1811); (F) Liver ChIP-seq (ENCODE ID ENCSR352QSB; antibody: Santa Cruz Biotech sc-553, Lot ID C1811); (G) SK-N-SH ChIP-seq (ENCODE ID ENCSR000BVG; antibody: Santa Cruz Biotech sc-553, Lot ID C1811).

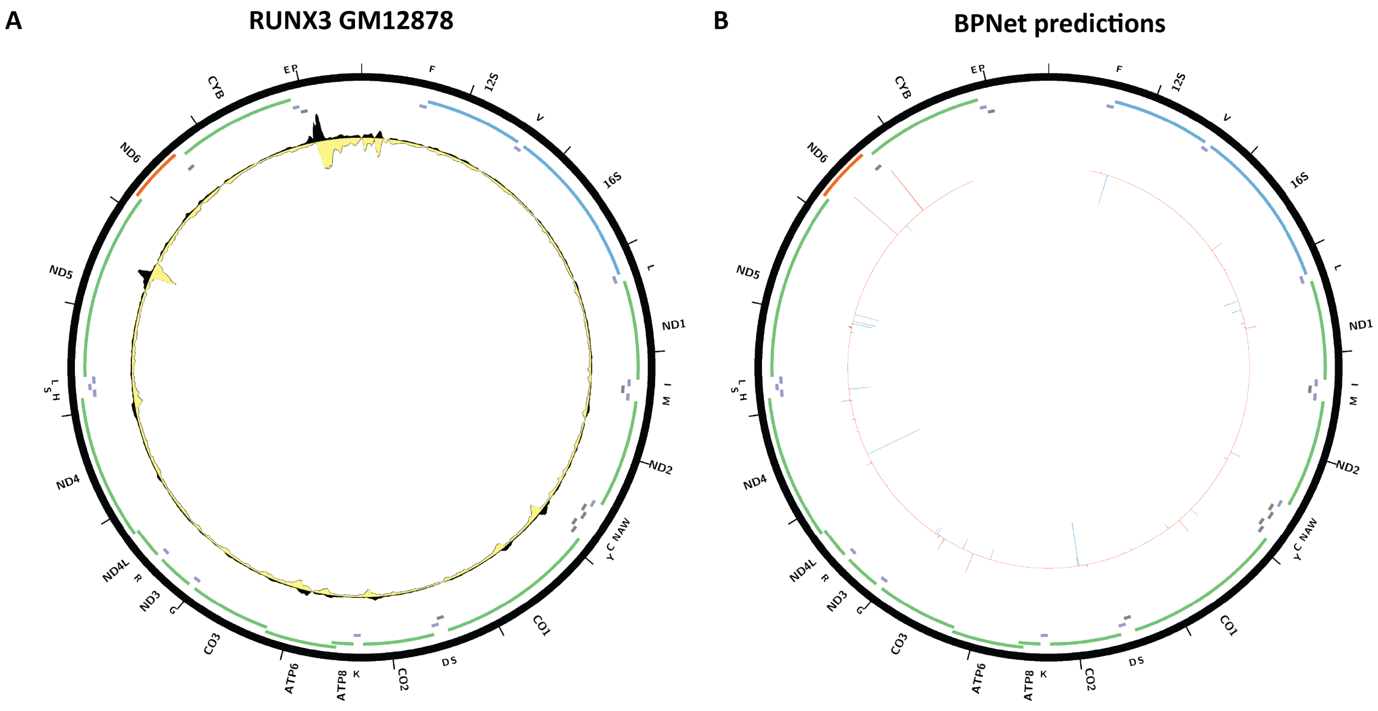

Supplementary Figure 47: Evidence for mitochondrial genome occupancy by the RUNX3 transcription factor. Black and yellow tracks show the forward- and reverse-strand ChIP-seq coverage over chrM. (A) GM12878 ChIP-seq (ENCODE ID ENCSR000BRI; antibody: Santa Cruz Biotech sc-101553, Lot ID B0909); (B) BPNet predictions over chrM (ENCODE ID ENCSR130BYX).

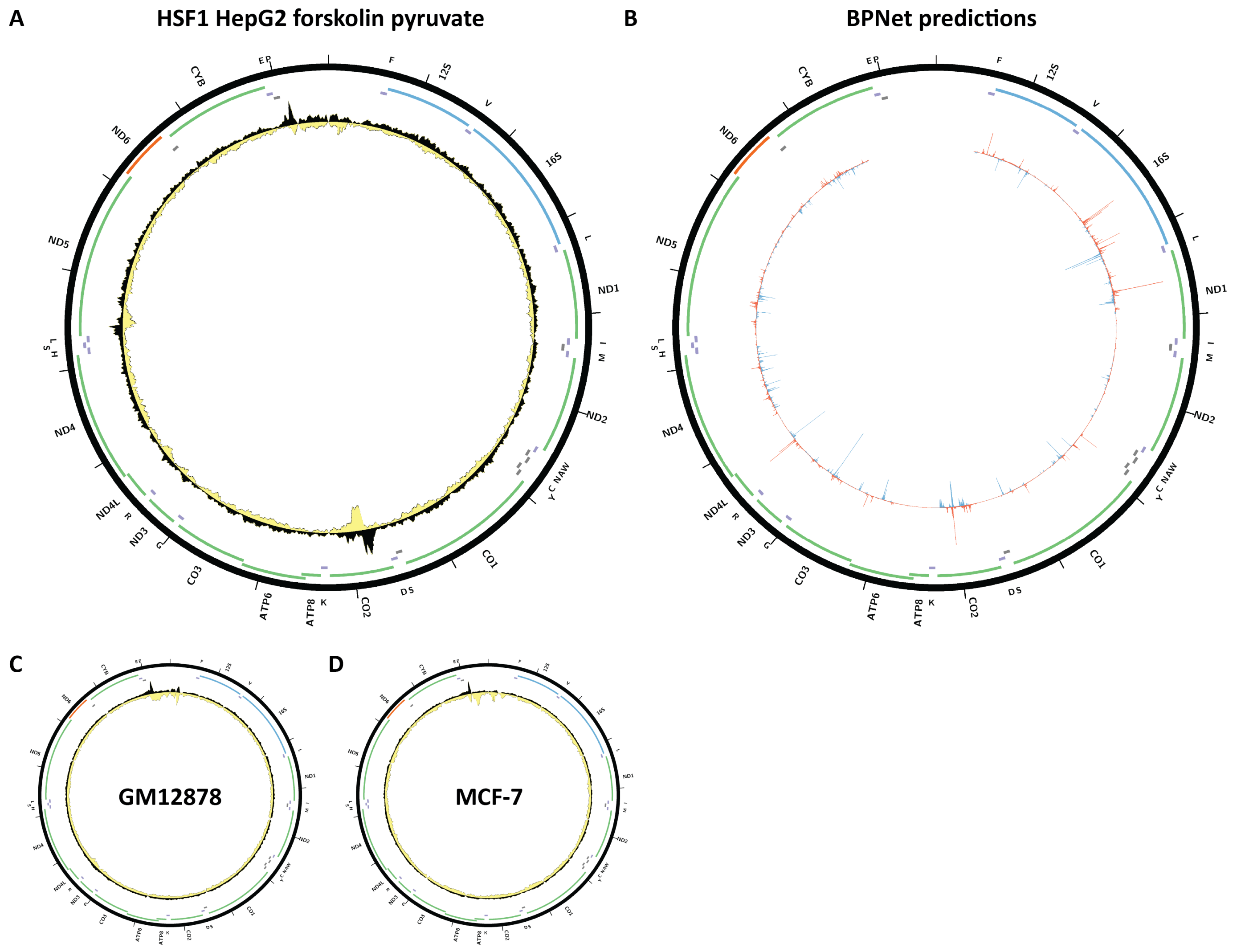

Supplementary Figure 48: Evidence for mitochondrial genome occupancy by the HSF1 transcription factor. Black and yellow tracks show the forward- and reverse-strand ChIP-seq coverage over chrM. (A) HepG2 forskolin + 1mM pyruvate 6 hours, ChIP-seq (ENCODE ID ENCSR000EET; antibody: Santa Cruz Biotech sc-9144); (B) BPNet predictions over chrM (ENCODE ID ENCSR000EET); (C) GM12878 ChIP-seq (ENCODE ID ENCSR009MBP; antibody: Santa Cruz Biotech sc-9144); (D) MCF-7 ChIP-seq (ENCODE ID ENCSR062HDL; antibody: Santa Cruz Biotech sc-9144).

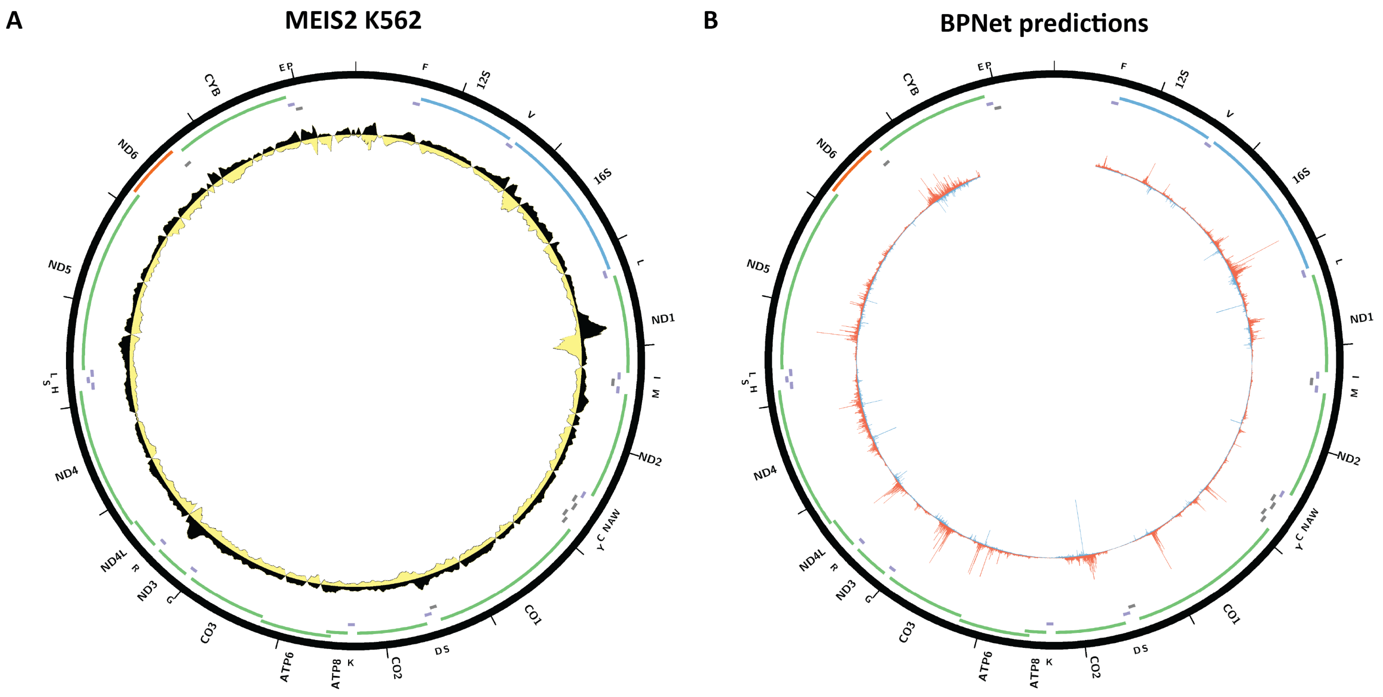

Supplementary Figure 49: Evidence for mitochondrial genome occupancy by the MEIS2 transcription factor. Black and yellow tracks show the forward- and reverse-strand ChIP-seq coverage over chrM. (A) K562 ChIP-seq (ENCODE ID ENCSR851BNE; antibody: Sigma HPA003256, Lot ID R89735); (B) BPNet predictions over chrM (ENCODE ID ENCSR603QNK).

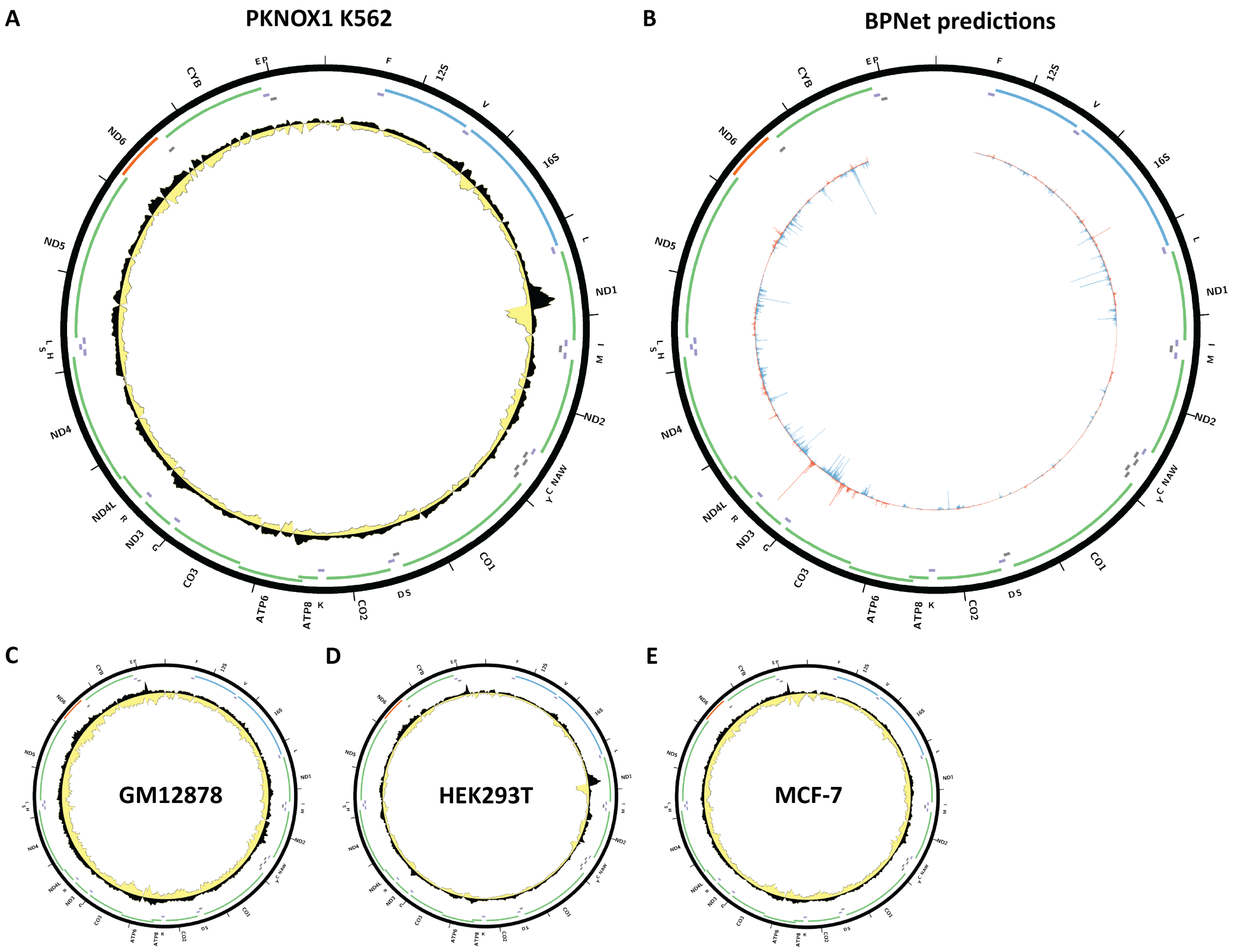

Supplementary Figure 50: Evidence for mitochondrial genome occupancy by the PKNOX1 transcription factor. Black and yellow tracks show the forward- and reverse-strand ChIP-seq coverage over chrM. (A) K562 ChIP-seq (ENCODE ID ENCSR115SMW; antibody: GeneTex GTX114991, Lot ID 40870); (B) BPNet predictions over chrM (ENCODE ID ENCSR509JOV); (C) GM12878 ChIP-seq (ENCODE ID ENCSR711XNY; antibody: GeneTex GTX114991, Lot ID 40870); (D) HEK293T ChIP-seq (ENCODE ID ENCSR233FAG; antibody: GeneTex GTX114991, Lot ID 40870); (E) MCF-7 ChIP-seq (ENCODE ID ENCSR986XYK; antibody: GeneTex GTX114991, Lot ID 40870).

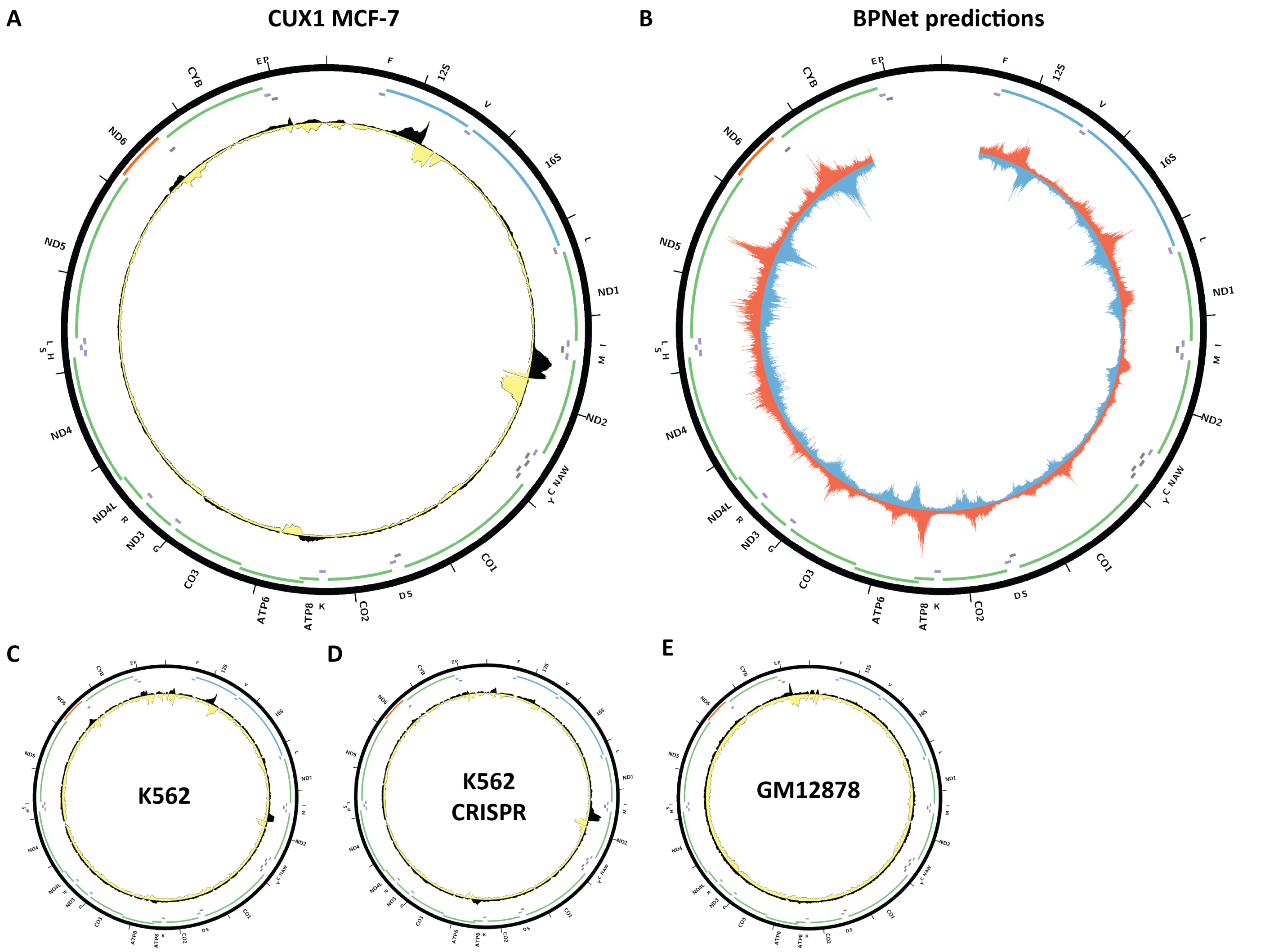

Supplementary Figure 51: Evidence for mitochondrial genome occupancy by the CUX1 transcription factor. Black and yellow tracks show the forward- and reverse-strand ChIP-seq coverage over chrM. (A) MCF-7 ChIP-seq (ENCODE ID ENCSR017CEO; antibody: Santa Cruz Biotech sc-6327, Lot ID E0709); (B) BPNet predictions over chrM (ENCODE ID ENCSR867JJN); (C) K562 ChIP-seq (ENCODE ID ENCSR000EFO; antibody: Santa Cruz Biotech sc-6327, Lot ID E0709); (D) K562 CETCH-seq (ENCODE ID ENCSR178NTX); (E) GM12878 ChIP-seq (ENCODE ID ENCSR000DYR; antibody: Santa Cruz Biotech sc-6327, Lot ID E0709).

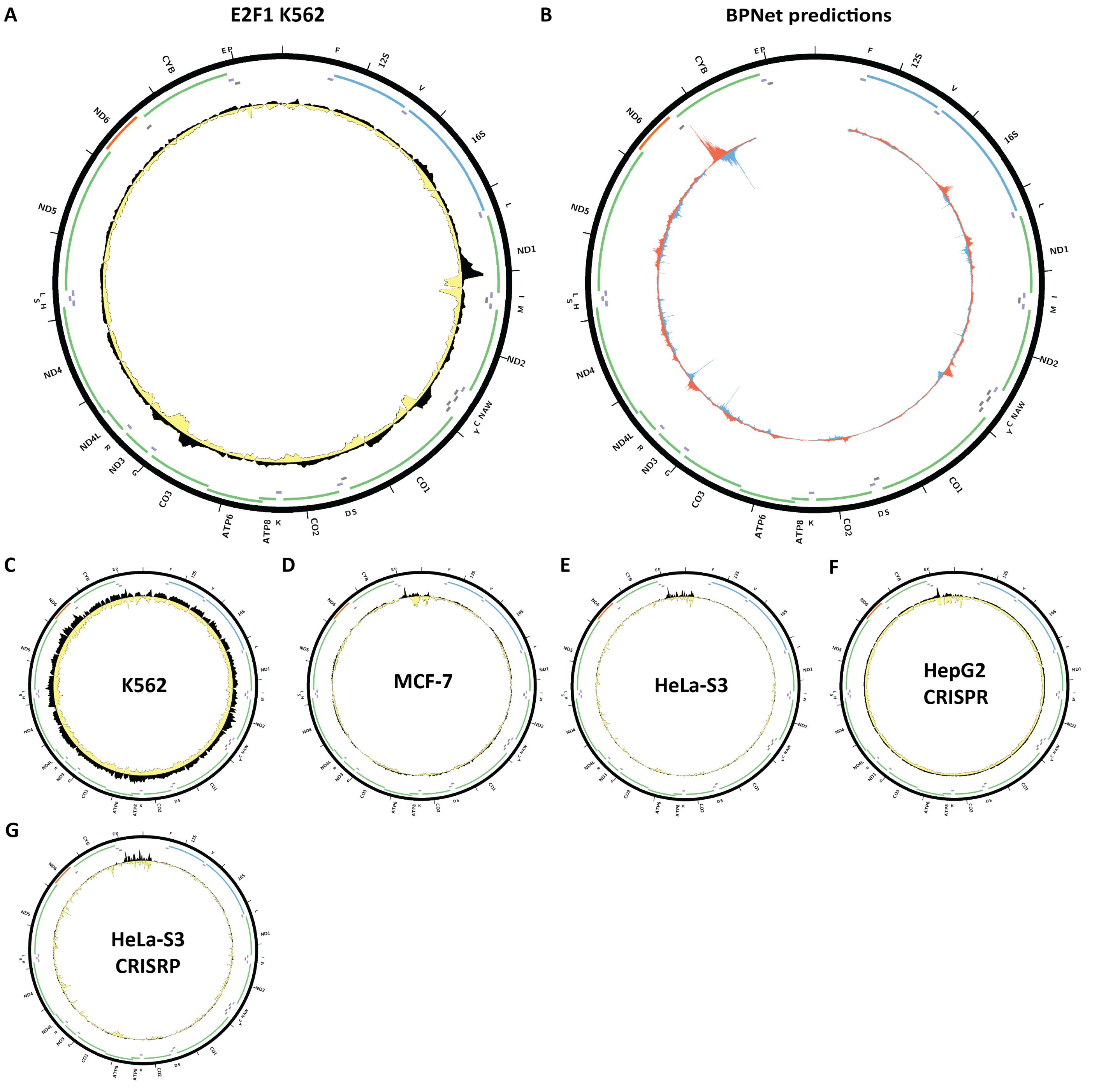

Supplementary Figure 52: Evidence for mitochondrial genome occupancy by the E2F1 transcription factor. Black and yellow tracks show the forward- and reverse-strand ChIP-seq coverage over chrM. (A) K562 ChIP-seq (ENCODE ID ENCSR720HUL; antibody: GeneTex GTX70165, Lot ID 19267); (B) BPNet predictions over chrM; (C) K562 ChIP-seq (ENCODE ID ENCSR563LLO; antibody: Cell Signaling 3742S, Lot ID 4); (D) MCF-7 ChIP-seq (ENCODE ID ENCSR000EWX; HA-modified E2F1); (E) HeLa-S3 ChIP-seq (ENCODE ID ENCSR000EVJ; antibody: Millipore 05-379). (F) HepG2 CETCH-seq (ENCODE ID ENCSR717ZZW); (G) HeLa-S3 CETCH-seq (ENCODE ID ENCSR000EVM).

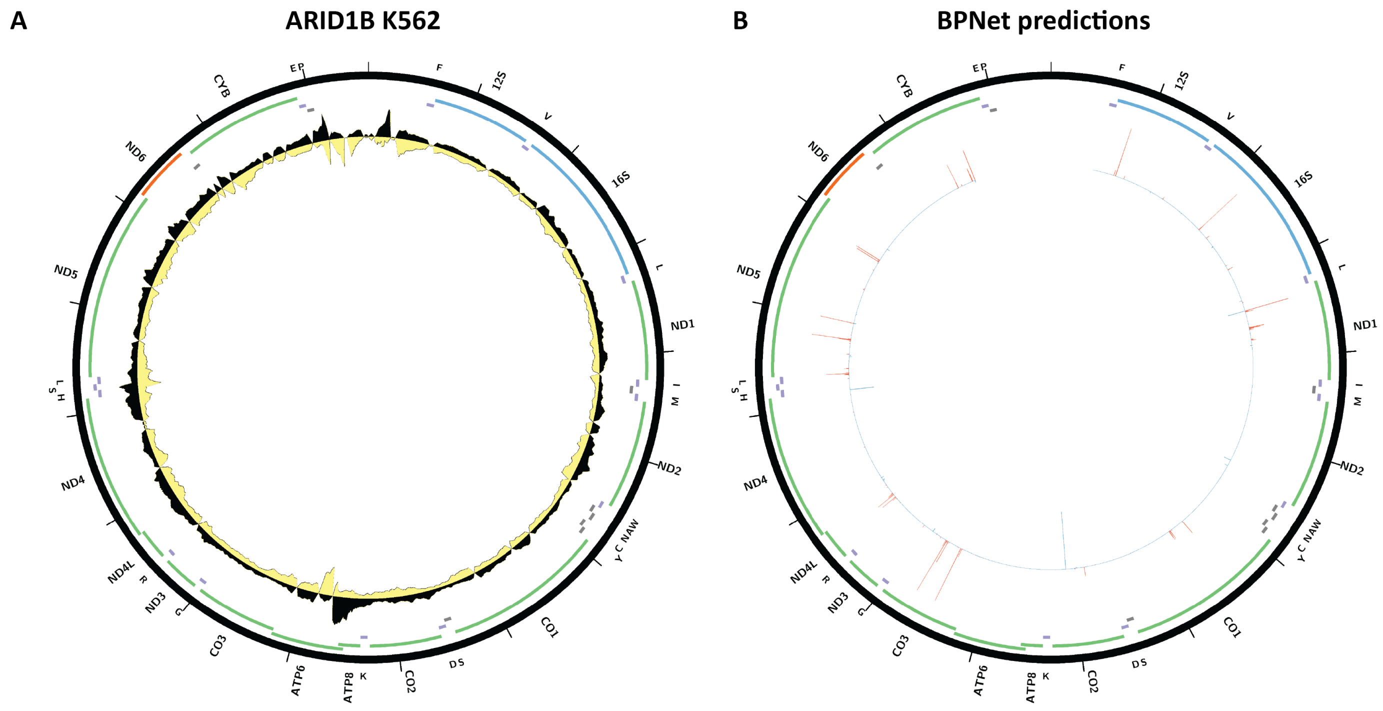

Supplementary Figure 53: Evidence for mitochondrial genome occupancy by the ARID1B transcription factor. Black and yellow tracks show the forward- and reverse-strand ChIP-seq coverage over chrM. (A) K562 ChIP-seq (ENCODE ID ENCSR822CCM; antibody: Bethyl Labs A301-046A); (B) BPNet predictions over chrM.

MCF-7 TFAP2C CETCH-seq

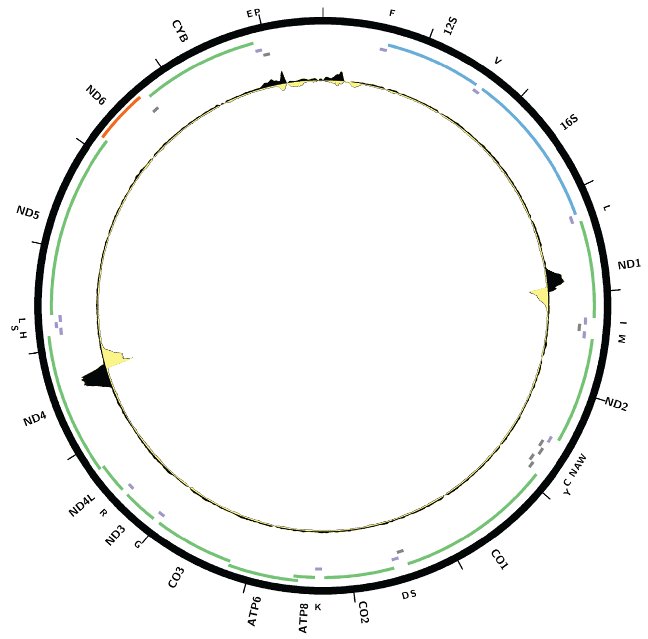

Supplementary Figure 54: Evidence for mitochondrial genome occupancy by the TFAP2C transcription factor. Black and yellow tracks show the forward- and reverse-strand ChIP-seq coverage over chrM. CETCH-seq; ENCODE ID ENCSR742RUA.

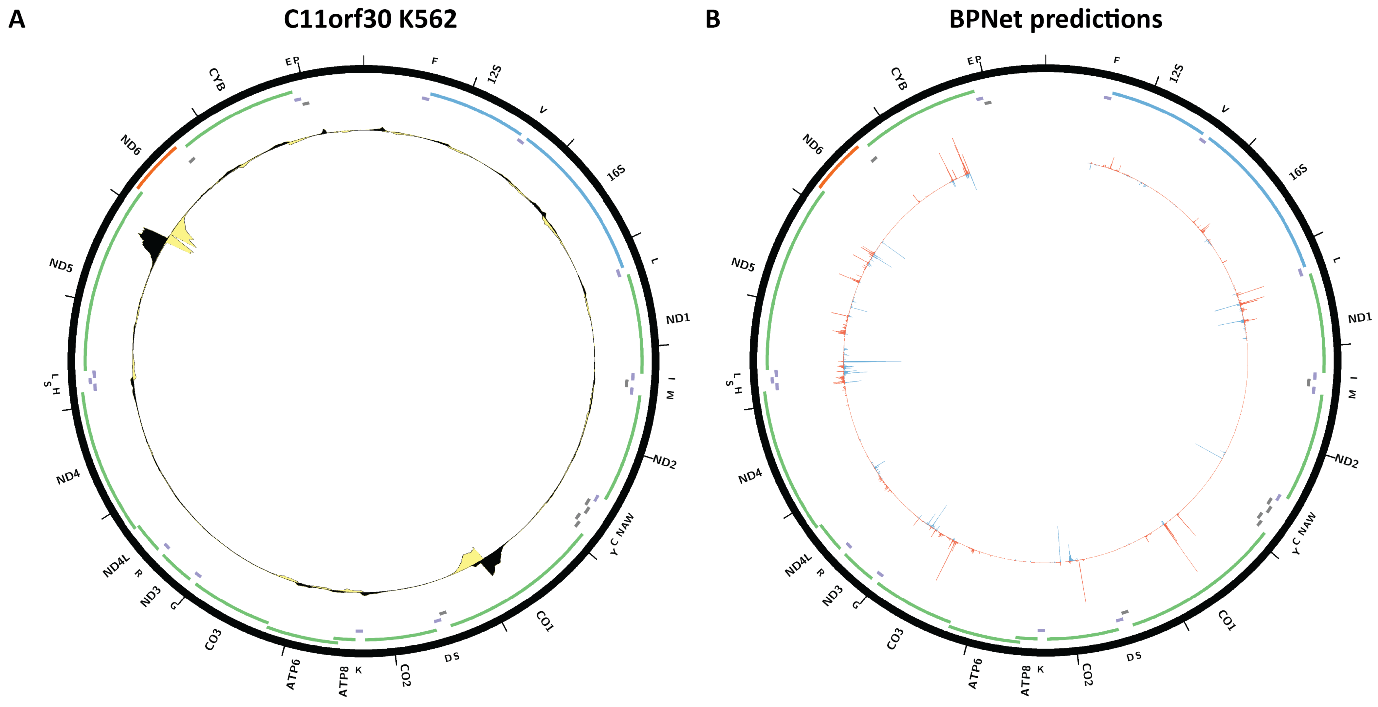

Supplementary Figure 55: Evidence for mitochondrial genome occupancy by the C11orf30 protein. Black and yellow tracks show the forward- and reverse-strand ChIP-seq coverage over chrM. (A) K562 ChIP-seq (ENCODE ID ENCSR350XWY; antibody: Bethyl Labs A300-253A, Lot ID 2); (B) BPNet predictions over chrM (ENCODE ID ENCSR350XWY).

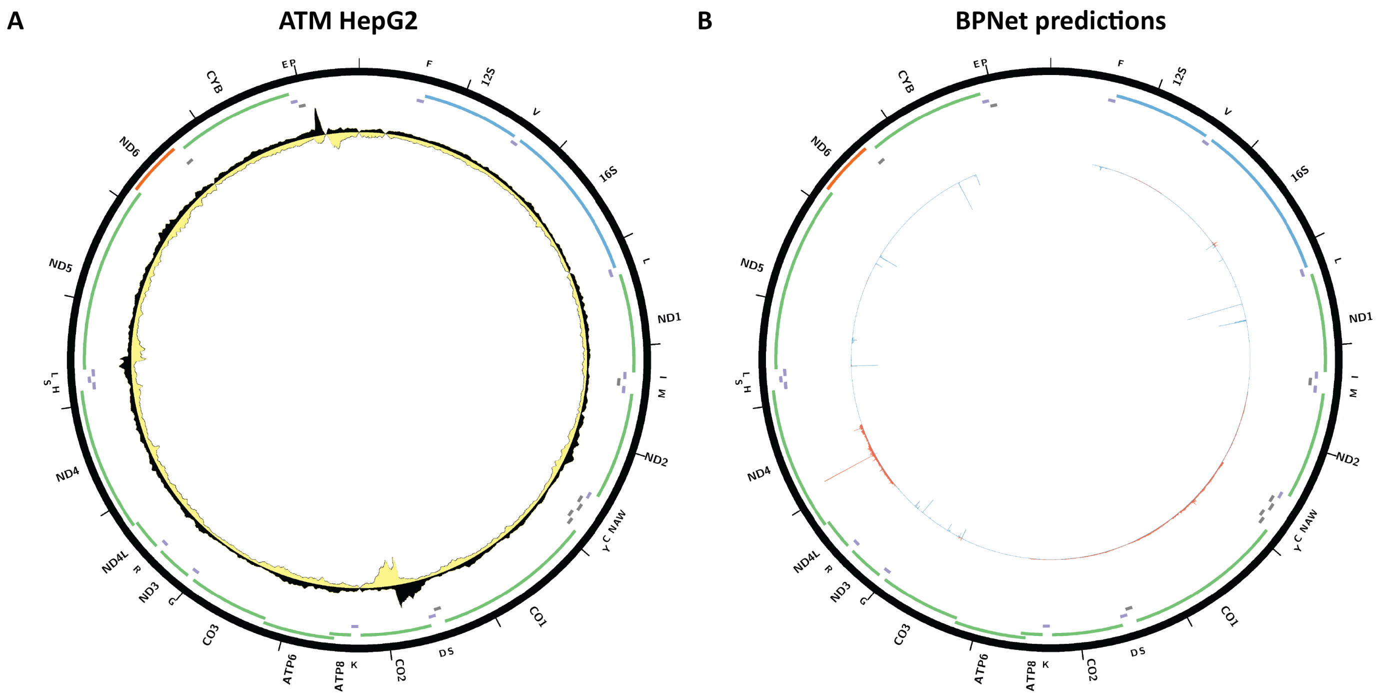

Supplementary Figure 56: Evidence for mitochondrial genome occupancy by the ATM protein. Black and yellow tracks show the forward- and reverse-strand ChIP-seq coverage over chrM. (A) HepG2 ChIP-seq (ENCODE ID ENCSR859JGF; antibody: Bethyl Labs A300-135A); (B) BPNet predictions over chrM.
